# Regioisomeric carbazole–dicyano–dioxin AIDF emitters for efficient triplet harvesting, two-photon absorption and bioimaging

**DOI:** 10.1039/d6sc03725a

**Published:** 2026-07-24

**Authors:** Anwesha Bera, Madhusudan Dutta, Ajay J. Malik, Madan D. Ambhore, Mayurika Lahiri, Partha Hazra

**Affiliations:** a Department of Chemistry, Indian Institute of Science Education and Research (IISER) Pune 411008 Maharashtra India p.hazra@iiserpune.ac.in; b Department of Biology, Indian Institute of Science Education and Research (IISER) Pune 411008 Maharashtra India; c Department of Chemistry, Yeshwant Mahavidyalaya Nanded Nanded 431602 Maharashtra India

## Abstract

Integrating aggregation-induced emission (AIE) with thermally activated delayed fluorescence (TADF) offers a useful strategy to achieve efficient triplet harvesting in the condensed phase. Here, we report three carbazole–dicyano–dioxin donor–acceptor regioisomers (*o*CN1, *o*CN2, and *p*CN) that exhibit pronounced aggregation-induced delayed fluorescence (AIDF) from solution to aggregates, neat films and crystals. Oxygen incorporation and peripheral *tert*-butyl groups make the molecular framework more rigid and help suppress aggregation caused quenching. These substitutions also promote twisted donor–acceptor geometries. In addition, subtle *ortho*/*para* changes in connectivity tune the charge-transfer character, the singlet–triplet energy gaps, and the reverse intersystem crossing dynamics. In THF-water mixtures, all isomers display clear AIDF signatures, with PLQYs up to 63% and microsecond delayed lifetimes, enabled by up to ∼20-fold suppression of internal conversion (IC) upon aggregation. In neat films, all these isomers exhibit bright green to yellow-orange emission with PLQYs of 78%, 31% and 54% for *o*CN1, *o*CN2, and *p*CN, respectively. Moreover, all three regioisomers exhibit an RISC rate of ∼10^5^ s^−1^ in neat films. This fast RISC rate is attributed to the small singlet–triplet energy gap and appreciable spin–orbit coupling. The strong charge-transfer character and robust AIDF further enable efficient two-photon bioimaging and the fabrication of converted LEDs.

## Abstract 2

Three carbazole–dicyano–dioxin regioisomers exhibits AIDF with high PLQYs, fast RISC (∼10⁵ s^−1^) and tunable emission. Efficient triplet harvesting and strong charge-transfer character enable applications in converted LEDs and two-photon bioimaging.

## Graphical abstract

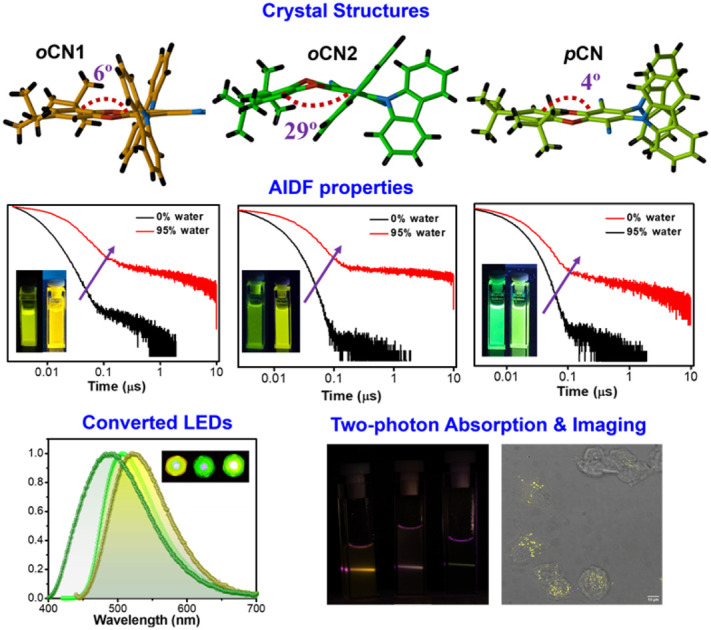

## Introduction

Organic luminescent materials that exhibit efficient solid-state emission are potential candidates for optoelectronic applications such as light-emitting diodes (OLEDs)^1–3^ as well as bioimaging, anticounterfeiting, photocatalysis, and sensing.^4–7^ Conventional luminophores show efficient light-emitting performance in dilute solution; however, their emission is drastically quenched or suppressed in the aggregated or solid state. This is due to the aggregation caused quenching (ACQ) effect, where strong π–π interactions increase the nonradiative deactivation channel through excitonic coupling, excimer formation, and energy transfer.^8^ To mitigate ACQ in OLEDs, the emissive materials are often diluted in a suitable host matrix, which limits the development of nondoped OLEDs.^9–12^ Nevertheless, doped OLEDs often suffer from efficiency roll-off where high efficiency at low brightness quickly falls as luminance increases.^13^ This drop is mainly caused by exciton–exciton and exciton–polaron interactions, charge imbalance, and local heating at high current densities.^14,15^ In this context, selecting a suitable host material and precisely controlling the dopant concentration is challenging, which can lead to poor reproducibility and reduced stability in OLED devices.^16,17^ Therefore, developing new emitters that can operate efficiently in non-doped devices is highly desirable.

In 2001, the discovery of aggregation-induced emission (AIE) by Tang and co-workers provided a breakthrough strategy to overcome ACQ, demonstrating that AIE-active luminogens (AIEgens) are weakly emissive in solution but exhibit strong emission properties upon aggregation or in the solid state.^18,19^ AIE significantly boosts emission efficiency and suppresses nonradiative decay by restricting intramolecular motion, rotation, and vibration *via* twisting molecular geometry.^20^ As a result, AIEgens are promising candidates for efficient solid state emission and have been widely explored in non-doped OLEDs, where electroluminescence efficiency can approach the theoretical 25% limit of electrogenerated singlet excitons for fluorescent emitters, with minimal efficiency roll-off.^21–24^ However, the remaining 75% of electrically generated triplet excitons are not utilized, leading to a significant loss in device efficiency. To further improve the exciton utilization in such systems, it is essential to activate these non-emissive triplet states. Following the pioneering work of Adachi and co-workers in 2012, thermally activated delayed fluorescence (TADF) has emerged as the most promising triplet exciton harvesting strategy, enabling up to 100% internal quantum efficiency (IQE) by recycling the non-emissive 75% triplet excitons *via* reverse intersystem crossing (RISC) without reliance on heavy metals.^1,25–28^ To achieve TADF, an effective RISC process requires a nearly degenerate or very small singlet–triplet energy gap (Δ*E*_ST_) together with sufficiently strong spin–orbit coupling. Donor–acceptor-based twisted molecular architectures are widely employed to fulfil these criteria, as they possess spatially separated the highest occupied molecular orbital (HOMO) and lowest unoccupied molecular orbital (LUMO), reduce electronic coupling, and thus afford a small Δ*E*_ST_.^26–28^ Thus, a logical next step to enhance the efficiency of non-doped OLEDs is to integrate AIE and TADF within a single molecular platform, wherein aggregation not only activates and strengthens emission but also facilitates efficient triplet–singlet up-conversion, a process known as aggregation-induced delayed fluorescence (AIDF). Guided by this design principle, Tang and co-workers reported a diverse array of AIDF materials consisting of carbazole analogues as donors and benzophenone or benzonitrile analogues as acceptors.^29–32^ Our group has also demonstrated that a series of triphenylamine-based emitters (TN, TA, and TP) exhibit AIDF properties, where molecular aggregation modulates the excited-state manifold to reduce the effective Δ*E*_ST_ and promote efficient delayed fluorescence.^33^ More recently, our group also illustrated that excited-state structural reorganization of molecular aggregates is a key factor governing this enhancement of solid state TADF, enabling the fabrication of highly efficient cyan-green and yellow AIDF-OLEDs.^34^

**Scheme 1:**
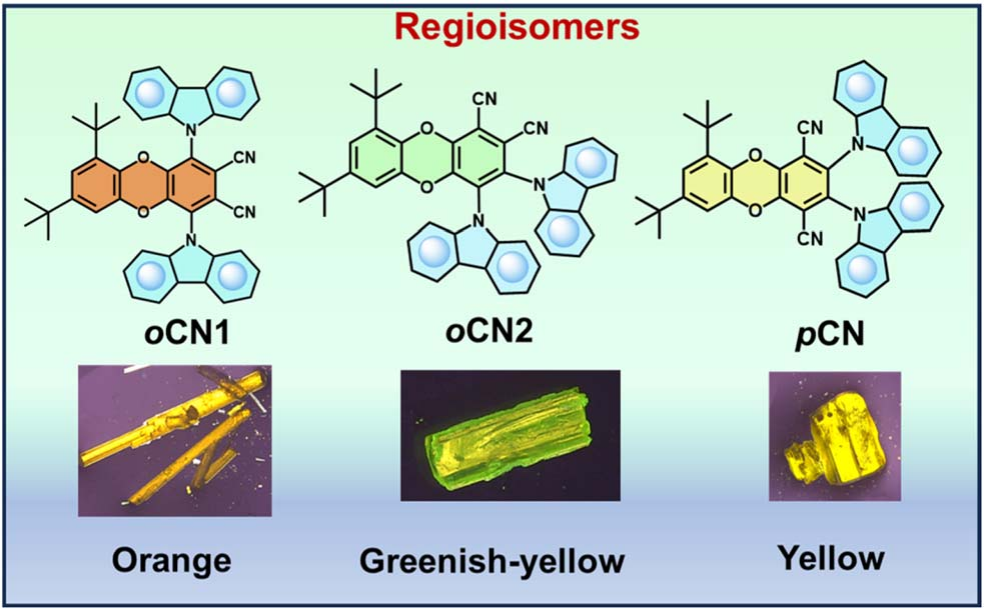
Molecular structures with three emitters with carbazole groups as donors and modified dibenzo-1,4-dioxine as an acceptor core (top). Tuneable emission of crystals under exposure to 365 nm UV light (bottom).

Despite these advancements in AIDF emitters, most reported systems still rely on conventional building blocks with closely related electronic configurations, which limits systematic tuning of their structure–property relationships. Therefore, it is highly desirable to explore new molecular architectures featuring rigid π-frameworks and heteroatom-rich cores. Such designs are expected to facilitate more efficient exciton utilization, suppress nonradiative decay pathways, and enhance solid-state PLQY, thereby paving the way for next-generation high-performance AIDF materials. Keeping the aforementioned challenges in mind, three regio-isomeric AIDF molecules have been designed and synthesized, namely *o*CN1, *o*CN2, and *p*CN, consisting of a modified dibenzo-1,4-dioxine (DBO) core as an acceptor and carbazole as a donor, as shown in Scheme 1. We believe that the introduction of *tert*-butyl groups on the DBO core disrupts face-to-face π–π intermolecular interactions between the DBO core in the crystal lattice, enhances solubility, and suppresses nonradiative decay channels, thereby improving the solid-state PLQY. In neat films, these emitters display tunable emission from green to yellow-orange with high solid state PLQYs of 31–78%, clearly evidencing their aggregation-induced emission characteristics. Notably, *o*CN2 exhibits a comparatively lower PLQY of 31%, which is attributed to pronounced face-to-face π–π stacking between the DBO core promoted by its puckered molecular geometry, indicating that specific crystal packing motifs can reopen non-radiative pathways and thereby modulate solid state emission efficiency. Their AIDF behaviour is further verified using delayed emission spectra and transient PL decay measurements upon going from THF solution to THF/water mixtures. Moreover, upon aggregation, the internal conversion rate (*k*_IC_) markedly decreases relative to the solution state, which facilitates spin flipping and results in pronounced delayed fluorescence and enhanced PLQY in the aggregated state. This suppression of IC is accompanied by a significant increase in the RISC rate, reaching 5.4 × 10^5^ s^−1^ for *o*CN1 and 3.6× 10^5^ and 4.2 × 10^5^ s^−1^ for *o*CN2 and *p*CN aggregates (95% water–THF mixture) respectively. Overall, our studies indicate that the observed structure–property correlation highlights the need for rational control of both molecular geometry and aggregate structure, which is crucial for developing next generation AIDF materials. Moreover, their pronounced charge transfer character and large hyperpolarizability motivate investigation of their two photon absorption properties. Given that two photon excitation provides deeper tissue penetration and reduced optical scattering compared to conventional one photon excitation, these molecules are employed for two photon excited confocal cell imaging.

### Materials design and synthesis

Emitters are designed considering the following points: (a) a twisted donor–acceptor (D–A) framework for the minimal overlap of the HOMO and LUMO, (b) incorporation of heteroatoms can increase the rate of ISC as well as RISC by mixing the π, π* and the n, π* states, and (c) insertion of *tert*-butyl groups can increase steric hindrance, suppress π–π stacking, and increase solubility. This strategy will suppress energy dissipation through thermal pathways and favour radiative decay processes in the aggregated/condensed state. After careful research, a rigid acceptor core is designed by fusing dicyanobenzene (phthalonitrile and terephthalonitrile) to a *tert*-butyl substituted dibenzo-1,4-dioxine core and carbazole (Cz) as a donor due to its high HOMO energy (Scheme 1). The fusion of phthalonitrile with a modified dibenzo-1,4-dioxine core generates two regio-isomeric acceptors (Scheme S2). Subsequent coupling with carbazole leads to the formation of two final emitters: *o*CN1 and *o*CN2. Meanwhile, terephthalonitrile, modified dibenzo-1,4-dioxine and carbazole donor are fused to give *p*CN (Scheme S1). Acceptor cores were synthesized by aromatic nucleophilic substitution of tetrafluorodicyanobenzene with di-*tert*-butlycatechol in the presence of a base (Schemes S1 and S2). Another aromatic nucleophilic substitution was done with the acceptor core and the carbazole to get the final emitters (Schemes S1 and S2). All the compounds were purified by the column chromatography technique (40% DCM/hexane mixture for *p*CN and a 10% ethyl acetate/hexane mixture for *o*CN1 and *o*CN2) with a reaction yield of 13–25% and characterized by ^1^H and ^13^C NMR spectroscopy, HRMS, MALDI-TOF, and the single crystal X-ray diffraction (SCXRD) technique (see the SI).

## Results and discussion

### TADF in the solution state

**Fig. 1:**
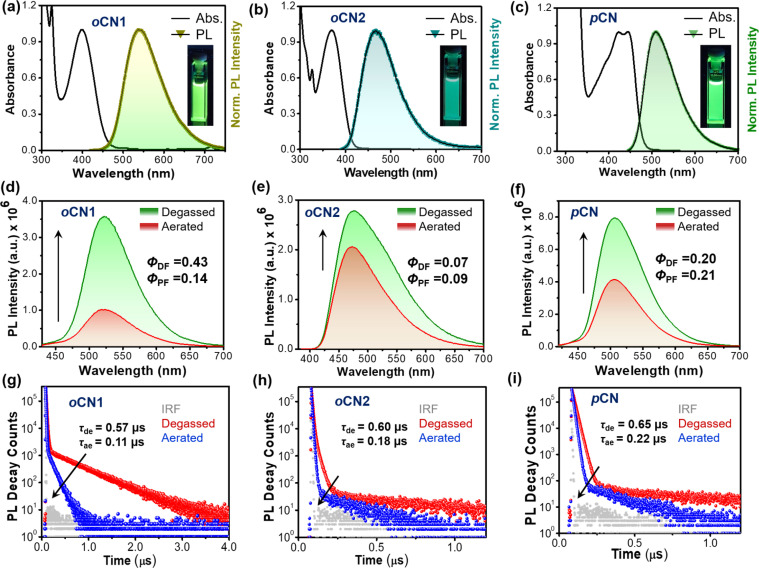
Absorption and emission spectra (shaded portion) of (a) *o*CN1, (b) *o*CN2, and (c) *p*CN, respectively, in 30 µM toluene. Inset: Cuvette pictures collected under exposure to 365 nm UV light. Aerated (O_2_) and degassed (N_2_) spectra of (d) *o*CN1, (e) *o*CN2, and (f) *p*CN, respectively. Time-resolved PL decay under degassed (red) and aerated (blue) conditions of (g) *o*CN1, (h) *o*CN2, and (i) *p*CN, respectively.

In toluene solution, all three emitters exhibit one structured high energy band below 350 nm followed by low energy bands at 400, 370 and 430 nm for *o*CN1, *o*CN2 and *p*CN respectively (Fig. 1a–c and S1). The high energy band arises from the π, π* transition, while the low energy band is attributed to charge transfer (CT) from the coupling between the carbazole donor and the acceptor (modified dioxin-benzonitrile core). Among these, the *para* (*p*CN) isomer absorbs at a longer wavelength (430 nm) compared to *ortho* (*o*CN1 and *o*CN2) isomers and shows a smaller bandgap which is calculated from DFT using the PBE0/6-31G (d,p) level of theory (Fig. S2). For all the emitters, the LUMO is mainly localized on the acceptor (dioxin-benzonitrile) core and HOMO is on the carbazole donors (Fig. S2). Notably, *o*CN1 exhibits slightly larger donor–acceptor torsional angles than the other two isomers (Fig. S3), which increases the spatial separation between the HOMO and LUMO and enhances the charge-transfer character of its S_1_ state. This is consistent with its strongest solvatochromic response and largest excited-state dipole moment among the three emitters, as discussed in the following section (Fig. S1).

All three emitters show broad, structureless emission bands with maxima in the 480–535 nm range in toluene, indicative of charge-transfer-type S_1_ emission (Fig. 1a–c). Simply changing the donor position (*ortho vs. para*) allows the emission in toluene to be tuned from cyan-blue to green, and the observed red shift of the emission maxima with increasing solvent polarity in solvatochromic measurements confirms the CT nature of these excited states. The magnitude of the red shift and the associated Stokes shift are most pronounced for *o*CN1, which exhibits a shift of ∼125 nm with respect to emission position upon changing the solvent from hexane to DMF. This significant shift strongly supports the prominent CT character of *o*CN1 in the S_1_ state. In contrast, *o*CN2 and *p*CN exhibit modest solvatochromic shifts of ∼100 and ∼80 nm, respectively. Lippert–Mataga analysis quantitatively supports this trend: the difference between ground- and excited-state dipole moments (Δ*µ*) is largest for *o*CN1 (18 D) and smaller for *o*CN2 (16.5 D) and *p*CN (14.85 D, Fig. S4). The larger Δ*µ* of *o*CN1 correlates directly with its larger solvatochromic and Stokes shifts, reflecting the strongest stabilization of a highly polar S_1_ CT state, whereas the smaller Δ*µ* values for *o*CN2 and *p*CN are consistent with their more modest red shifts and weaker CT character. Time-resolved photoluminescence (TRPL) measurements on the nanosecond time scale were performed in solvents of varying polarity to gain further insight into the nature of the S_1_ states of our designed luminogens. For *o*CN1, the emission lifetime decreases from 18 ns in toluene to 5.1 ns in THF (Fig. S5), indicating enhanced charge separation and an increased nonradiative decay rate in polar media. By contrast, *o*CN2 and *p*CN display a smaller variation in lifetime across different solvents than *o*CN1, consistent with their weaker CT character (Table S1).

The TADF behaviour of the emitters was examined in degassed toluene, where steady-state emission, time-gated spectra, and temperature-dependent studies revealed the presence of delayed fluorescence. The complete overlap of steady-state and time-gated (50 µs delay) spectra of all three compounds confirms that the delayed fluorescence originates from the lowest singlet excited state (S_1_) (Fig. S6). Among the three, *o*CN1 demonstrates the largest contribution of delayed fluorescence (*Φ*_DF_ = 43%) and highest PLQY (*Φ*_PL_ = 57%). It also exhibits the fastest reverse intersystem crossing rate (*k*_RISC_ = 6.2 × 10^6^ s^−1^), which can be attributed to its smallest singlet–triplet energy gap of 0.06 eV (Table 1). Experimentally determined Δ*E*_ST_ values from temperature-dependent analyses (Fig. S7) follow the sequence *o*CN1 < *o*CN2 < *p*CN, consistent with the TD-DFT calculations (Fig. S2 and Table 1). Transient PL decay profiles recorded under nitrogen-purged conditions reveal sub-microsecond delayed lifetimes (0.57, 0.60, and 0.65 µs for *o*CN1, *o*CN2, and *p*CN, respectively). Upon exposure to oxygen, the emission is significantly quenched, supporting the involvement of triplet states in the emissive process. The markedly enhanced RISC rate for *o*CN1 arises from the effective coupling between a CT-dominant S_1_ state and a locally excited (LE)-type T_1_ state (Fig. S8). This interaction leads to spin–orbit coupling (SOC ≈ 0.8 cm^−1^), in accordance with the El-Sayed rule.^35^ Natural transition orbital (NTO) analyses reveal spatially well-separated hole and electron distributions in the S_1_ state of *o*CN1 (Fig. S8), corroborating the solvatochromic behaviour and dominant CT character observed experimentally. Collectively, the minimal Δ*E*_ST_, significant SOC, and rapid RISC rate indicate *o*CN1 as the most efficient TADF-active emitter among the three isomers, highlighting its potential as a high-performance material for solution-processable optoelectronic devices.

**Table 1:** Photophysical parameters in toluene solution

Emitters	*Φ*_PL_	*τ*_p_/*τ*_d_ [ns]/[ns]		*Φ*_DF_/*Φ*_PF_	a *k*_r_ [10^7^ s^−1^] (S_1_ → S_0_)	b *k*_nr/IC_ [10^7^ s^−1^] (S_1_ → S_0_)	c *k*_ISC_ [10^7^ s^−1^] (S_1_ → T_1_)	d *k*_RISC_ [10^6^ s^−1^] (T_1_ → S_1_)	eΔ*E*_ST_ (in eV)
		N_2_	O_2_						
*o*CN1	0.57	18/570	7.5/112	0.43/0.14	0.8	0.60	4.8	6.2	0.06
*o*CN2	0.15	6.5/600	4.4/182	0.07/0.09	1.38	7.8	14	1.4	0.11
*p*CN	0.41	12/650	9.2/228	0.20/0.21	1.75	2.5	6.5	1.8	0.14

a *k*_r_ (S_1_ → S_0_) = *Φ*_PF_/*τ*_p_.

b *k*_nr/IC_ (S_1_ → S_0_) = (*k*_r_/*Φ*_PL_) − *k*_r_.

c *k*_ISC_ (S_1_ → T_1_) = (1 − *Φ*_PF_)/*τ*_p_.

d *k*_RISC_ (T_1_ → S_1_) = *Φ*_DF_/(*k*_ISC_ × *τ*_p_ × *τ*_d_ × *Φ*_PF_).

e Experimentally determined energies from the onset of fluorescence and phosphorescence spectra. Prompt fluorescence (PF) and delayed fluorescence (DF) contributions are measured in toluene solvent (30 µm) under aerated (O_2_) and degassed (N_2_) conditions. Upon N_2_ purging, the total quantum yield (*Φ*_PL_) can be measured from the steady state spectra. Subsequently, O_2_ purging in the same solution enables determination of prompt fluorescence contribution (*Φ*_PF_) from the steady state spectra. Then, *Φ*_DF_ is measured using *Φ*_DF_ = *Φ*_PL_ − *Φ*_PF_.

### TADF in aggregates

**Fig. 2:**
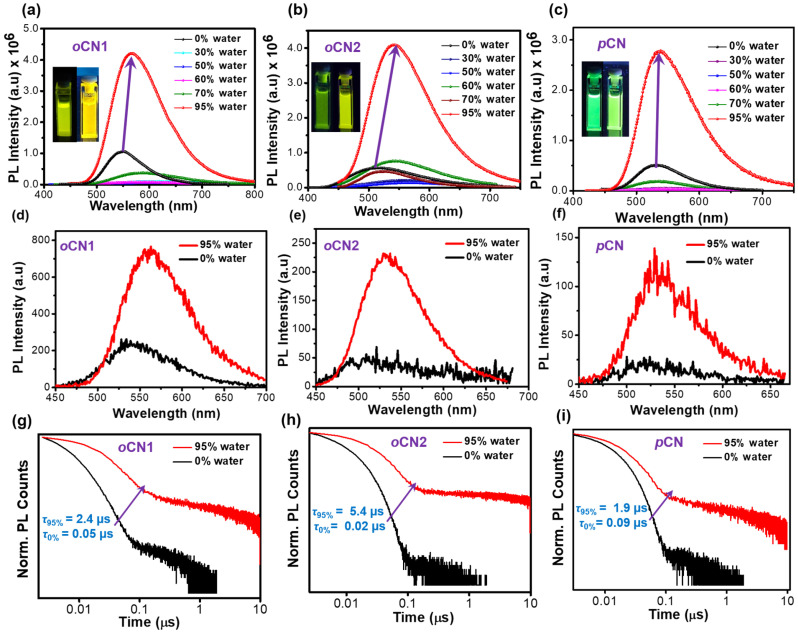
Variation of steady state, gated PL intensity (50 µs delay, 0.2 ms window) and delayed fluorescence lifetime with 0% and 95% water content for (a, d and g) *o*CN1; (b, e and h) *o*CN2 and (c, f and i) *p*CN. Inset: Cuvette pictures of monomers (left, 0% water) and aggregates (right, 95% water) under exposure to 365 nm UV light.

In THF solution (0% water), all the emitters display weak photoluminescence properties, which are reflected in their poor PLQYs of around 7–19% (Fig. 2). With the addition of water, the emission gradually red shifts and its intensity decreases, which can be ascribed to an enhanced charge transfer character in the highly polar environment. When the water fraction exceeds about 60%, the PL intensity starts to increase again as the aggregates are formed, and at 95% water the emission is enhanced by 6-fold for *o*CN1, 8-fold for *o*CN2, and 10-fold for *p*CN, suggesting pronounced AIE behaviour of luminogens. In this aggregated state, restriction of intramolecular motions and vibrations suppresses nonradiative decay channels,^20^ which in turn leads to a strong increase in the photoluminescence quantum yields. Furthermore, the formation of nanoaggregates at higher water content for all these AIEgens was confirmed by dynamic light scattering (DLS) studies (Fig. S10).

Notably, the molecular aggregates formed at 95% water content exhibit PL decay time in the microsecond range, with an average lifetime of 2.4 µs, 5.4 µs and 1.9 µs for *o*CN1, *o*CN2, and *p*CN, respectively (Fig. 2g–i and Table 2), which is significantly higher than that observed in pure THF/toluene. Here, it is important to note that this long lifetime component was not prominent at low water content (<60%), when the aggregates are not properly formed. Notably, these microsecond lifetimes are typical signatures of triplet harvesting processes and, therefore, point to the activation of the TADF process in the aggregated state. Time gated emission measurements (50 µs delay, room temperature) further confirm this conjecture, revealing much stronger emission from the aggregates and thus a more efficient delayed fluorescence channel (Fig. 2d–f). In the aggregated state (95% water), all three luminogens show a pronounced increase in both prompt fluorescence efficiency (*Φ*_PF_) and delayed fluorescence quantum yield (*Φ*_DF_), pointing to highly efficient exciton utilization. The *Φ*_DF_ values increase from nearly zero in THF to about 29%, 25%, and 18% in aggregates for *o*CN1, *o*CN2, and *p*CN, respectively, and the overall PLQY (*Φ*_PL_) reaches 63%, 40%, and 52%, respectively (Fig. S11 and S12 and Table 2). This behaviour is characteristic of AIDF, where both prompt fluorescence from singlets and delayed fluorescence from triplet-assisted singlets are operative in the aggregated state. To understand the origin of this AIDF response, we compared the excited state deactivation channels in the molecular and aggregated states. In polar solvents such as THF, the excited S_1_ and T_1_ states are stabilized and relax *via* non-radiative pathways (internal conversion, vibrational and rotational motions, *etc.*), leading to very short lifetimes that suppress both ISC and RISC. Upon aggregation, the local environment around the emitters becomes effectively hydrophobic, which reduces excessive stabilization of the CT states and at the same time restricts intramolecular motions that drive the internal conversion pathway. Consequently, all three regioisomers display modulation of the nonradiative internal conversion rate (*k*_IC_) and reverse intersystem crossing rate (*k*_RISC_). The internal conversion rate (*k*_IC_) decreases markedly from 16.5 × 10^7^ to 1.05 × 10^7^ s^−1^ from monomers to aggregates for *o*CN1, with 20- fold and 4-fold reductions observed for *o*CN2 and *p*CN, respectively (Table 2). This suppression of internal conversion facilitates more efficient ISC and RISC, which in turn increases the delayed fluorescence channel.

**Table 2:** All photophysical parameters in monomers, aggregates and neat films

Emitters	Systems	*Φ*_PL_	*τ*_p_ [ns]/*τ*_d_ [µs]	*Φ*_DF_/*Φ*_PF_	a *k*_r_ [10^7^ s^−1^] (S_1_ → S_0_)	b *k*_IC/nr_ [10^7^ s^−1^] (S_1_ → S_0_)	c *k*_ISC_ [10^7^ s^−1^] (S_1_ → T_1_)	d *k*_RISC_ [10^5^ s^−1^] (T_1_ → S_1_)
*o*CN1	eMonomers	0.14	5.1/—	—/0.14	2.7	16.5	—	—
	eAggregates	0.63	18/2.4	0.29/0.34	1.8	1.05	3.6	5.4
	fNeat film	0.78	12/20	0.15/0.63	5.2	1.46	3.1	0.32
*o*CN2	eMonomers	0.07	4.5/—	—/0.07	1.5	20	—	—
	eAggregates	0.40	20/5.4	0.25/0.15	0.75	1.12	4.2	3.6
	fNeat film	0.31	16/38	0.12/0.19	1.2	2.67	5.1	0.2
*p*CN	eMonomers	0.19	9.6/—	—/0.19	1.9	8.1	—	—
	eAggregates	0.52	14/1.9	0.18/0.34	2.4	2.2	4.7	4.2
	fNeat film	0.54	7.9/18	0.07/0.47	5.9	5.02	6.7	0.15

a *k*_r_ (S_1_ → S_0_) = *Φ*_PF_/*τ*_p_.

b *k*_IC/nr_ (S_1_ → S_0_) = (*k*_r_/*Φ*_PL_) − *k*_r_.

c *k*_ISC_ (S_1_ → T_1_) = (1 − *Φ*_PF_)/*τ*_p_.

d *k*_RISC_ (T_1_ → S_1_) = *Φ*_DF_/(*k*_ISC_ × *τ*_p_ ×*τ*_d_ ×*Φ*_PF_).

e Measured using 30 µm sample concentration in THF solvent at 0% and 95% water content.

f Measured in neat films.

**Fig. 3:**
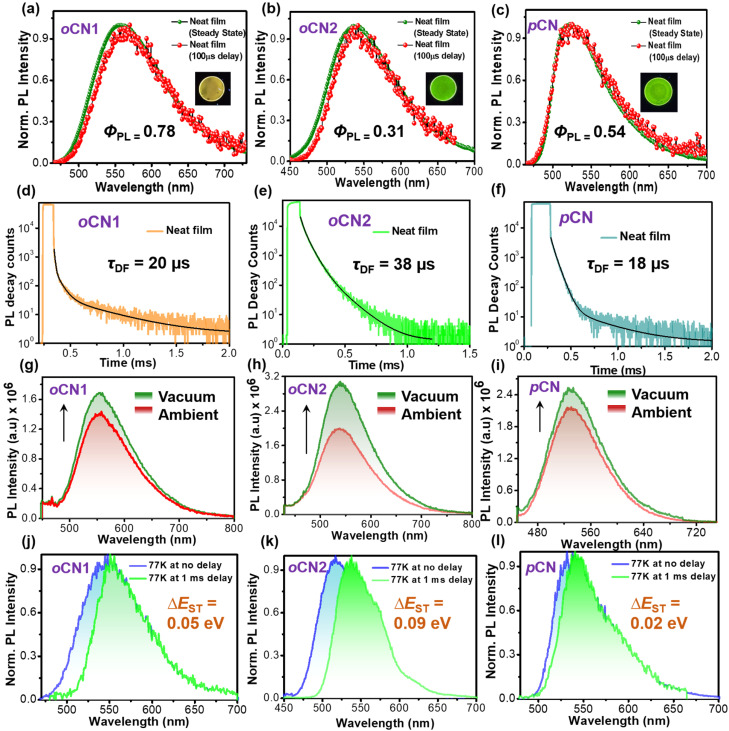
Steady-state and time-gated (100 µs delay, 5 ms sample window) RT emission spectra in neat films of (a) *o*CN1, (b) *o*CN2, and (c) *p*CN (inset shows the color of the film under exposure to 365 nm UV light). Delayed fluorescence lifetime for (d) *o*CN1, (e) *o*CN2, and (f) *p*CN. PL intensity in a vacuum and under ambient conditions for (g) *o*CN1, (h) *o*CN2, and (i) *p*CN. Gated emission spectra at 77 K with no delay and 1 ms delay and a 10 ms window for (j) *o*CN1, (k) *o*CN2, and (l) *p*CN.

**Fig. 4:**
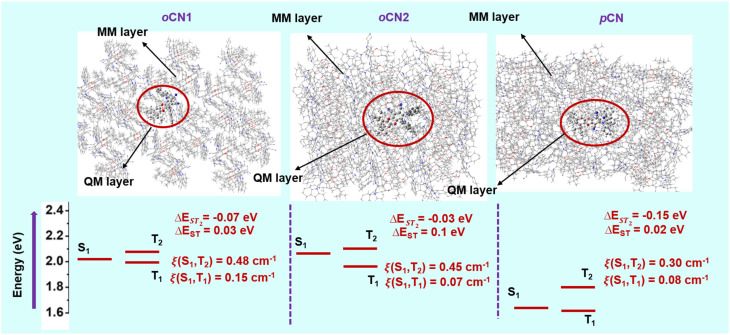
TD-DFT and SOC calculation in the aggregated state using the QM/MM model. QM and MM layers were chosen from the crystal structure for QM/MM simulations for *o*CN1, *o*CN2 and *p*CN. For the QM/MM calculation, the QM layer has been treated with the PBE0/6-31G (d, p) level of theory, while the MM layer has been treated with a classical UFF model of theory.

**Fig. 5:**
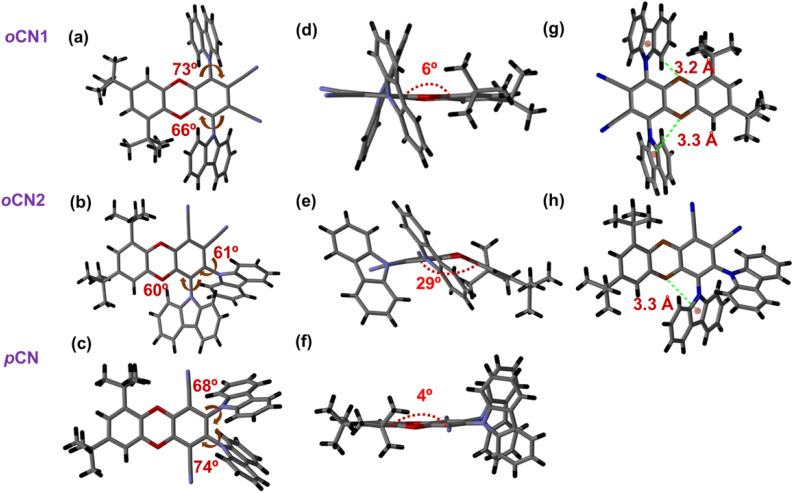
Crystal structure of (a) *o*CN1, (b) *o*CN2, and (c) *p*CN along the crystallographic *b* axis. Puckered angles between the two peripheral benzene rings in (d) *o*CN1, (e) *o*CN2, and (f) *p*CN, respectively. Interaction distance between the lone pair of the dioxin core and Cz ring in (g) *o*CN1 and (h) *o*CN2.

To assess the persistence of the AIDF effect and its applicability in non-doped OLED devices, the photophysical properties were investigated in neat films. All three emitters display bright green to yellow emission with PL maxima between 525 and 565 nm (Fig. 3). Spectral position and shape of the steady state and time gated spectra match, demonstrating the presence of TADF as the major emissive pathway, with both contributions arising from the same singlet state (Fig. 3a–c and S13). To quantify their solid-state emissive performance, the absolute PLQY was measured. *o*CN1 exhibits a value of 78%, notably higher than that of *o*CN2 (31%) and *p*CN (54%). *o*CN2 adopts a puckered conformation (evident from SC-XRD analysis, Fig. 5e) that enables intermolecular π–π stacking, leading to reduced PLQY. The transient PL decays at room temperature display an average delayed component of 20 µs, 38 µs, and 18 µs for *o*CN1, *o*CN2, and *p*CN, respectively, along with prompt fluorescence in the nanosecond regime. The observation of delayed emission in the solid state further confirms that a spin-flip process is active in the emissive mechanism (Fig. 3d–f). Temperature dependent transient PL decay measurements of the fluorescence peak reveal an initial sharp drop in the delayed fluorescence lifetime on cooling from room temperature to 77 K, reflecting insufficient thermal energy to upconvert the triplets to the singlet state (Fig. S14). At longer time scales, the lifetime increases again due to emerging phosphorescence contribution, and the observed long-lived components of 206 ms, 370 ms, and 61 ms for *o*CN1, *o*CN2, and *p*CN, respectively, are assigned to phosphorescence from the T_1_ state (Fig. S15). At 77 K with a 1 ms delay, the gated spectra show a new peak appears at longer wavelengths, consistent with phosphorescence originating from the lowest triplet (T_1_) state (Fig. 3j–l). From the spectral onsets, the S_1_–T_1_ gaps (Δ*E*_ST_) are estimated to be 0.05 eV, 0.09 eV, and 0.02 eV for *o*CN1, *o*CN2, and *p*CN, respectively (Fig. 3j–l), and the corresponding calculated values of 0.03 eV, 0.1 eV, and 0.02 eV (Fig. 4) obtained from computational studies agree well with the experiment. These small Δ*E*_ST_ values, which result from the spatial separation of donor and acceptor units, reduce the RISC barrier and thereby promote efficient TADF in the neat films. RISC plays a crucial role in TADF, because it upconverts non-emissive triplet excitons back to the singlet state, thereby boosting the overall emission efficiency. Thus, RISC rate constants were determined from the fractional quantum yields of prompt (*Φ*_PF_) and delayed (*Φ*_DF_) fluorescence, obtained by integrating the steady state PL under ambient and vacuum conditions (Fig. 3g–i). *o*CN2 exhibits the highest delayed contribution (*Φ*_DF_/*Φ*_PF_ = 0.63), followed by *o*CN1 (*Φ*_DF_/*Φ*_PF_ = 0.24) and *p*CN (*Φ*_DF_/*Φ*_PF_ = 0.15). Among the three emitters, *o*CN1 exhibits the highest RISC rate both in aggregates (5.4 × 10^5^ s^−1^) and in neat films (0.32 × 10^5^ s^−1^) (Table 2). To gain deeper insight into this enhancement, QM/MM calculations were performed using the two-level ONIOM model with crystal geometry as input.^36^ From the energy alignment of the excited states, it has been observed that the S_1_–T_1_ gap (Δ*E*_ST_) increases from 0.03 eV for *o*CN1 to 0.1 eV for *o*CN2, while for *p*CN it is 0.02 eV (Fig. 4). Along with this, the T_2_ state is located in close proximity to S_1_, and the corresponding energy gaps (Δ*E*_ST2_) become −0.07 eV for *o*CN1 to −0.03 eV for *o*CN2, and −0.15 eV for *p*CN. The T_1_–T_2_ separations are 0.1 eV, 0.13 eV, and 0.17 eV for *o*CN1, *o*CN2, and *p*CN, respectively. The spin–orbit coupling (SOC) matrix elements between S_1_ (predominantly CT) and T_1_ (predominantly CT) are 0.15, 0.07, and 0.08 cm^−1^ for *o*CN1, *o*CN2, and *p*CN, whereas the SOC values between S_1_ (predominantly CT) and T_2_ (HLCT for *o*CN1 and *o*CN2; predominantly CT for *p*CN) are significantly larger for *o*CN1 and *o*CN2 with values of 0.48 and 0.45 and a little bit smaller for *p*CN with a value of 0.30 cm^−1^ (Fig. 4 and S16 and Table S2).

In the spin-vibronic model of TADF, efficient RISC is obtained when the singlet and triplet states are separated by small energy gaps, the spin–orbit coupling is sufficiently strong, and vibronic coupling connects an intermediate triplet state lying close in energy to S_1_.^37,38^ Notably, when the energy separation between two triplet states (T_1_ and T_2_) is small and both lie close in energy to S_1_, vibronic coupling can strongly mix the triplet manifold and open an extra pathway for RISC *via* T_2_, thereby enhancing the overall RISC rate.^39^ Conclusively, in *o*CN1 aggregates, the smallest T_1_–T_2_ gap (0.1 eV) and the largest SOC (0.48 cm^−1^) between S_1_ and T_2_ provide favourable conditions for such a spin-vibronic RISC channel, rationalizing its high experimental RISC rate. By contrast, *o*CN2 and *p*CN display larger T_1_–T_2_ separations (0.13 and 0.17 eV) and/or smaller S_1_–T_2_ SOC, along with a slightly larger S_1_–T_1_ gap in the case of *o*CN2, which collectively reduce the efficiency of the RISC process compared to *o*CN1.

### Single crystal X-ray diffraction (SC-XRD) and structure–packing–property co-relations

In order to understand the role of intermolecular interactions and molecular packing induced delayed fluorescence, single crystals of *o*CN1, *o*CN2, and *p*CN were grown from slow evaporation of hexane/THF, hexane/methanol and ethyl acetate solutions, respectively. The resulting crystals of *o*CN1 adopt a needle like morphology and crystallize in the monoclinic *P*2_1_/*n* space group, whereas *o*CN2 and *p*CN form block shaped crystals with triclinic *P*1̄ and orthorhombic *Pbca* space groups, respectively (crystallographic parameters are summarized in Tables S3–S5). SCXRD reveals donor–acceptor dihedral angles of 66–73° (*o*CN1), 60–61° (*o*CN2) and 68–74° (*p*CN), consistent with TDDFT optimized structures (Fig. 5a–c). This result is in close agreement with our theoretical predictions, thereby validating that the chosen level of theory reliably reproduces the experimental observations (Fig. S3).

Among the three emitters, the acceptor core of *o*CN2 adopts a prominent bent geometry with an angle of 29°, whereas the acceptor cores of *o*CN1 and *p*CN adopt almost linear geometry with angles of 6° and 4° (Fig. 5d–f and S19). In *o*CN2, the lone pair of the oxygen atom of DBO interacts with the carbazole moiety at a distance of less than 3.4 Å *via* dispersion forces (Fig. 5h). This specific interaction is known to stabilize puckered conformation,^40,41^ but a flat structure would otherwise increase the interatomic distance and weaken these stabilizing forces. Consequently, this dispersion-driven attraction is the primary reason the acceptor core of *o*CN2 adopts a puckered rather than planar geometry. This puckered geometry brings neighbouring molecules into close proximity and gives rise to face-to-face π–π stacking between the aromatic cores with an interplanar distance of 3.4 Å (Fig. S20); this interaction facilitates nonradiative decay channels and is consistent with the reduced PLQY of 31% observed in neat films (Table 2). In contrast, for *o*CN1 and *p*CN, the donors are placed in less sterically congested environments and retain larger donor–acceptor dihedral angles, so they can adopt more twisted geometry (donor–acceptor dihedral angles of 66°–73° for *o*CN1 and 68°–74° for *p*CN), while keeping the acceptor core closer to planar (Fig. 5); this reduces the driving force for ring puckering and avoids the strong face to face π–π stacking seen for *o*CN2 (Fig. S20 and S21). This reduced tendency for face-to-face aggregation is consistent with their higher solid state PLQYs (78% and 54% for *o*CN1 and *p*CN respectively in neat films) relative to *o*CN2, highlighting that even small structural changes can strongly alter packing and emission behaviour. Due to the puckered conformation in *o*CN2, conjugation is disrupted at the dioxin core and the terminal benzene ring does not function as an extended part of the acceptor; in contrast, it remains weakly conjugated in *o*CN1 and *p*CN, which is evident from spatial distribution of the excited state wavefunction obtained from theoretical calculation (Fig. S16). Thus, the different emission colours and photophysical properties of the three regio-isomers originate from their distinct molecular geometries and crystal packing. Time resolved measurements on the single crystals further underscore the impact of packing. Notably, crystalline samples display significantly prolonged average lifetimes of 14 µs, 62 µs, and 20 µs for *o*CN1, *o*CN2, and *p*CN, respectively, in the green to orange region, following the same trend observed in neat films (Fig. S22 and S23). In particular, *o*CN2 shows a striking lifetime enhancement from submicrosecond delayed fluorescence in THF solution to 62 µs in the crystalline state, which can be ascribed to the strong face to face π–π stacking that promotes efficient intermolecular electronic coupling and stabilizes triplet derived excited states,^42,43^ and thereby markedly prolonged TADF lifetime. Together, these structure–packing–property correlations emphasize that fine control over both molecular geometry and solid-state packing is crucial for optimizing the TADF performance of this AIDF class of emitters.

**Fig. 6:**
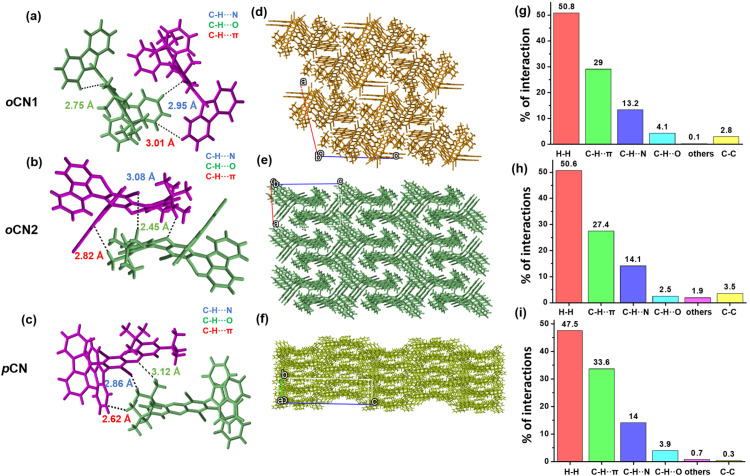
(a–c) Intermolecular non-covalent interactions of *o*CN1, *o*CN2, and *p*CN in dimer form. Different color codes represent two different molecules as well as different types of interactions. (d–f) Supramolecular interaction induced crystal structure packing; herringbone, lamellar and wave-like bulk crystal structure packing along the *a*- to *c*-axis in *o*CN1, *o*CN2, and *p*CN respectively. (g–i) Comparison of different types of non-covalent interactions in tabular form calculated from Hirshfeld surface analysis.

To gain deeper insight into the nature and relative strength of the intermolecular contacts, we further analysed the non-covalent interactions present in all three crystals. All three emitters exhibit fundamentally different molecular packing modes from each other. *o*CN1 adopts a herringbone packing motif with an antiparallel slip stacked arrangement (Fig. 6d and S24). This packing mode is stabilized by a diverse set of non-covalent directional contacts, including C–H⋯N, C–H⋯π and C–H⋯O interactions with distances of 2.75–3.01 Å. *o*CN2 adopts slipped cofacial π-stacking of the conjugated cores arranged into lamellar layers. In contrast to *o*CN1 and *o*CN2, *p*CN does not form extended cofacial π-stacks or a classical herringbone lattice. Instead, SCXRD shows that *p*CN molecules assemble into polar head-to-tail donor–acceptor chains, yielding a non-centrosymmetric arrangement of molecular dipoles with negligible intermolecular π-overlap. These packing arrangements result in several non-covalent intermolecular interactions dominated by C–H⋯π, C–H⋯N, C–H⋯O and π⋯π contacts (2.62–3.12 Å for *p*CN). To gain deeper insight into the relative contributions of these contacts, we performed Hirshfeld surface analysis (Fig. 6g–i and S25). This analysis reveals that, although H⋯H van der Waals interactions constitute the largest fraction of surface contacts, their weak enthalpic contribution (∼0.4–4 kJ mol^−1^)^44^ makes them less important for stabilizing the packing than stronger, more directional interactions.

Hirshfeld surface analysis further quantifies the π–π interactions as 3.5% for *o*CN2, 2.8% for *o*CN1 and 0.3% for *p*CN, consistent with the higher solid state PLQYs of *o*CN1 and *p*CN relative to *o*CN2. All three molecules exhibit dominant C–H⋯π and C–H⋯N interactions, which possess relatively high stabilization energies (∼10.3 kJ mol^−1^ and 4–20 kJ mol^−1^ respectively)^45,46^ and act as key forces stabilizing their crystal lattices. In addition, C–H⋯O interactions, although accounting for only 1.5–3.9% of the total contacts, display comparatively high stabilization energies (∼41–104 kJ mol^−1^);^47^ thus, they also make an important contribution to the packing. Taken together, these intermolecular contacts provide molecular rigidity and reduce internal motions, thereby suppressing nonradiative energy loss pathways and promoting stronger, more efficient solid-state emission. This behavior is consistent with that observed in THF/water aggregates, where the internal conversion rate (*k*_IC_) decreases by up to approximately 20-fold.

Overall, single crystal X-ray diffraction provides a clear link between the molecular and packing structures of the three regioisomers and their distinct solid state photophysical properties. *o*CN1 combines a herringbone slip stacked packing with a total of 2.8% π–π interactions and twisted donor–acceptor geometry yielding the smallest Δ*E*_ST_, the fastest delayed component and the highest PLQY (more denser rigid environment suppressing the non-radiative decay channel); *o*CN2's puckered, π stacked lattice gives a larger Δ*E*_ST_, reduced PLQY but the longest delayed emission; and *p*CN falls between these limits, with correspondingly intermediate solid-state emission and TADF characteristics. The fact that the same trends appear in both crystals and neat films demonstrates that the regioisomerism-controlled geometry and packing captured by SCXRD translate directly into TADF efficiency, PLQY and the emission colour observed in solid-state films and c-LEDs.

## Applications

### Converted LEDs

**Fig. 7:**
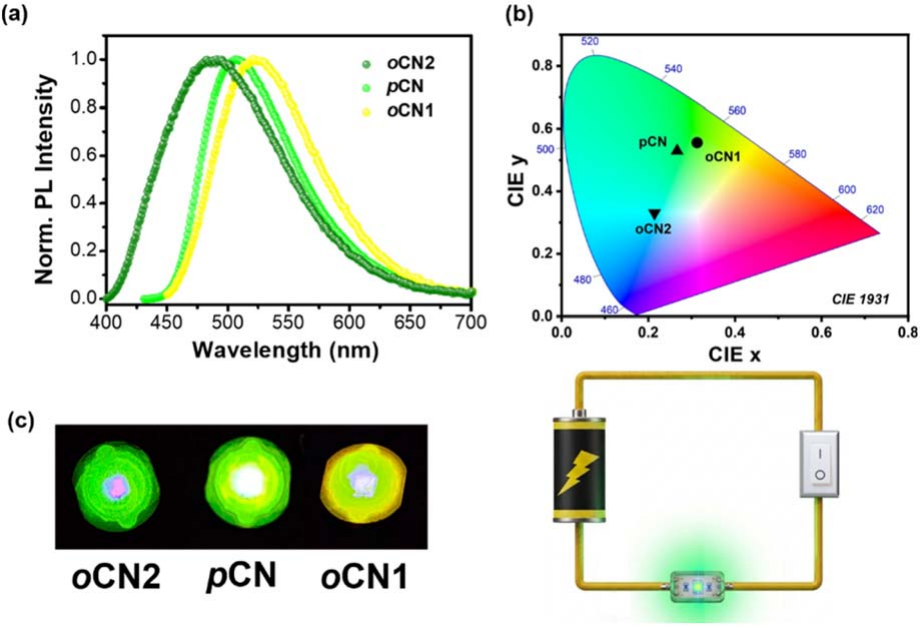
(a) Emission spectra of all three fabricated c-LEDs, (b) CIE-chromaticity plots (CIE 1931) of the emission spectra obtained in all three isomers, (c) emission glow from all three c-LEDs in three different color regimes and the schematic of the c-LED devices fabricated.

Inspired by the three unique emission characteristics exhibited by the isomers, we directed our efforts toward the development of converted LEDs (c-LEDs). Here PMMA was chosen as a host matrix because its high triplet energy prevents back energy transfer while its rigid environment also suppresses nonradiative decay, enhancing emitter efficiency.^48^ The c-LEDs were constructed by coating InGaN chips with a PMMA layer doped with 10 wt% emitter, with an elaborate fabrication procedure described in the SI (Section S8 and Fig. S26). Under a 3 V bias, *o*CN1 emits yellow color with CIE coordinates of (0.31, 0.56), while *o*CN2 and *p*CN exhibit green and greenish yellow emission with CIE coordinates of (0.21, 0.33) and (0.27, 0.53) respectively (Fig. 7a–c). It is important to highlight that the observed emission colors closely resemble the photoluminescent (PL) emissions recorded from the 10 wt% doped PMMA films (Fig. S17). This study demonstrates the viability of employing the three emitters as promising candidates for future OLED development.

### Two-photon absorption and cell imaging

**Fig. 8:**
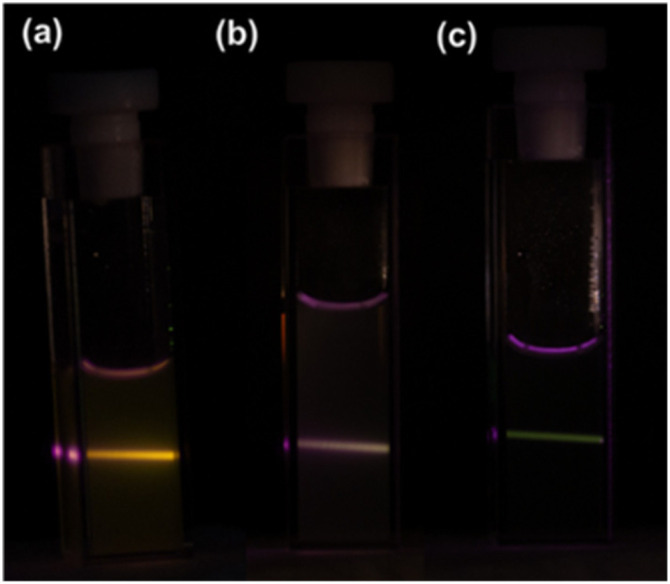
A laser beam of 800 nm light focused on a solution of (a) *o*CN1 in 95% water/THF (30 µM), (b) *o*CN2 in 95% water/THF (30 µM), and (c) *p*CN in 95% water/THF (30 µM). Clear yellow and green fluorescence was observed in the aggregates for *o*CN1, *o*CN2 and *p*CN respectively.

**Fig. 9:**
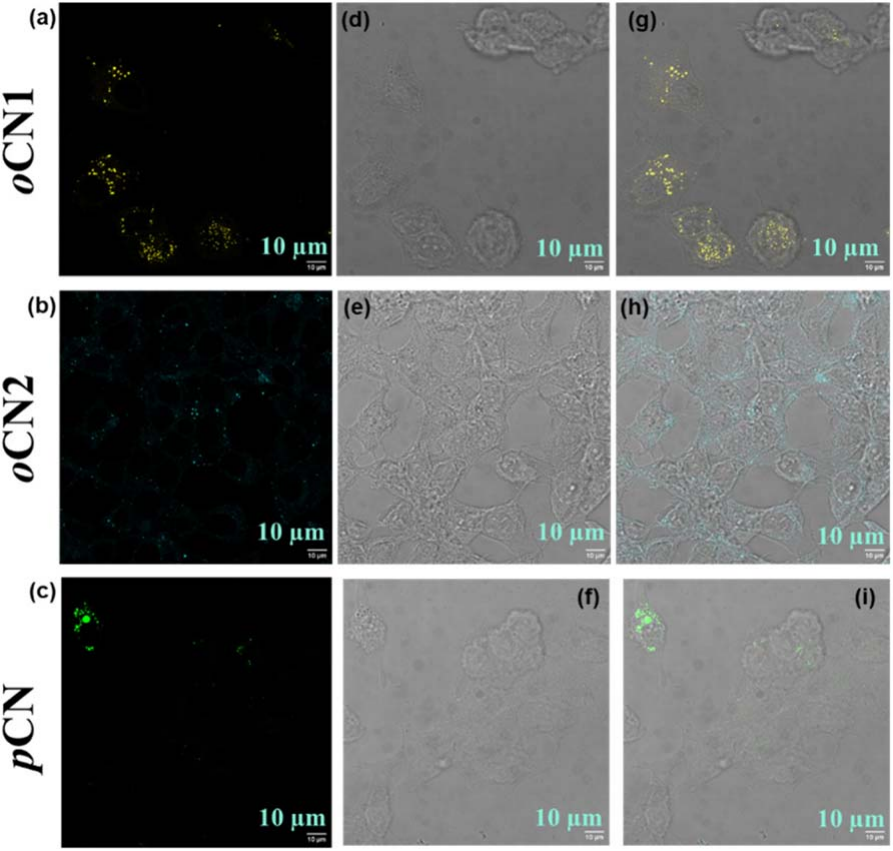
Two-photon microscopy images (excitation wavelength = 800 nm, and the collection window is from 570 nm to 620 nm for *o*CN1, 490 nm to 540 nm for *o*CN2, and 520 nm to 570 nm for *p*CN) of HeLa cells: (a–c) dye treated two-photon imaging, (d–f) differential interference contrast (DIC) images, and (g–i) DIC image merged with dye treated two-photon images.

Two-photon fluorescence bioimaging offers distinct advantages over one-photon excitation. By employing near-infrared (NIR) photons, it minimizes photodamage to live samples and reduces light scattering, enabling deep tissue penetration.^49,50^ Due to extensive π-conjugated structures and strong charge transfer properties, all our AIDF molecules exhibit two-photon activity in the aggregated state (Fig. 8 and 9). The two-photon excitation–emission spectra (*λ*_ex_ = 800 nm) of aggregates closely resemble their one-photon spectra (*λ*_ex_ = 400 nm), indicating that identical emissive states are involved (Fig. S27 and S28). Furthermore, the anti-Stokes-shifted emission observed under 690–1100 nm excitation (Fig. S27d–f), together with a log–log intensity slope greater than 1.5, confirms genuine two-photon-excited emission with negligible first-order (one-photon) contribution (Fig. S29).

Additionally, the biocompatibility of the luminates was evaluated using the MTT cell viability assay in MCF7 cells. The luminates did not show cytotoxicity to the cells at 5, 10 and 15 µM concentrations (Fig. S31), thereby demonstrating that the luminogens are well tolerated by mammalian cells at the tested levels. Two-photon confocal microscopy on HeLa cells reveals bright intracellular emission (Fig. 9), with *o*CN1, *o*CN2 and *p*CN providing distinguishable emission windows (570–620 nm, 490–540 nm and 520–570 nm collection, respectively). The luminogens form stable intracellular aggregates distributed throughout the cytoplasm and retain their AIDF-character under two-photon excitation, enabling long-lived, high-contrast images with reduced photodamage due to NIR excitation. Together, these results demonstrate that the regioisomeric AIDF emitters function as robust, colour-tunable two-photon bioimaging agents for further biological applications.

Importantly, the application performance of these materials directly reflects the regioisomerism-driven photophysics. *o*CN1, with the smallest Δ*E*_ST_, highest PLQY and fastest *k*_RISC_, enables the fabrication of bright yellow c-LEDs and exhibits strong two-photon-excited emission in both aggregated states and live cells. In contrast, the puckered, π-stacked *o*CN2 shows lower PLQY and correspondingly less intense c-LED output, yet its longer TADF lifetime may be advantageous for time-gated bioimaging or lifetime-based sensing. The *para* isomer *p*CN shows an intermediate PLQY and an emission wavelength between those of the *ortho* isomers, which is useful for both imaging and device applications. Thus, our regioisomeric design principles will be useful for the development of next-generation AIDF materials for optoelectronic and bioimaging applications.

## Conclusion

We have demonstrated that integrating AIE with TADF in carbazole–dicyano–dioxin regioisomers affords efficient triplet harvesting and color tunable emission from solution to aggregates, neat films and crystals. The incorporation of oxygen atoms and attachment of *tert*-butyl groups make the molecules more rigid and help suppress aggregation caused quenching. These substituents also enforce twisted donor–acceptor geometries. Subtle changes in the carbazole connection pattern strongly regulate molecular geometry, crystal packing and excited state energetics and thereby the optical and photophysical responses of *o*CN1, *o*CN2 and *p*CN. *o*CN1 combines a twisted donor–acceptor arrangement with a nearly planar acceptor core, leading to the smallest Δ*E*_ST_, the fastest RISC and the highest solid state PLQY. In contrast, the *ortho* linked *o*CN2 adopts a puckered acceptor and strongly π–π stacked lattice, which activates nonradiative decay channels and lowers PLQY, yet stabilizes triplet derived states and markedly prolongs delayed emission in the neat film and crystals. The *para* isomer, *p*CN, exhibits intermediate packing and photophysical behaviour. The strong charge-transfer character and solid-state AIDF behaviour enable them for efficient two-photon bioimaging and use in converted LEDs. Together, our results show that regio-isomer engineering of twisted donor–acceptor dioxin frameworks is a useful design strategy for next-generation multifunctional TADF-AIE emitters for optoelectronic and bioimaging applications.

## Author contributions

A. B. and P. H. conceived and designed the project. A. B. carried out the synthesis of the molecules, designed and executed all the spectroscopic measurements, and analysed the data. M. D. contributed to the two-photon absorption, c-LED experiments and theoretical calculations. A. J. M. and M. L. designed and performed the cell viability and two-photon cell imaging experiments. M. D. A. assisted with the analysis of the crystal structures. M. D. helped analyse the theoretical results. P. H. supervised the project and provided valuable insights throughout its progression. A. B., M. D. and P. H. wrote the manuscript with input from all the authors.

## Conflicts of interest

There are no conflicts to declare.

## Supplementary Material

SC-OLF-D6SC03725A-s001

SC-OLF-D6SC03725A-s002

SC-OLF-D6SC03725A-s003

## Data Availability

CCDC 2551023, 2551024 and 2551025 contain the supplementary crystallographic data for this paper.^51*a*–*c*^ The data supporting this study are available within the supplementary information (SI) and from the authors on reasonable request. Supplementary information: instrumentation and experimental details, materials and methods, characterization, crystallographic data, and computational details. See DOI: https://doi.org/10.1039/d6sc03725a.
