# Supplementary material for: Regioisomeric carbazole–dicyano–dioxin AIDF emitters for efficient triplet harvesting, two-photon absorption and bioimaging
Source: Chem Sci. 2026 Jul 24;17(36):17297–309. doi: 10.1039/d6sc03725a (PMC13430608; doi:10.1039/d6sc03725a)
Supplement: Supplementary file 1 [file SC-017-D6SC03725A-s001.pdf]

Supplementary Information (SI) for Chemical Science.
This journal is © The Royal Society of Chemistry 2026

Electronic Supplementary Information

Regioisomeric carbazole-dicyano-dioxin AIDF emitters for efficient triplet harvesting, two-photon absorption and bioimaging

*Anwesha Bera, Madhusudan Dutta, Ajay J. Malik, Madan D. Ambhore, Mayurika Lahiri, and Partha Hazra**

Anwesha Bera, Madhusudan Dutta

Department of Chemistry, Indian Institute of Science Education and Research Pune, Dr. Homi Bhabha Road, Pune, 411008, India

Ajay J. Malik, Mayurika Lahiri

Department of Biology, Indian Institute of Science Education and Research (IISER), Pune (411008), Maharashtra, India

Madan D. Ambhore

Department of Chemistry, Yeshwant Mahavidyalaya Nanded, Nanded, PIN-431602, Maharashtra, India

Partha Hazra

Department of Chemistry and Centre for Energy Science, Indian Institute of Science Education and Research Pune, Dr. Homi Bhabha Road, Pune, 411008, India

Corresponding author E-mail: p.hazra@iiserpune.ac.in

**Section S1**	Instrumentation
**Section S2**	Materials
**Section S3**	Experimental and theoretical methods
**Section S4**	Cell culture and drug treatments
**Section S5**	Synthesis and Characterization
**Section S6**	Photophysical and theoretical studies
**Section S7**	Crystal data
**Section S8**	Fabrication of converted LEDs
**Section S9**	Two-photon absorption properties
**Section S10**	Two-photon imaging studies

Section S1: Instrumentation

All synthesized compounds were characterized by using ^{1}H NMR (300 MHz) and ^{13}C NMR (75 MHz) with Bruker Avance™ II NMR spectrometer. For both the characterization, deuterated chloroform ($CDCl_3$) served as the solvent (containing residual chloroform), and tetramethylsilane (TMS) was used as the internal standard. The chemical shift values (δ) were measured referenced to $CDCl_3$ (δ = 7.26 ppm for ^{1}H NMR and δ = 77.16 ppm for ^{13}C NMR). The following abbreviations have been used for multiplicity assignments: "s" for singlet, "d" for doublet, "t" for triplet, "m" for multiplet. ^{19}F spectra were recorded with proton decoupling. Mass spectra were collected in both MALDI-TOF with TiO2 matrix and high-resolution mass spectrometry (HRMS) performed with an ESI TOF system on a mass spectrometer.

We got the single-crystal diffraction data at 100 K using a BRUKER KAPPA APEX II CCD Duo diffractometer (operating at 50 kV, 30 mA, 1500 W) with graphite-monochromated Mo-Kα radiation (λ = 0.71073 Å). Crystal structures were solved in APEX software by direct method and refined via least squares against F2 using the SHELXL-97 software WinGX.[1,2] Details of crystal structures are available in the Cambridge Crystallographic Data Centre (CCDC) under the respective deposition numbers.

UV-vis absorption spectra were recorded in Shimadzu UV-2600 spectrophotometer. On the other hand, steady-state and time-gated emission spectra for solution and solid-state samples were measured using Fluoromax-4C (HORIBA) and Fluorolog-3 spectrometer (HORIBA), respectively. All photoluminescence quantum yield (PLQY) values reported were obtained through absolute quantum yield measurements using an integrating sphere (K-sphere, HORIBA). Time-resolved photoluminescence (PL) decay profiles were performed utilizing time-correlated single photon counting (TCSPC) and multi-channel scanning (MCS) techniques by using either a 402 nm diode laser (nanoLED, HORIBA) source for time windows shorter than 25 µs or a 366 nm spectraLED source for longer lifetime in FluoroCube (HORIBA). All data analysis was done using DAS6, a fluorescence decay analysis software (HORIBA), with the quality of fit assessed through χ^2-values and visual inspection of residuals where the fit with χ^2 = (1-1.2) value is taken as the best fit.

Section S2: Materials

For synthesis purposes, all the important precursors like tetrafluoroterephthalonitrile, tetrafluorophthalonitrile, 3,5-ditertbutylbenzene-1,2-diol, potassium carbonate, etc., are purchased from Sigma-Aldrich and were used without any further purification. For spectroscopic studies in solution, spectroscopic grade solvents were purchased from Spectrochem. Pvt. Ltd. India. For solid-state emission studies, crystals or powdered samples were held in between two quartz slides purchased from TED PELLA INC.

Section S3: Experimental and theoretical Methods

Fabrication of 10wt% PMMA films:

9 mg of PMMA and 1 mg of emitter were weighed to make 10 wt% emitter-doped PMMA. The compounds were dissolved in 1 ml spectroscopic grade chloroform by heating at 50°C for 10 minutes followed by sonication for another 5 minutes. Thereafter 0.2 ml of the resulting solution is used for spin-coating on a quartz plate at 1000 rpm for 120 seconds. The quartz plate was then kept in high vacuum for another 1 hour prior to photophysical studies.

Preparation of Neat Films:

Neat films were prepared by dissolving 1 mg of each luminogens in 0.5 ml of spectroscopic-grade chloroform. The resulting solution was spin-coated onto quartz plates at 1000 rpm for 120 seconds. The coated plates were dried under a high vacuum for 1 hour before photophysical measurements.

Density Functional Theory (DFT) Calculations:

Quantum chemical computations were achieved using Gaussian 09 software [3] on a high-performance cluster at IISER Pune. Initial geometry optimizations of luminogens were conducted in the gas phase, followed by frequency analyses [4,5] at the PBE0/6-31 G (d,p) level to confirm minima on the potential energy surface. Time-dependent DFT (TD-DFT) calculations were performed to examine HOMO-LUMO distributions and singlet/triplet state energies.

QM/MM Calculations:

The quantum mechanics/molecular mechanics (QM/MM) model was built based on the single crystal structure and was utilized to analyse the electronic properties of the active QM molecule embedded in the aggregated crystal state. The surrounding molecules were defined as a rigid MM component to simulate the effect of a solid-state environment.[6] The high layer for QM was computed using the TD-DFT method of PBE0/6-31G (d, p) level, and the low layer for MM is described by the universal force field (UFF)[7] augmented by Coulomb interactions which are in accordance with the quantum method. The monomer was configured to a high layer for QM calculated by the TD-DFT method of PBE0/6-31G (d, p) level.

Spin-orbit coupling matrix element calculation:

SOC values between excited singlet and triplet states were calculated using the zeroth-order regular approximation (ZORA). In this approximation, the SOC operator, $\widehat{H}_{SOC},$ is described as[8]

$$\widehat{H}_{SOC} = \frac{c^2}{(2c^2-v)^2}\sigma \cdot (\nabla v \times p)$$

Where c, v, σ, and p signify the speed of light, Kohn-Sham potential, Pauli spin-matrix vector, and linear momentum operator respectively. Perturbations were applied to scalar relativistic orbitals post-SCF and TD-DFT calculations. PBE0 functional was employed, and the computations were conducted using the PYSOC program.

SectionS4: Cell culture and drug treatments

MCF7 cells were purchased from the European Collection of Cell Cultures (ECACC). The cells were maintained in 60 mm dishes (VWR, USA) and grown in Dulbecco's Modified Eagle Medium (DMEM; HiMedia, India) containing 4.5g/L Glucose, supplemented with 10% heat inactivated FBS (Thermo Scientific, USA),1X Pen-Strep antibiotic (Thermo Scientific, USA) and incubated at 37°C in a humidified incubator with 5% CO2 (Eppendorf, GmbH).

For the MTT cell viability assay, 7.5 x 10^3 MCF-7 cells were seeded per well in a 96-well plate (Eppendorf, Gm) and incubated at 37°C with 5% CO2 for 18 hours. Cells were treated with different concentrations of the compounds *o*CN1, *o*CN2, and *p*CN or DMSO vehicle control (5

µM, 10 µM, and 15 µM). Post 24 hrs of treatment, drug-containing media was removed, and 100 µL of DMEM containing 0.5 mg/ml MTT (Hi Media, India) was added to each well and incubated at 37ºC in the dark for 4 hours. Post incubation, the MTT-DMEM solution was aspirated, and the purple MTT-formazan crystals were solubilized in 100 µL of DMSO and incubated for 15 mins in the dark before recording the absorbance at 570 nm using the EnSight Multimode plate reader (Revvity, USA).

For the two-photon imaging experiments, 0.2 x 10^6 MCF-7 cells were seeded on coverslips (pre-washed with 95% EtOH) in 35 mm culture dish (VWR, USA) and incubated at 37°C with 5% CO2 for 18 hrs. At 70% confluency, cells were treated with 10 µM of compound (*o*CN1, *o*CN2 and *p*CN) or DMSO and incubated for 24 hrs. post-treatment, cells were washed twice with 1X DPBS and fixed with 4% para-formaldehyde for 20 minutes in the dark. After fixation, cells were washed twice with PBS and mounted onto slides. The cells were imaged on a multiphoton confocal microscope at an excitation wavelength of 800 nm (Leica, Germany) and observed with a 63X oil immersion objective.

SectionS5: Synthesis and Characterization

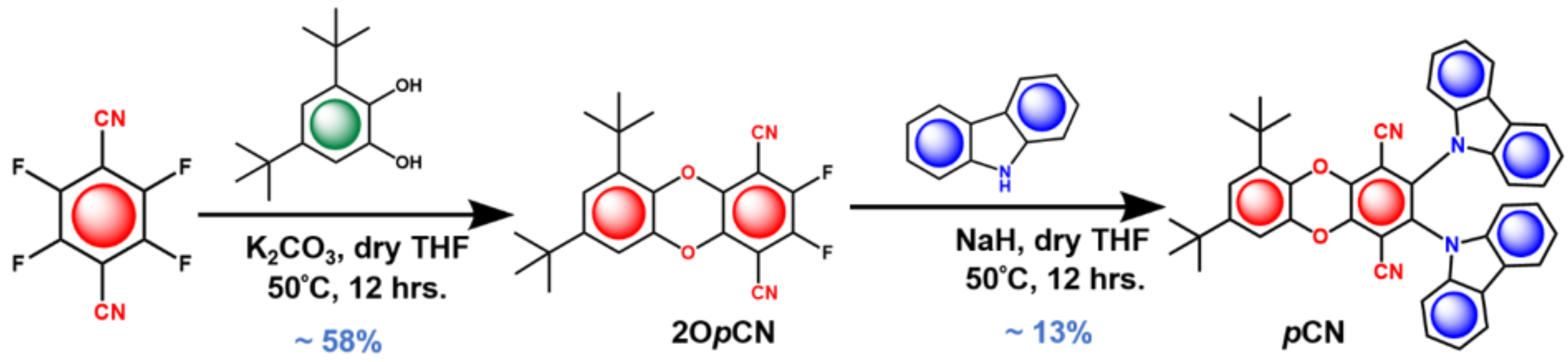

Scheme S1: Synthetic routes of *p*CN compound

Synthesis 2O*p*CN:

In a two-necked round bottom (RB) flask containing with a magnetic stir bar, 1gm (5mmol, 1eq.) of commercially available tetrafluoroterepthalonitrile (4F*p*CN, $C_8F_4N_2$) and 1.11gm (5mmol, 1eq.) of 3,5-ditertbutylbenzene-1,2-diol($C_{14}H_{22}O_2$) were taken as precursors. Then 20 ml of anhydrous tetrahydrofuran (THF) under a N2-atmosphere was added to the RB flask with a syringe. Slowly, 1.38gm (10mmol, 2eq.) of vacuum-dried potassium carbonate (K_2CO_3) was poured into that mixture, which was then stirred at 50^0C for 12 hours under N2-atmosphere (**Scheme S1**). The formation of a new product is confirmed by thin-layer chromatography (TLC). Excess potassium carbonate was quenched by the distilled water added dropwise to the RB. The mixture is then washed several times with water and DCM and the organic layer was extracted with sodium sulphate (Na_2SO_4). After the evaporation of the solvent, we got the pure product 2O*p*CN ($C_{22}H_{20}F_2N_2O_2$) that had been confirmed by ^{1}H-NMR,^{13}C-NMR and mass spectroscopy. Yield ~ 58%.

Characterization data: ^{1}H NMR (400 MHz, $CDCl_3$): δ 7.08 (d, 1H, J=2.5Hz), 6.93(d, 1H, J=2.5Hz), 1.45(s, 9H), 1.29(s, 9H) ppm.

^{13}C{^{1}H} NMR (101 MHz, $CDCl_3$): δ 147.96, 145.83, 145.67, 145.56, 143.24, 143.13, 142.96, 140.65, 140.53, 138.25, 137.30, 134.71, 119.77, 111.44, 107.27, 106.88, 106.88, 106.84, 95.21, 34.21, 33.84, 30.13, 28.80 ppm.

MALDI-TOF: Calculated mass for $C_{22}H_{20}F_2N_2O_2$ is 382.1493, found 383.2573.

Synthesis of *p*CN:

In a two-necked round RB flask with a magnetic stir bar, 481mg (2.8mmol, 2.2eq.) of carbazole was dissolved in 10 ml of anhydrous THF under an N2 atmosphere. Slowly, 118mg (4.92mmol, 3.76eq.) of vacuum-dried sodium hydride (NaH) (60% in oil) was added to that RB, which was then stirred at room temperature for 30 minutes. After this time, 500mg (1.31mmol, 1eq.) of 2O*p*CN was poured to the reaction mixture, and stirring was continued for another 12 hours at 50°C under N2-atmosphere (see **Scheme S1**). The completion of the reaction is confirmed by thin-layer chromatography (TLC). Excess sodium hydride was quenched by adding distilled water dropwise. The mixture was then washed several times with water and DCM. Finally, the crude product was purified using silica gel flash column chromatography with a hexane/dichloromethane (DCM) solvent system and which had been confirmed by ^{1}H-NMR, ^{13}C-NMR and mass spectroscopy. Yield ~ 13%.

Characterization data: ^{1}H NMR (400 MHz, $CDCl_3$): δ 7.69-7.66 (4H, m), 7.16 (1H, d, J=4Hz), 7.05-6.95 (13H, m,), 1.53(9H, s), 1.34(9H, s) ppm.

^{13}C{^{1}H} NMR (101 MHz, $CDCl_3$): δ 148.87, 145.84, 145.66, 139.57, 138.69, 138.64, 138.41, 136.04, 133.28, 125.29, 125.26, 123.79, 123.77, 120.87, 120.61, 119.93, 112.51, 110.75, 110.43, 109.60, 109.57, 106.81, 35.17, 34.77, 31.04, 29.78 ppm.

HRMS (ESI): m/z calcd. for [$C_{46}H_{36}N_4O_2$] $(M+H)^+$ 677.2918, found 677.2911.

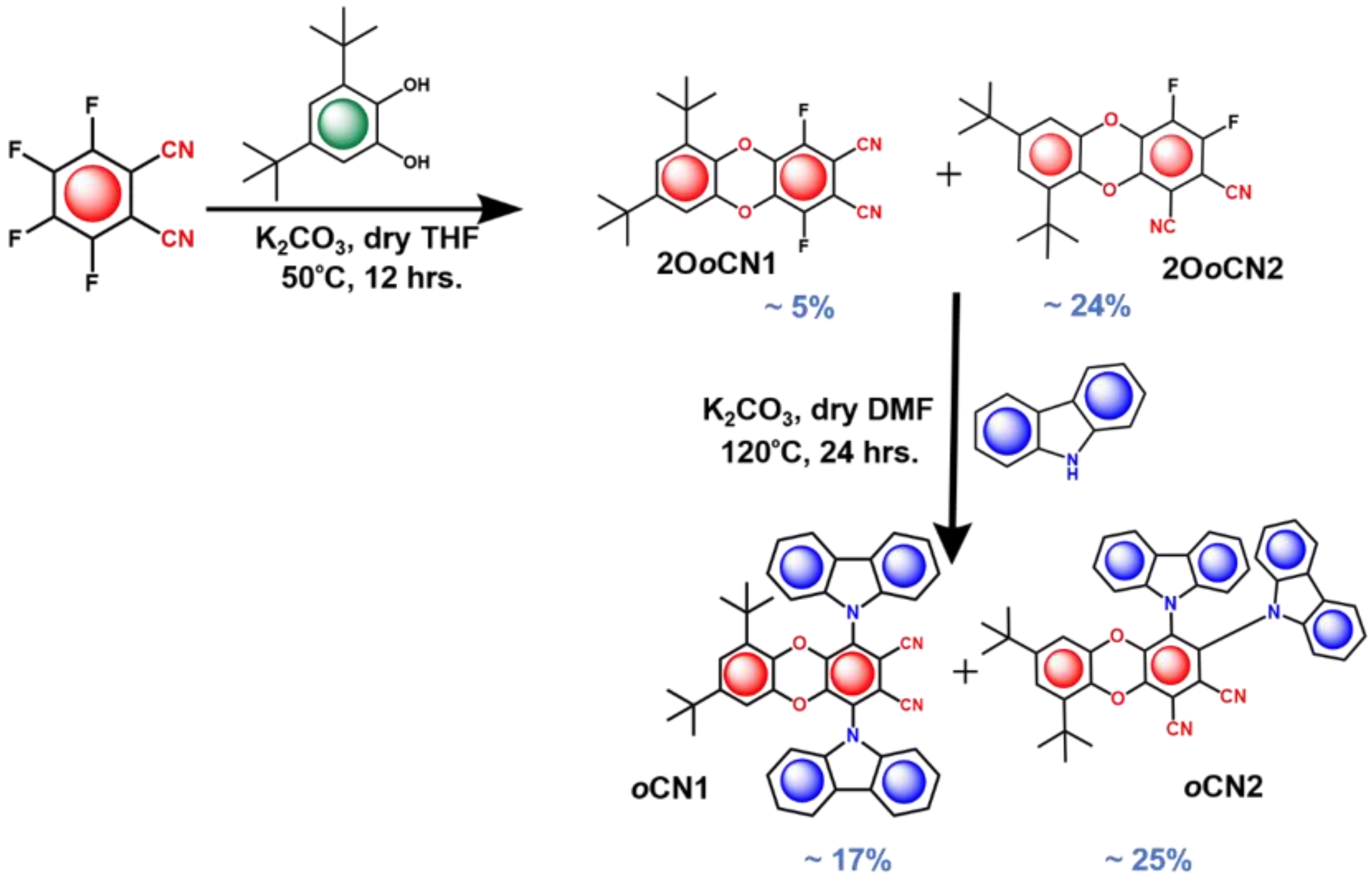

Scheme S2: Synthetic routes of *o*CN1 and *o*CN2 compound

Synthesis of 2O*o*CN1 and 2O*o*CN2:

In a two-necked RB flask equipped with a magnetic stir bar, 1gm (5mmol, 1eq.) of commercially available tetrafluoropthalonitrile (4FOCN, $C_8F_4N_2$) and 555mg (2.5mmol, 0.5eq.) of 3,5-ditertbutylbenzene-1,2-diol($C_{14}H_{22}O_2$) were taken as precursors. Then 20 ml of anhydrous tetrahydrofuran (THF) under a N2-atmosphere was added to the RB flask with a syringe. Slowly, 830mg (6mmol, 1.2eq.) of vacuum-dried potassium carbonate (K_2CO_3) was poured into that mixture, which was then stirred at 50^0C for 12 hours under N2-atmosphere (**Scheme S2**). The formation of a new product is confirmed by thin-layer chromatography (TLC). Excess potassium carbonate was quenched by the distilled water added dropwise to the RB. The mixture is then washed several times with water and DCM. Finally, the crude product was purified using silica gel flash column chromatography with a hexane/dichloromethane (DCM) solvent system and without further characterization we proceeded for next step. As the colour and polarity of these two isomers are quite similar, so we can't separate these two isomers through flash column. Yield of 2OoCN1 ~ 5% and 2OoCN2 ~ 24 %.

MALDI-TOF: Calculated mass for $C_{22}H_{20}F_2N_2O_2$ is 382.1493, found 383.2917.

Synthesis of *o*CN1 and *o*CN2:

In a two-necked round RB flask with a magnetic stir bar, 481mg (2.8mmol, 2.2eq.) of carbazole was dissolved in 10 ml of anhydrous DMF under an N2 atmosphere. Slowly, 118mg (4.92mmol, 3.76eq.) of vacuum-dried potassium carbonate (K_2CO_3) was added to that RB, which was then stirred at room temperature for 30 minutes. After this time, 500mg (1.31mmol, 1eq.) of 2O*o*CN1 and 2O*o*CN2 were poured to the reaction mixture, and stirring was continued for another 24 hours at 120°C under N_2-atmosphere (see **Scheme S2†**). The completion of the reaction is confirmed by thin-layer chromatography (TLC). Excess potassium carbonate was quenched by adding distilled water dropwise. The mixture was then washed several times with water and EtOAc. Finally, the crude product was purified using silica gel flash column chromatography with a hexane/Ethyl Acetate (EtOAc) solvent system and which had been confirmed by ^{1}H-NMR,^{13}C-NMR and mass spectroscopy. Yield of oCN1~ 17% and oCN2 ~ 25%.

Characterization data of *o*CN1: ^{1}H NMR (400 MHz, $CDCl_3$): δ 8.16 (d, 3H, J=10Hz), 7.49-7.34 (m, 7H), 7.17-7.06 (m, 5H), 6.95 (d, 1H, J=2.1), 6.86 (d, 1H, J=2.15), 6.41 (d, 1H, J=2.9), 1.51 (s, 9H), 1.16 (s, 9H) ppm.

^{13}C{^{1}H} NMR (101 MHz, $CDCl_3$): δ 158.35, 155.73, 148.71, 146.03, 145.56, 140.48, 139.73, 139.70, 137.58, 126.73, 126.61, 124.51, 124.43, 122.03, 121.72, 121.59, 120.89, 120.27, 112.47, 110.07, 109.64, 109.58, 34.38, 30.97, 28.54 ppm.

HRMS (ESI): m/z calcd. for [$C_{46}H_{36}N_4O_2$] $(M+H)^+$ 677.2918, found 677.2919.

Characterization data of *o*CN2: ^{1}H NMR (400 MHz, $CDCl_3$): δ 8.22 (dd, 3H, J=22.6Hz), 7.69 (d, 1H, J=10Hz), 7.52-7.32 (m, 10H), 7.08 (t, 1H, J=15.5), 7.00 (t, 1H, J=14.08), 6.84 (d, 1H, J=10.2Hz), 6.75 (s, 1H) 1.03 (s, 9H), 0.41 (s, 9H) ppm.

^{13}C{^{1}H} NMR (101 MHz, $CDCl_3$): δ 157.91, 154.14, 148.70, 146.01, 140.47, 139.78, 137.58, 130.11, 130.07, 126.59, 126.06, 124.53, 122.03, 121.57, 120.87, 120.09, 114.23, 110.06, 109.56, 34.64, 34.37, 31.15, 28.57 ppm.

HRMS (ESI): m/z calcd. for [$C_{46}H_{36}N_4O_2$] $(M+H)^+$ 677.2918, found 677.2905.

^{1}H NMR of 2O*p*CN:

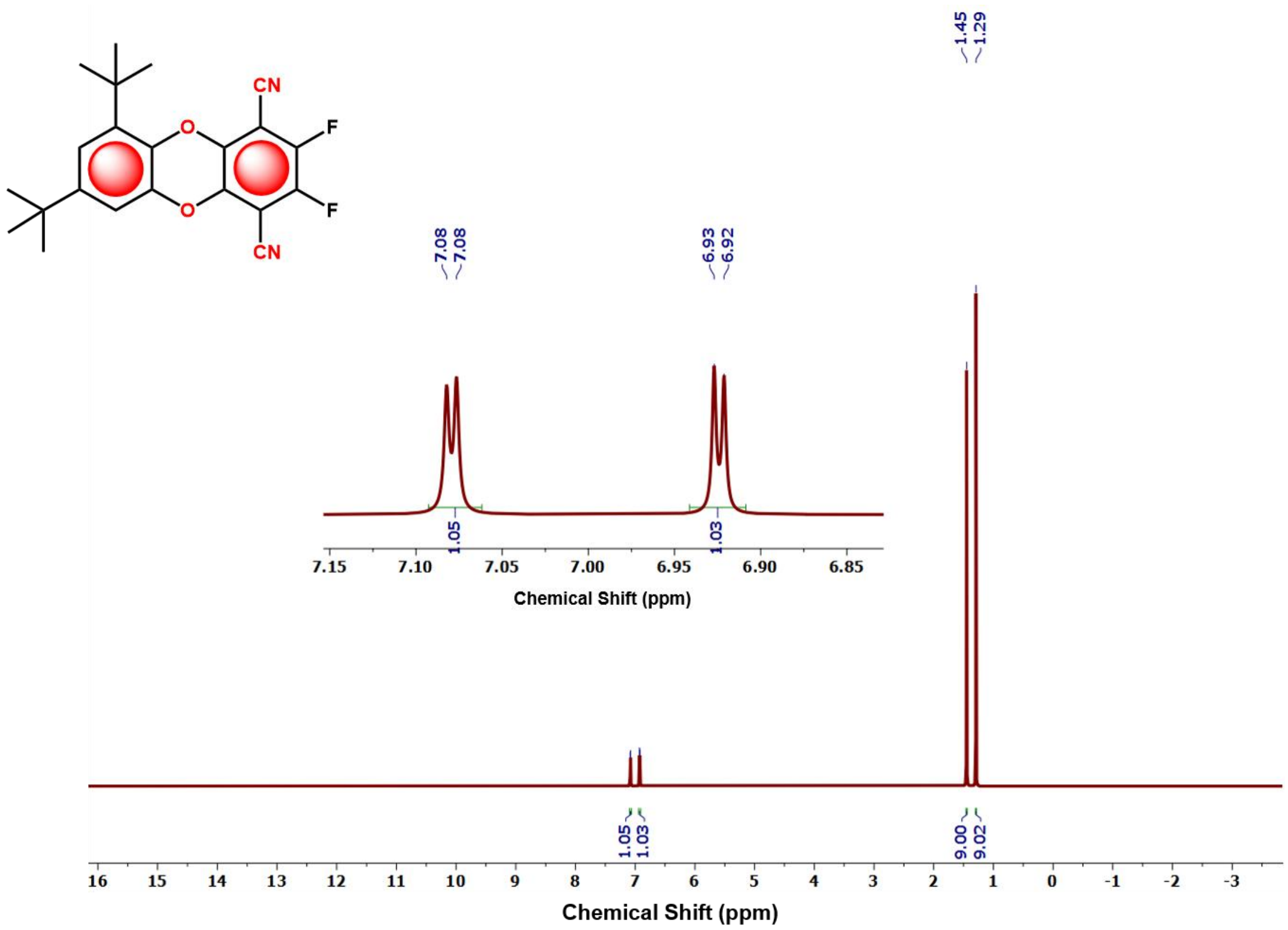

^{13}C NMRof 2O*p*CN:

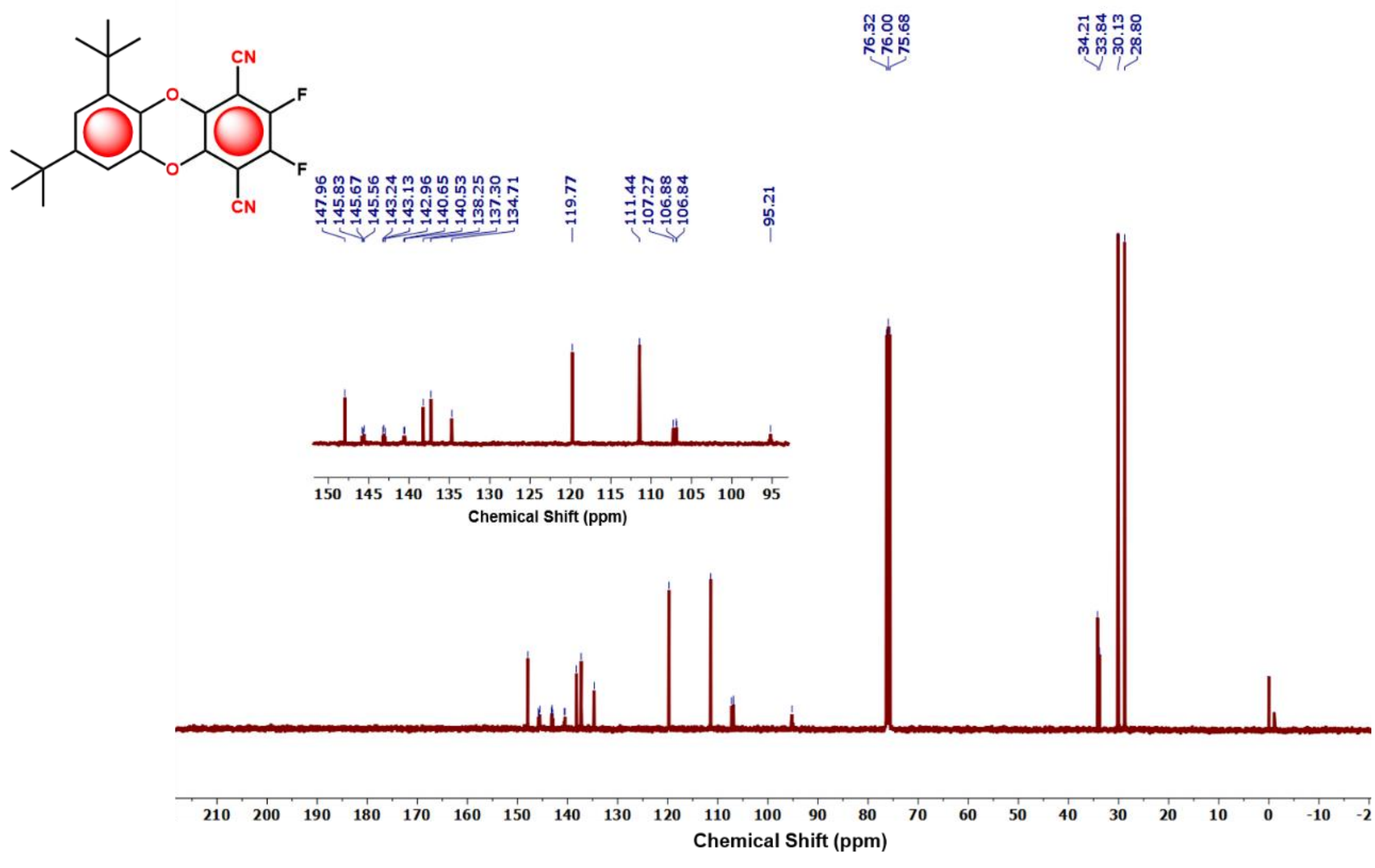

MALDI-TOF of 2O*p*CN:

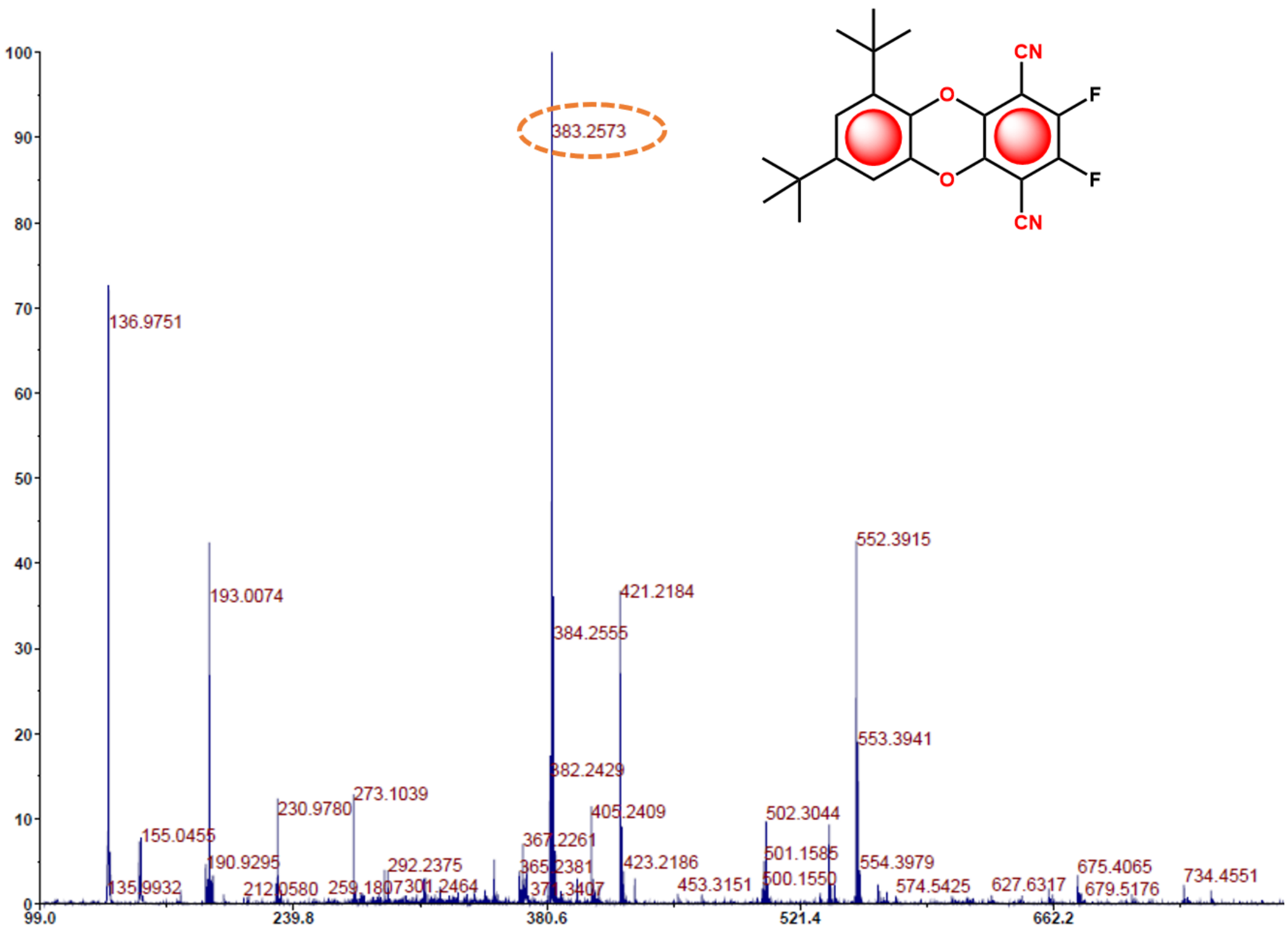

^{1}H NMR of *p*CN:

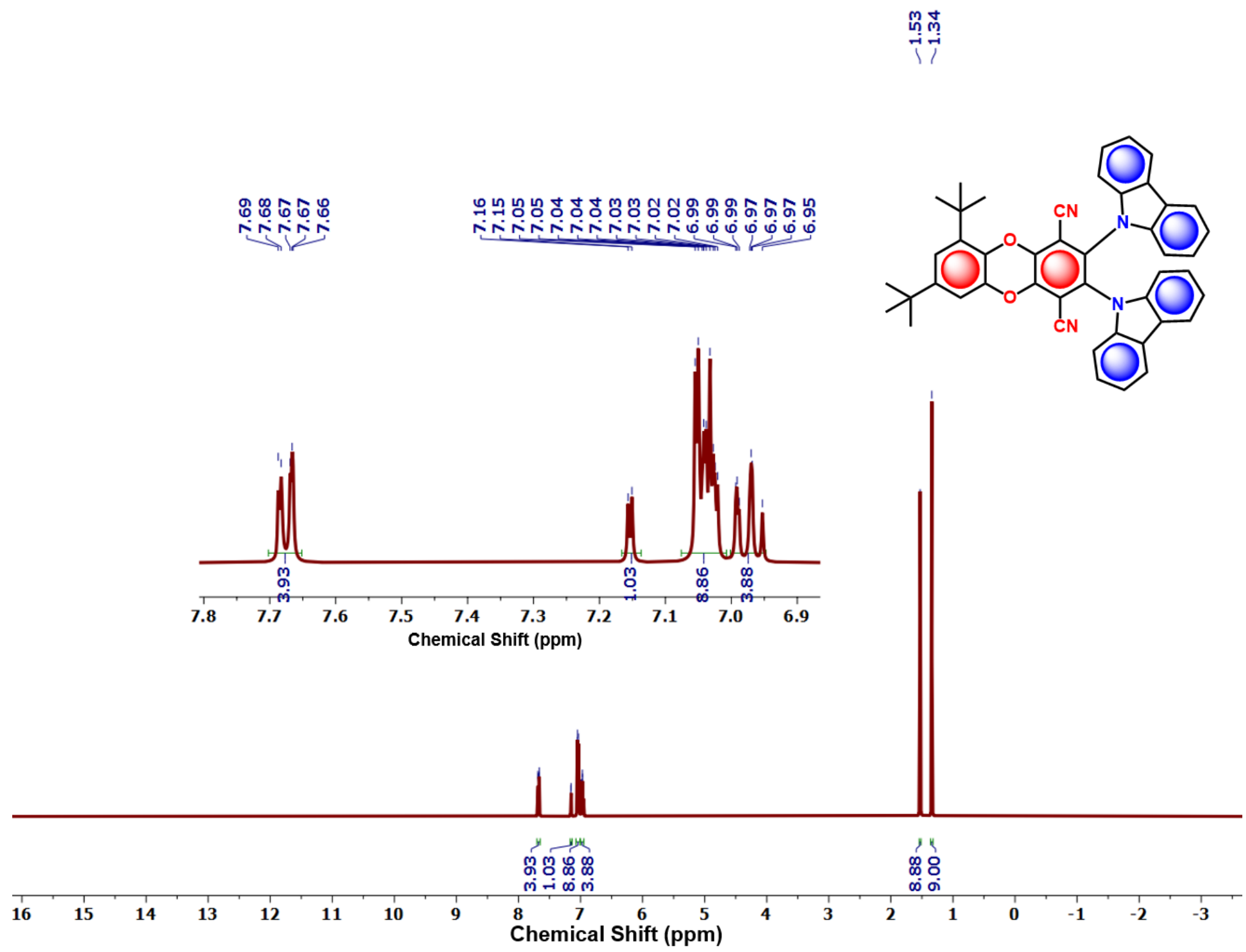

^{13}C NMR of *p*CN:

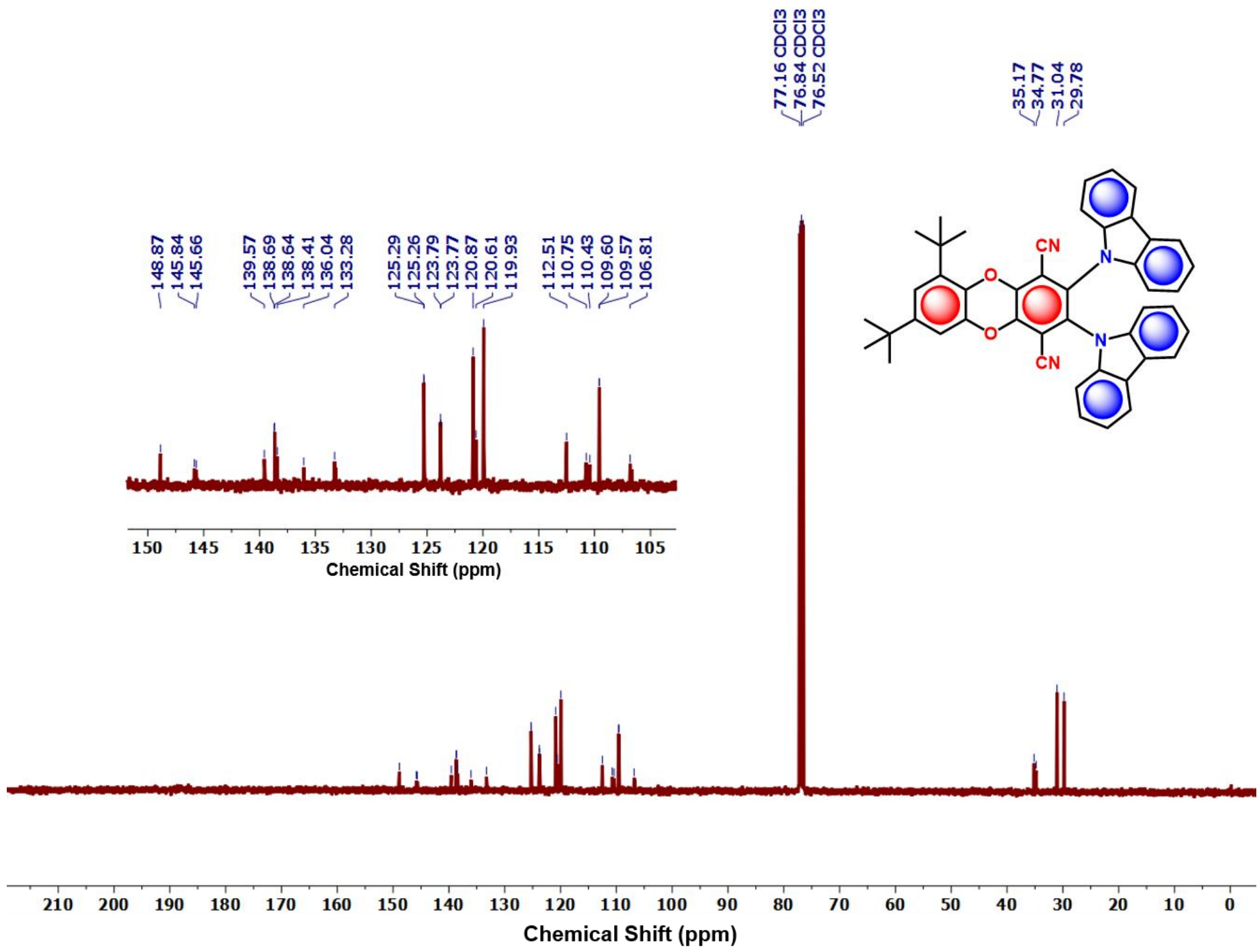

HRMS of *p*CN:

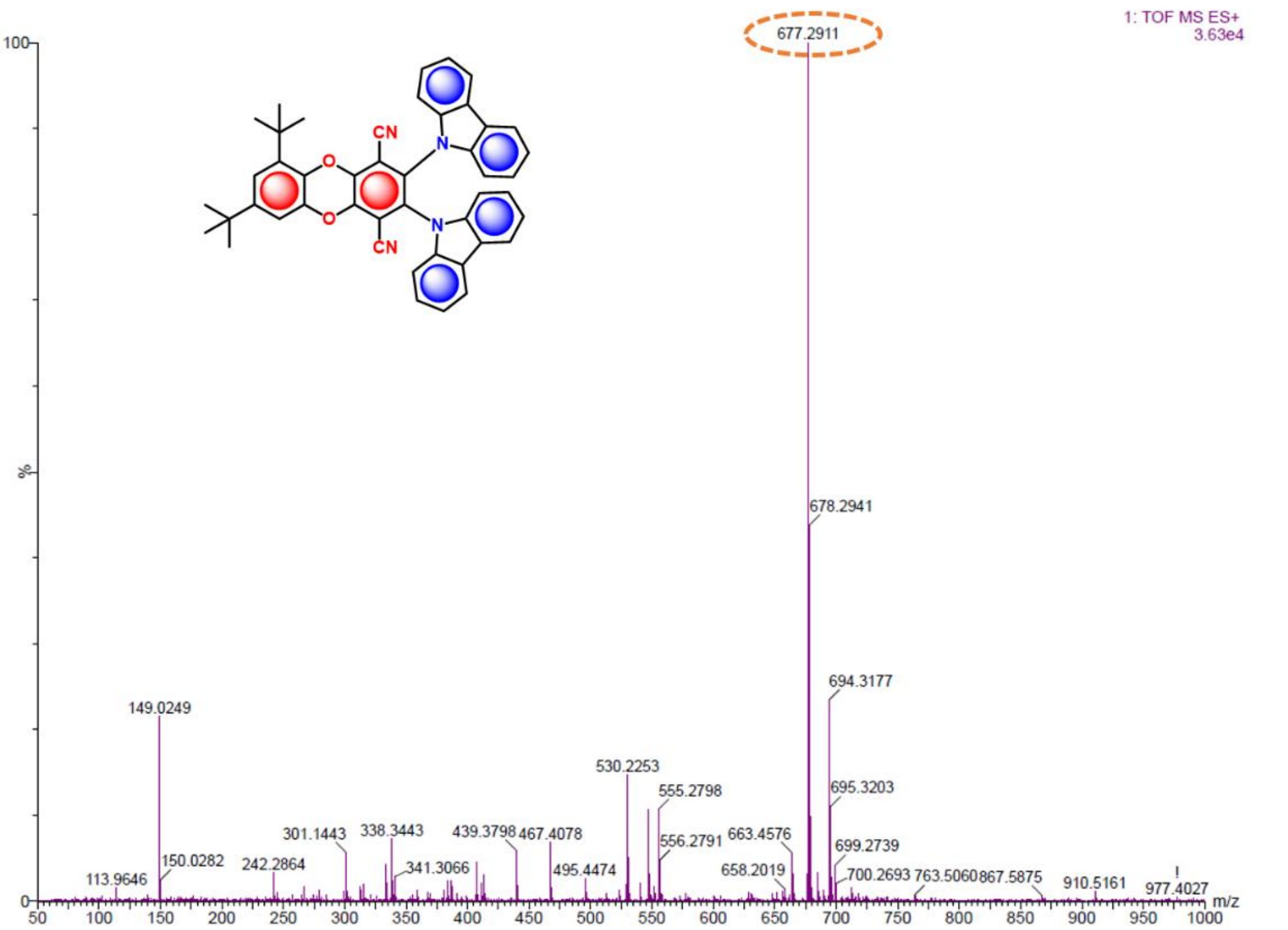

MALDI-TOF of 2O*o*CN1 and 2O*o*CN2:

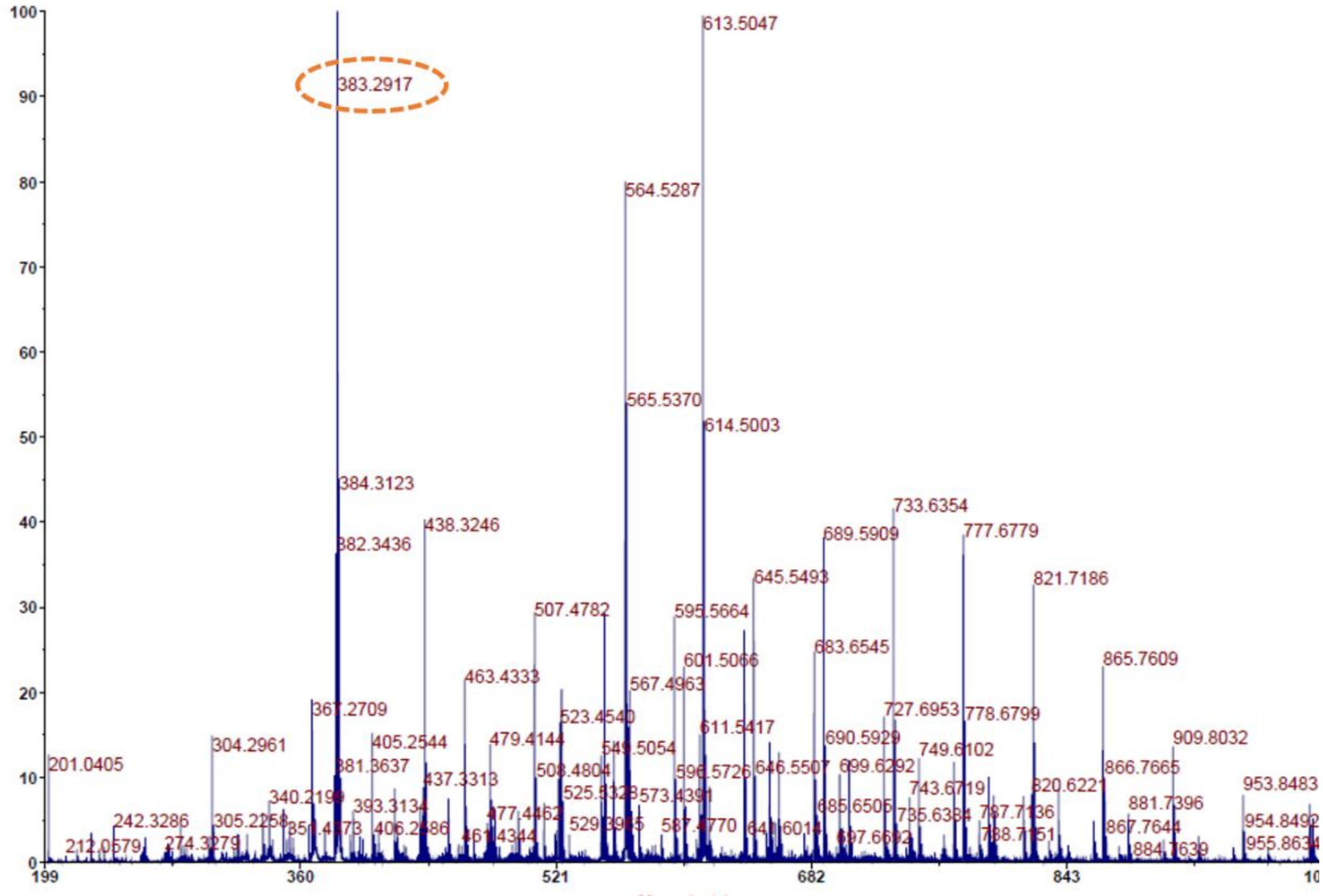

^{1}H NMR of *o*CN1:

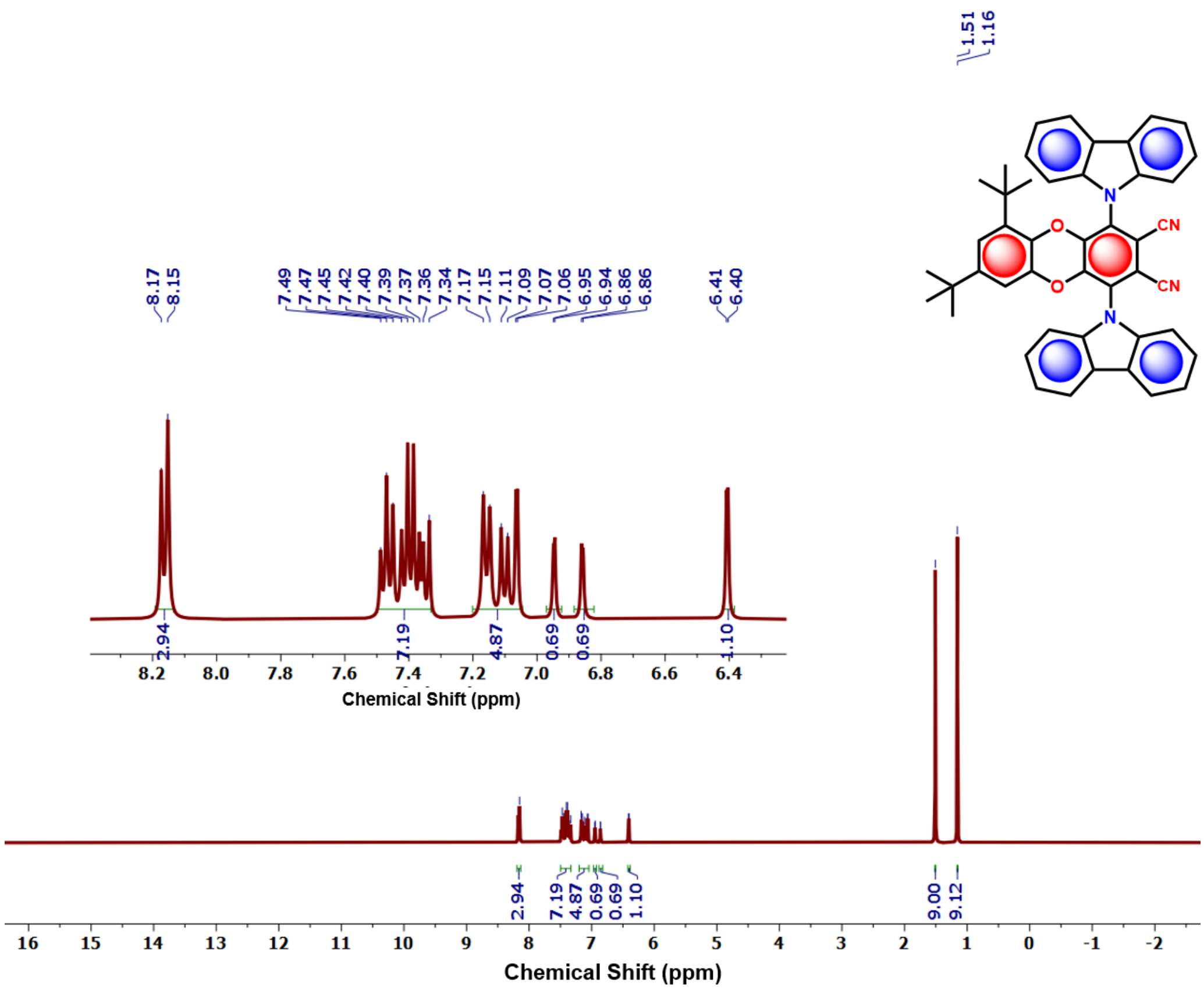

^{13}C NMR of *o*CN1:

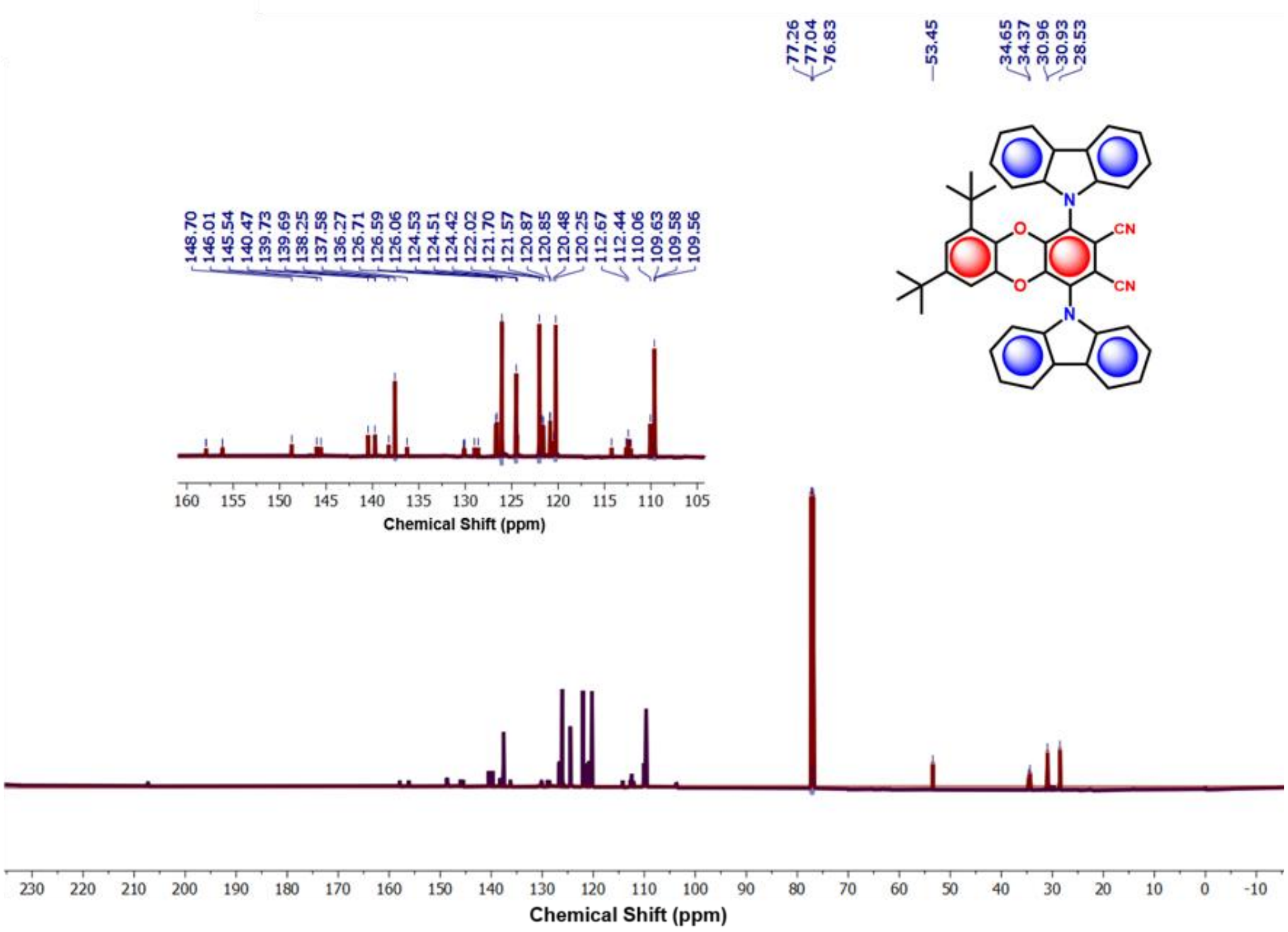

HRMS of *o*CN1:

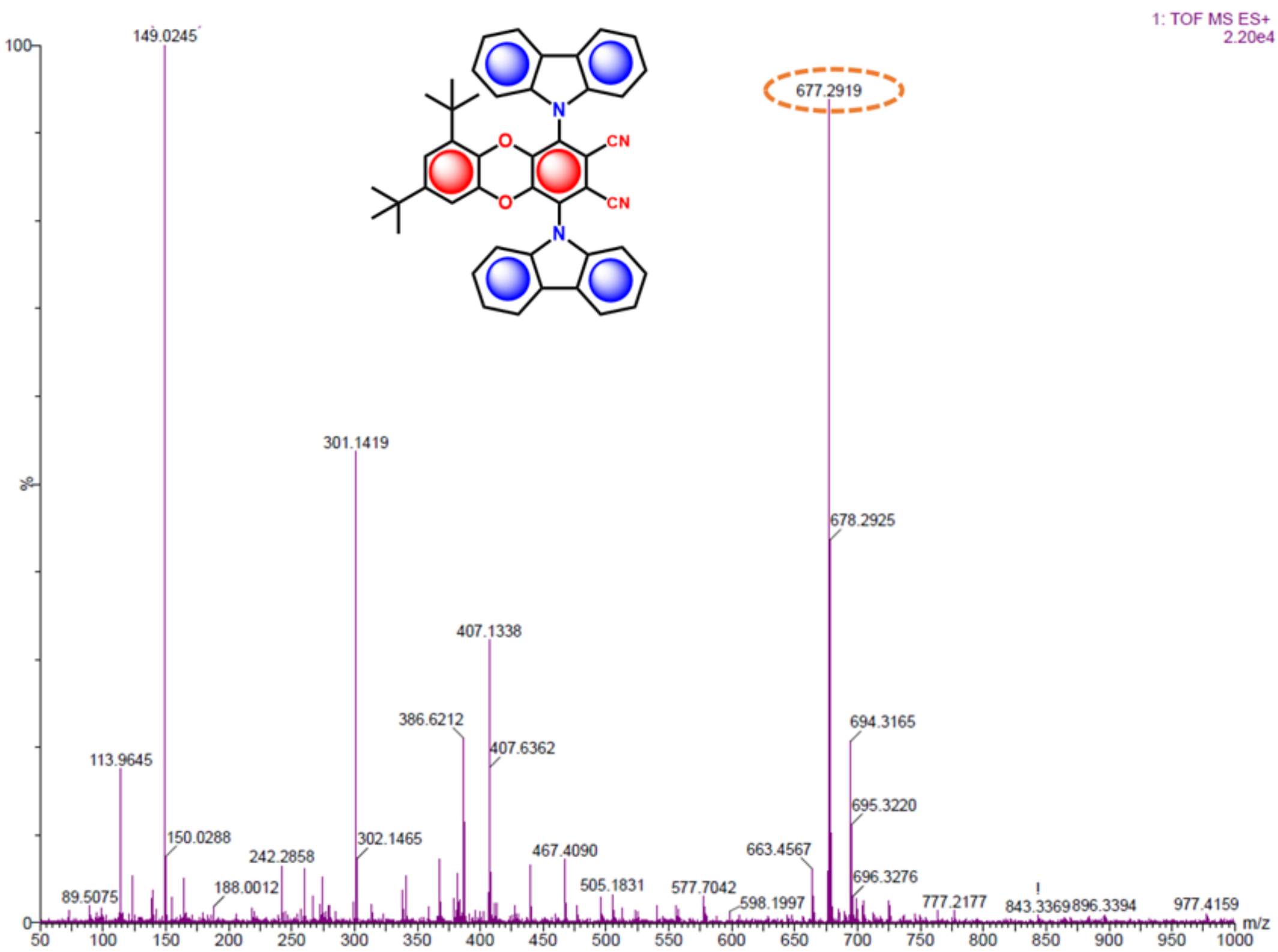

^{1}H-NMR of *o*CN2:

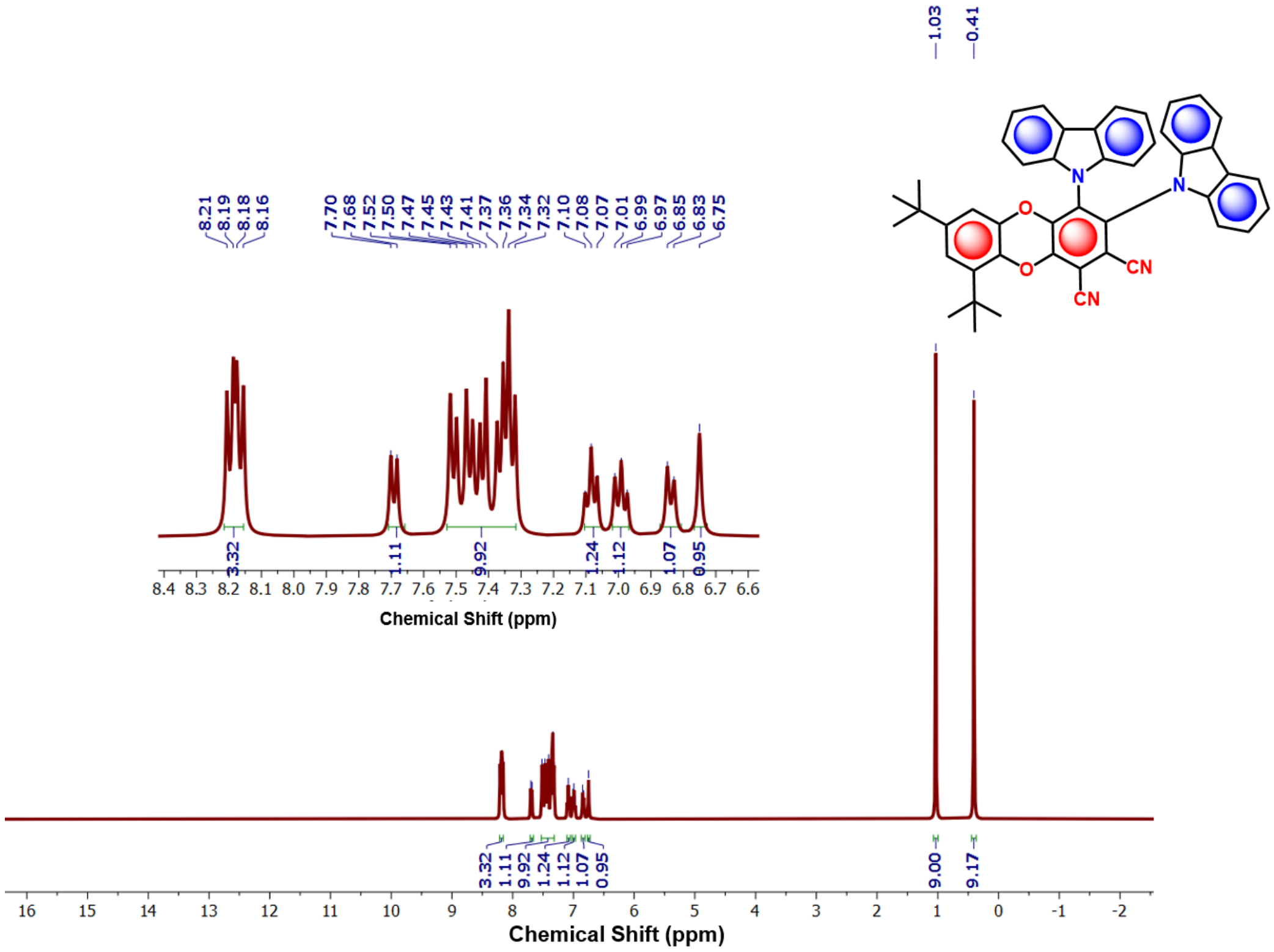

^{13}C NMR of *o*CN2:

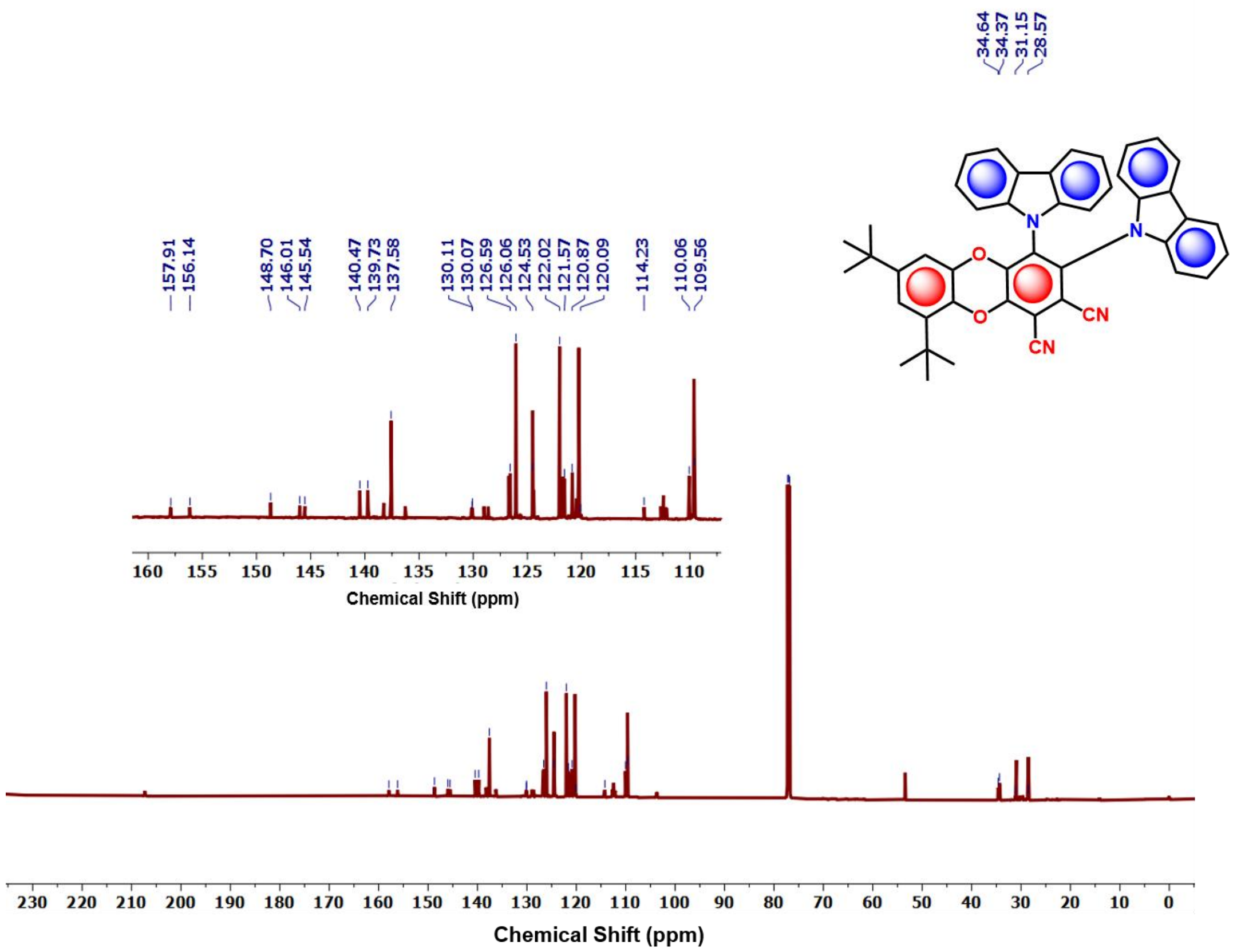

HRMS of *o*CN2:

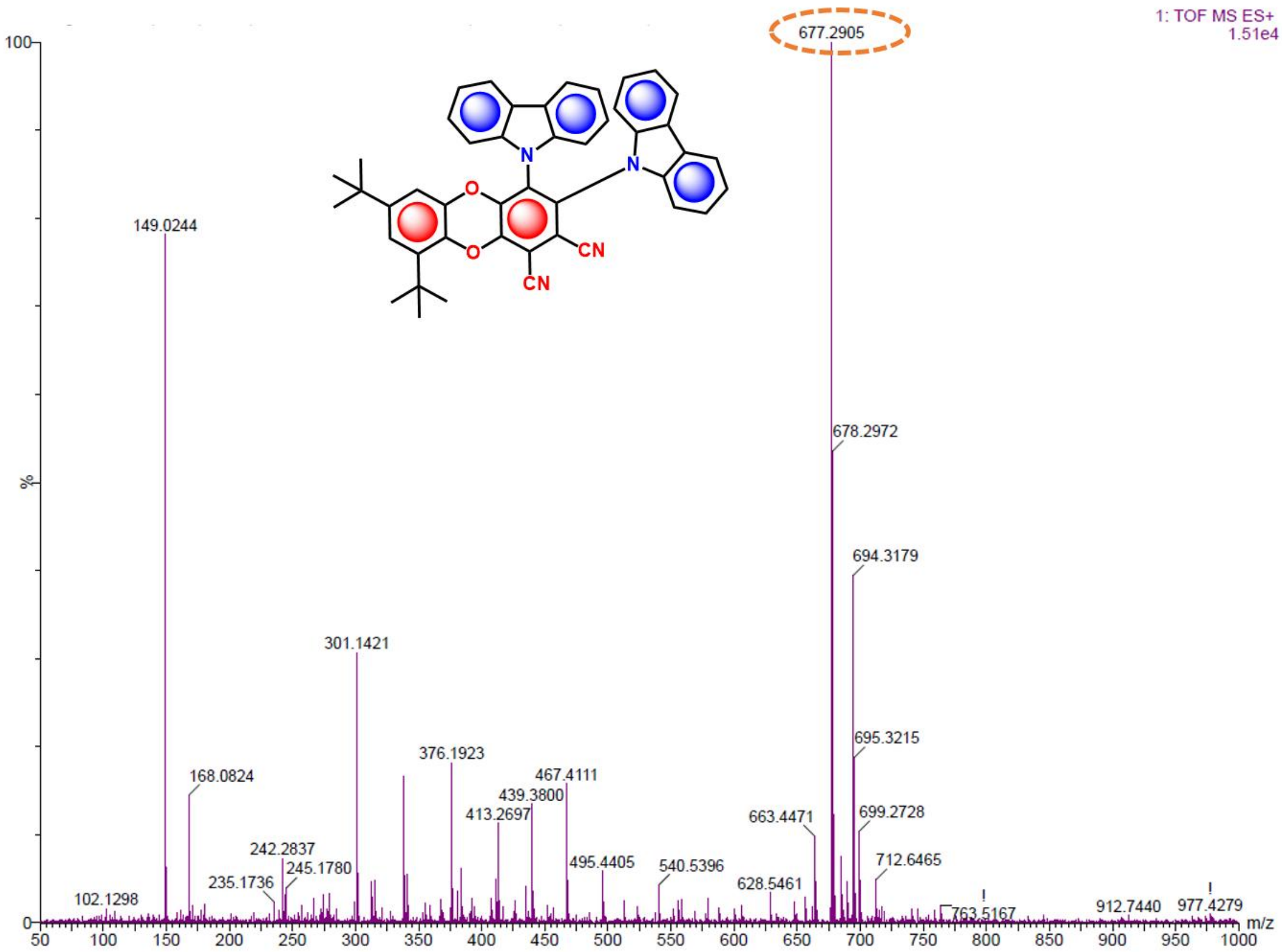

1: TOF MS ES+
1.51e4
677.2905
149.0244
678.2972
694.3179
301.1421
695.3215
376.1923
168.0824
467.4111
439.3800
413.2697
663.4471
699.2728
242.2837
245.1780
235.1736
495.4405
540.5396
712.6465
628.5461
102.1298
763.5167
912.7440
977.4279
CN
CN
N
N
O
O
%
m/z

Section S6: Photophysical and Theoretical Studies

Absorption, emission spectra in toluene and solvatochromic study:

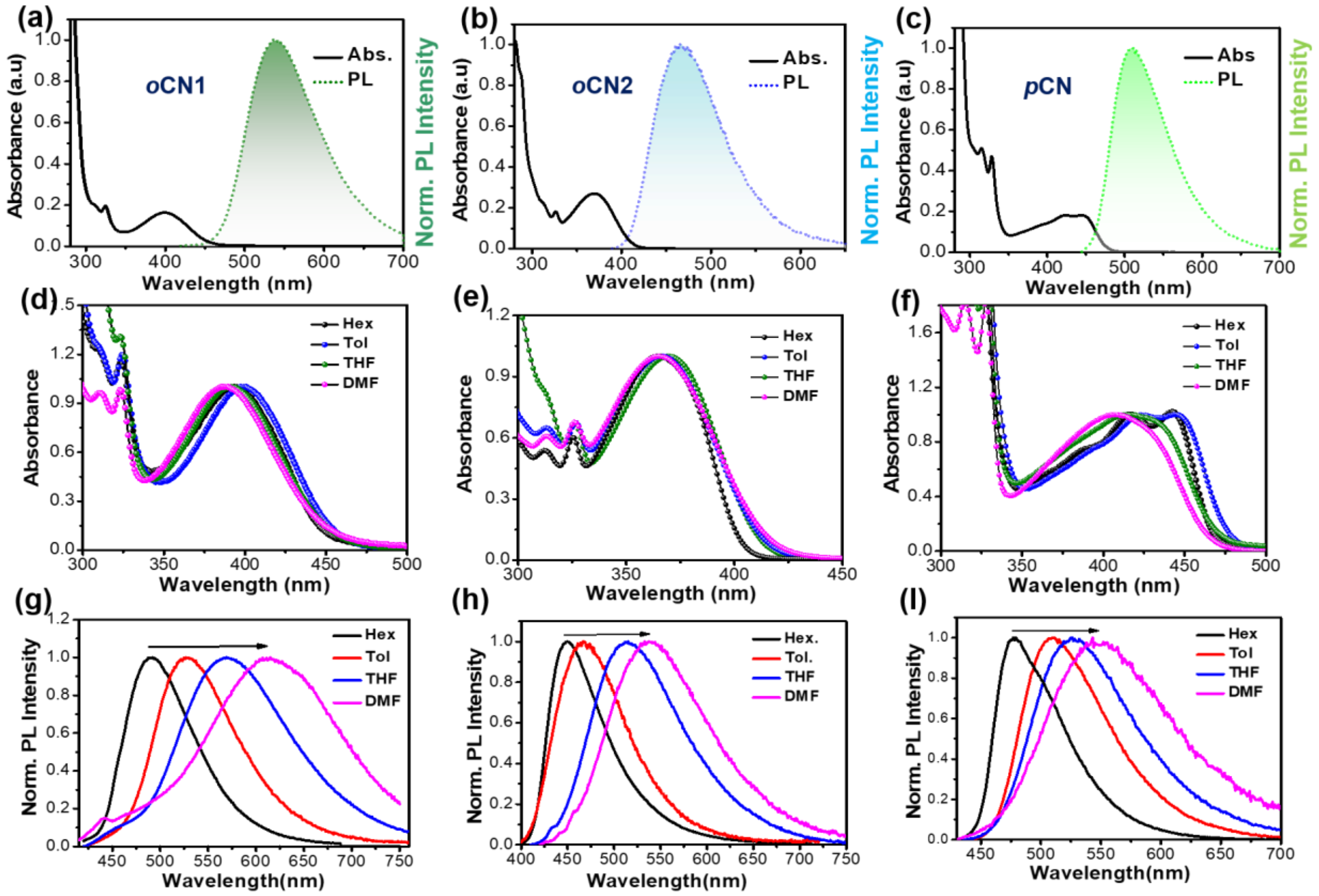

Fig. S1 Absorption (without normalization, white portion) and emission spectra (shaded portion) of (a) ***o*CN1**, (b) ***o*CN2**, and (c) ***p*CN**, respectively. Polarity-dependent absorption and emission studies in different solvents at RT of (d), (g) ***o*CN1**, (e), (h) ***o*CN2** and (f), (l) ***p*CN** (concentration 30 µm).

HOMO-LUMO energy distribution, TD-DFT and SOC calculation in toluene solvent:

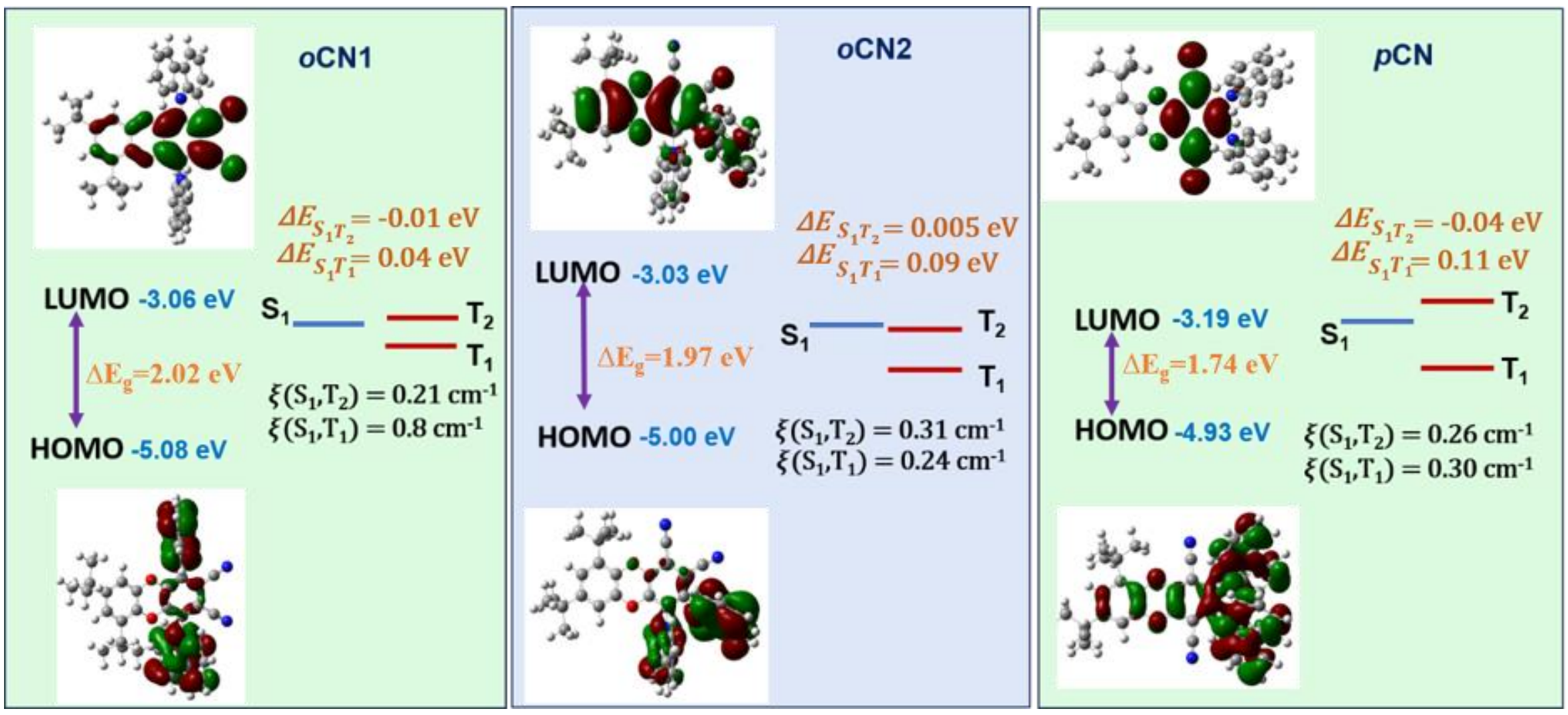

Fig. S2 HOMO and LUMO molecular orbitals (ground-state geometry at the PBE0/6-31G(d,p) level) along with the energy gaps and the electronic energy states (TD-DFT using the PBE0 functional together with the 6-31G(d,p) basis set) in ***o*CN1**, ***o*CN2** and ***p*CN**.

Ground state molecular optimization:

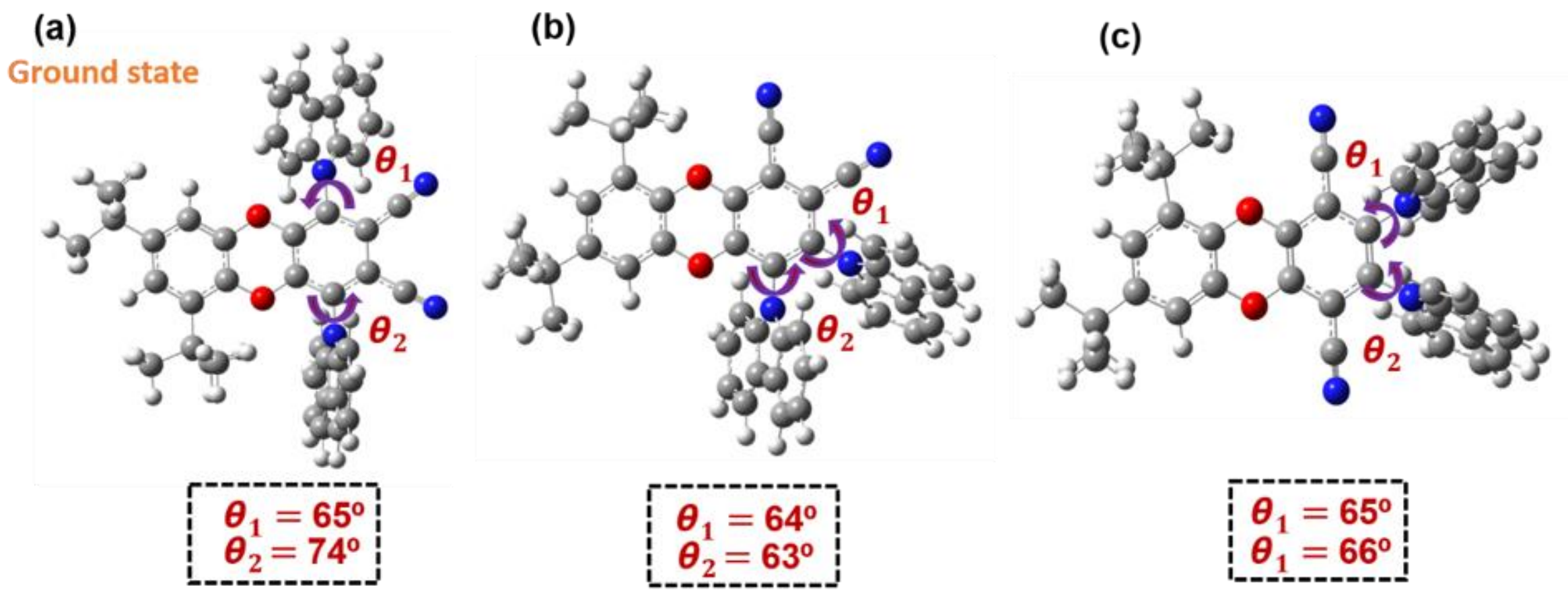

Fig. S3 Optimized ground state structures of all three isomers in the solvent-based (IEFPCM/Toluene) model at PBE0/ 6-31G (d,p) level of theory (a) ***o*CN1**, (b) ***o*CN2** and (c) ***p*CN**.

Lippert-Mataga Plot:

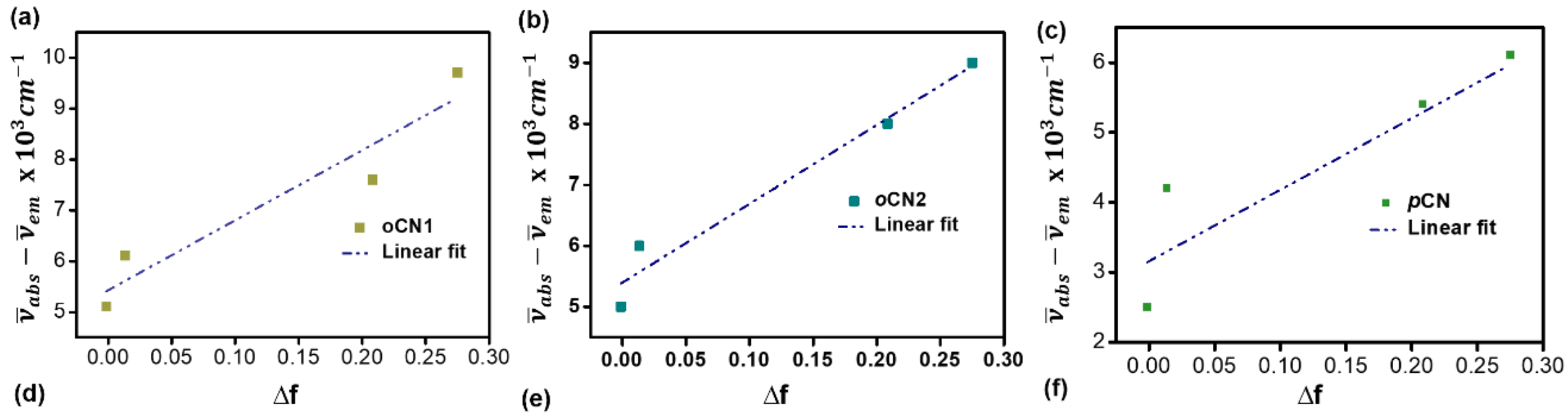

(d)

Equation	y = a + b*x		
Weight	No Weighting		
Residual Sum of Squares	1.05E+06		
Pearson's r	0.95513		
Adj. R-Square	0.86842		
		Value	Standard Error
a	Intercept	5418.606	521.1973
b	Slope	13761.24	3017.329

(e)

Equation	y = a + b*x		
Weight	No Weighting		
Residual Sum of Squares	340467.3		
Pearson's r	0.98283		
Adj. R-Square	0.94893		
		Value	Standard Error
a	Intercept	5397.616	296.3255
b	Slope	12922.45	1715.495

(f)

Equation	y = a + b*x		
Weight	No Weighting		
Residual Sum of Squares	9.23E+05		
Pearson's r	0.93145		
Adj. R-Square	0.80139		
		Value	Standard Error
a	Intercept	3157.391	487.7796
b	Slope	10222.65	2823.866

Fig. S4 Plot and fit line of $\bar{\nu}_{abs} - \bar{\nu}_{em}$ (Stokes shift, cm^{-1}) with Lippert–Mataga solvent polarity function **Δf** for (a), (d) ***o*CN1**, (b), (e) ***o*CN2** and (c), (f) ***p*CN** in series of different polarity solvents (from nonpolar hexane to polar DMF)[9]. Δf = {f (ϵ) – f (η²)}

Lipper-Mataga Equation:

$$\bar{\nu}_{abs} - \bar{\nu}_{em} = \frac{2}{hca^3} \cdot \Delta f \cdot (\mu_E - \mu_G)^2 + constant$$

From the slope we have calculated the difference between excited state and ground state dipole moments (μ_E-μ_G) which correlates with the Stokes shift.

<u>**Slope**</u> $= \frac{2}{hca^3} \cdot (\mu_E - \mu_G)^2$

where h = planck's constant, c = velocity of light, a = radius of individual molecule (calculated from the volume of individual crystals).

Solvent-dependent prompt lifetimes:

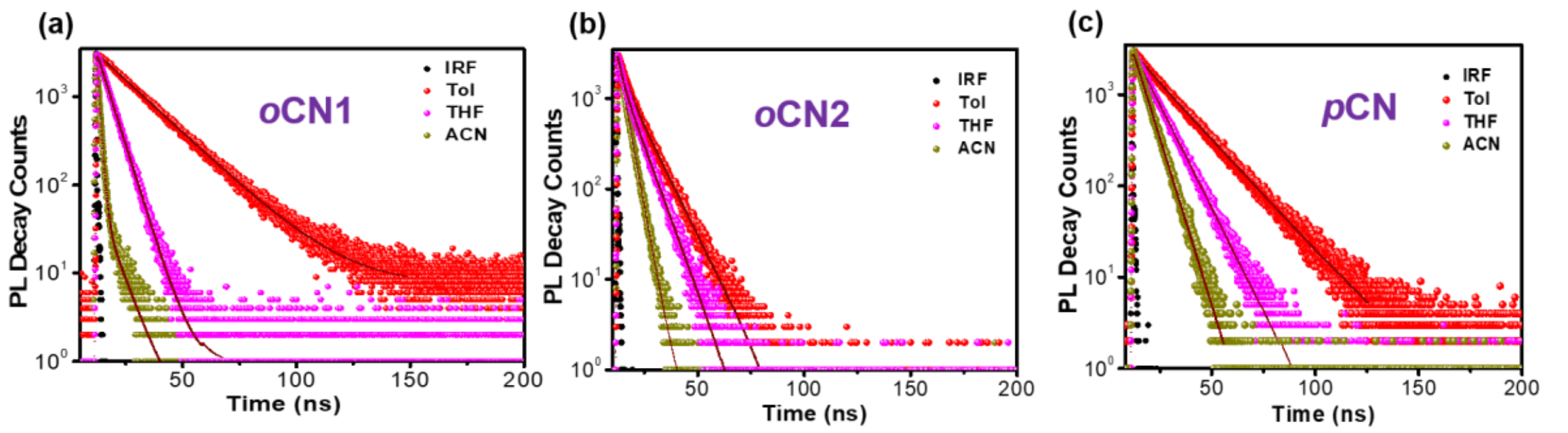

Fig. S5 Time-resolved fluorescence decay profiles (prompt lifetimes) of (a) ***o*CN1**, (b) ***o*CN2**, and (c) ***p*CN** in solvents of varying polarity (concentration 30 µm).

Table S1 Time-resolved fluorescence decay components (prompt lifetimes) in different solvents of ***o*CN1**, ***o*CN2**, ***p*CN** (concentration 30 µm).

**Solvent system**	***o*CN1**	***o*CN2**	***p*CN**
**Toluene**	18 ns	6.5 ns	12 ns
**THF**	5.1 ns	4.5 ns	9.6 ns
**ACN**	2.9 ns	2.74 ns	7.9 ns

Steady State and Gated spectra at RT in solution:

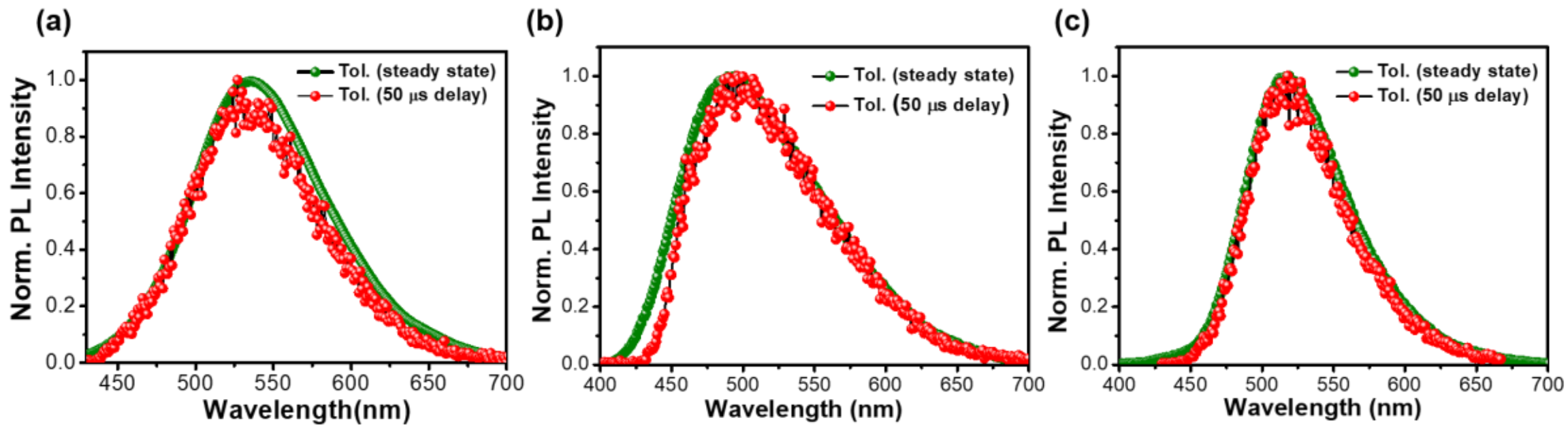

Fig. S6 Steady-state and time-gated (50 µs delay, 1 ms sample window) at RT emission spectra in degassed toluene of (a) ***o*CN1**, (b) ***o*CN2**, and (c) ***p*CN** (concentration 30 µm).

Temperature-dependent gated spectra in solution:

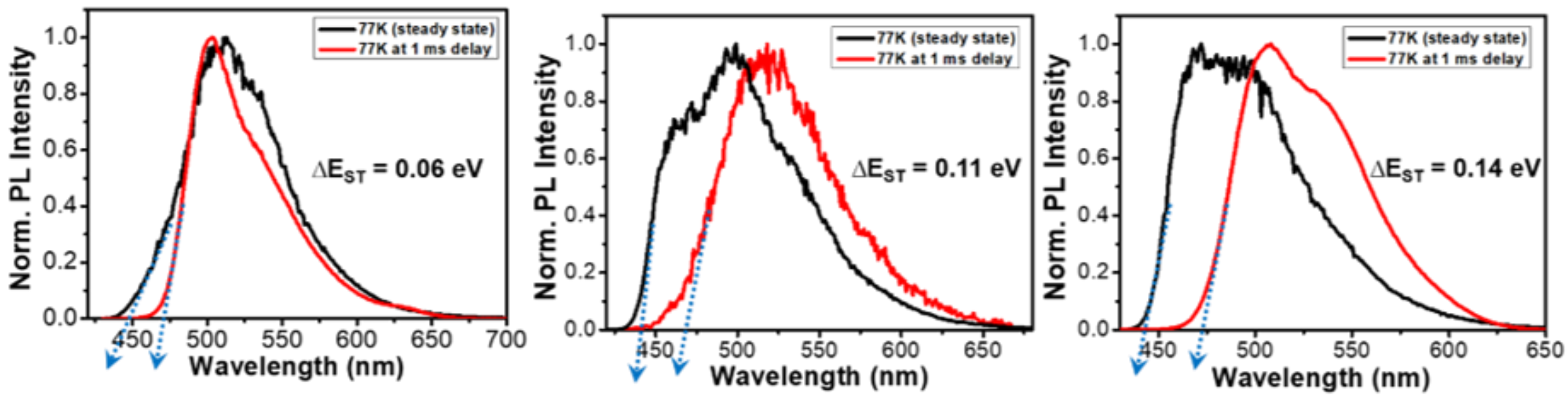

Fig. S7 Steady-state and time-gated emission spectra of (a) ***o*CN1**, (b) ***o*CN2** and (c) ***p*CN** (concentration 30 µM) at 77 K in 2-methyl-THF (glass freezing solvent). At 77 K (low-temperature), 1 ms delay time and 10 ms sample windows have been used.

NTOs Calculation for molecule in toluene solvent:

***o*CN1**

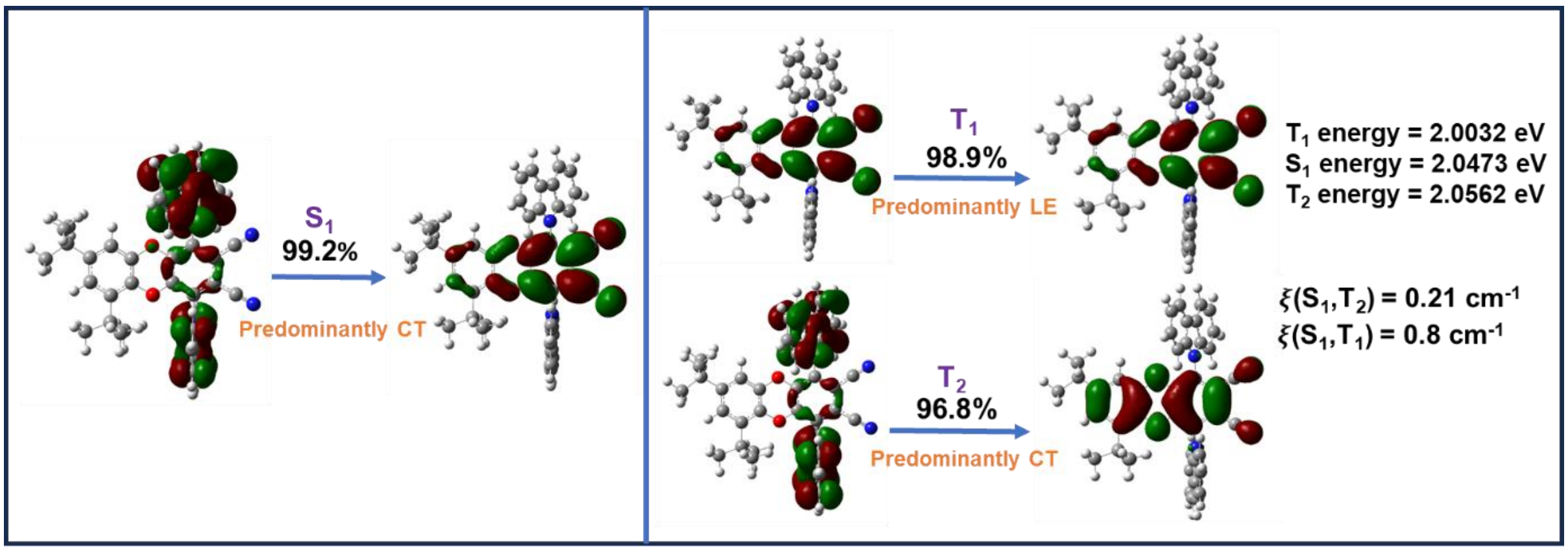

***o*CN2**

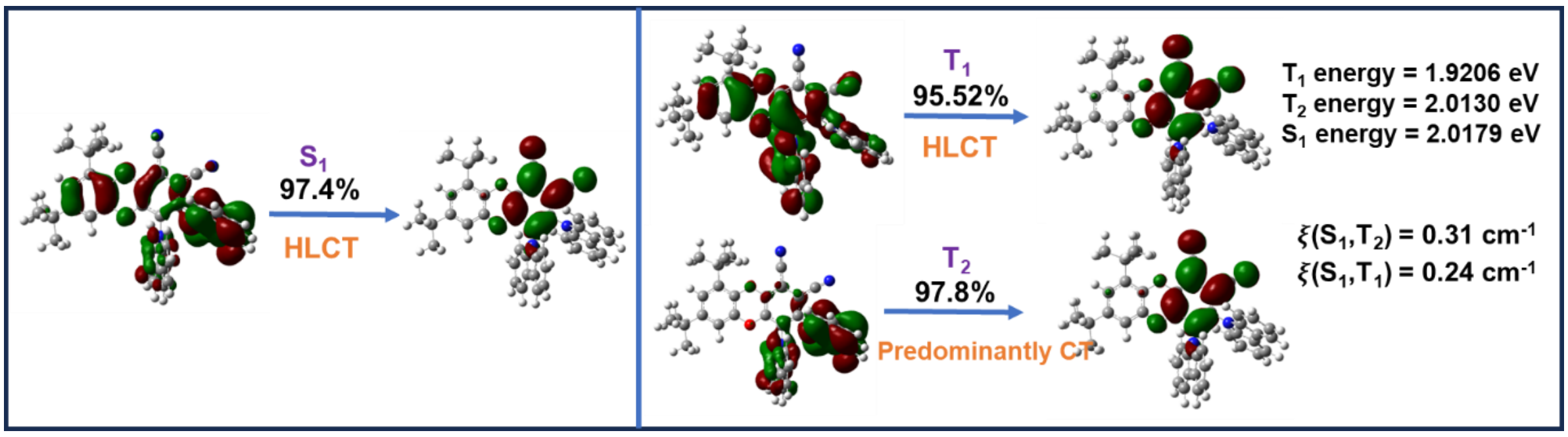

***p*CN**

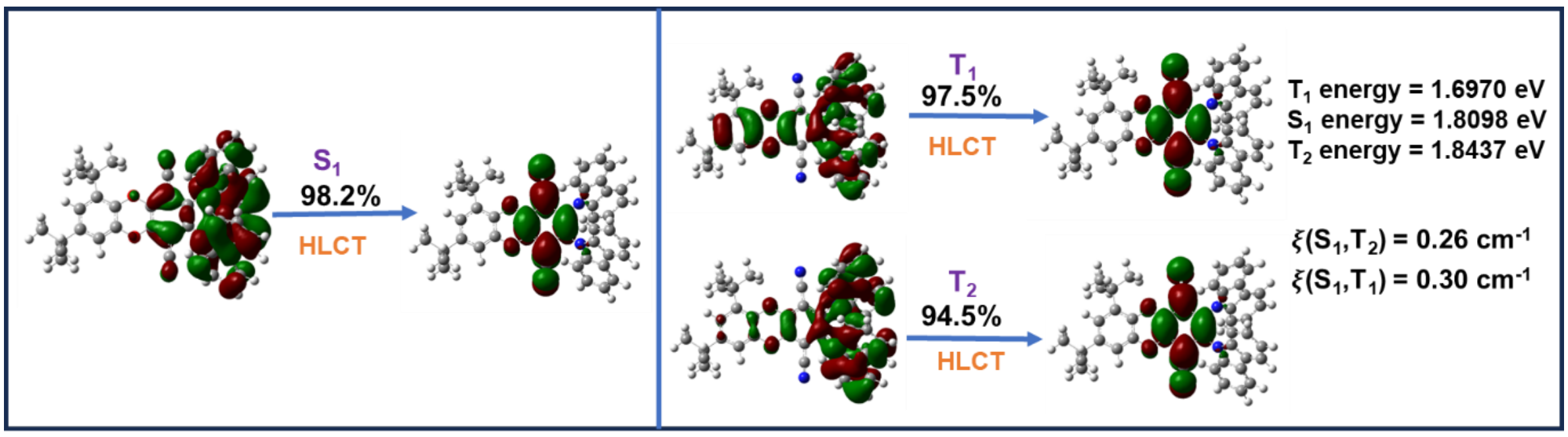

Fig. S8 Natural transition orbital (NTO) analysis, energies, and spin-orbit coupling calculations of all three emitters' S_1, T_1 and T_2 states in the solvent-based (IEFPCM/Toluene) model at PBE0/ 6-31G (d,p) level of theory.

Prompt and Delayed lifetimes in THF solvent only (0% water content):

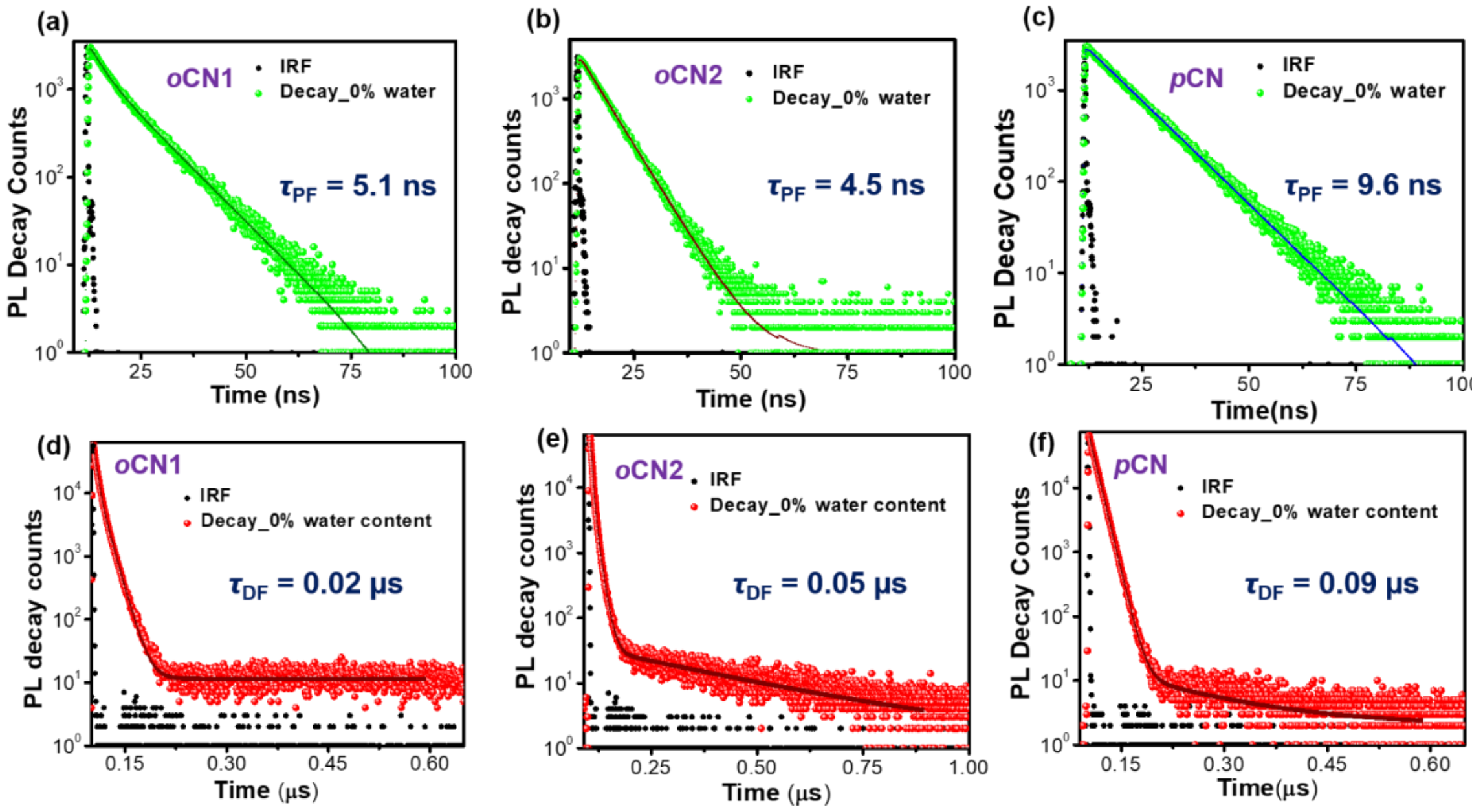

Fig. S9 Time-resolved fluorescence decay profiles of prompt and delayed lifetimes for (a), (d) ***o*CN1**, (b), (e) ***o*CN2**, and (c), (f) ***p*CN** in 0% water content in THF solution (concentration 30 μm).

DLS study of monomers and aggregates:

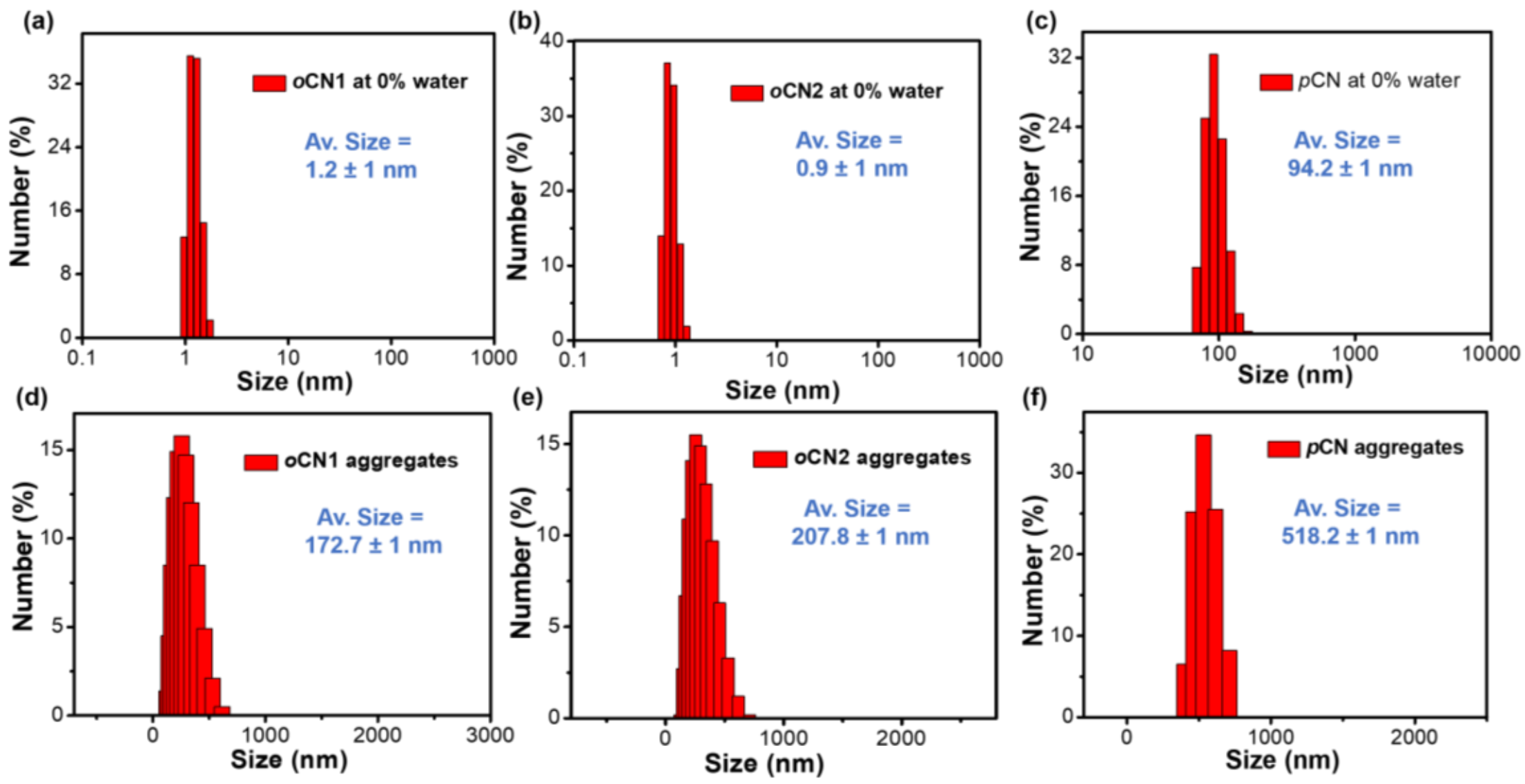

Fig. S10 DLS studies at 0% water content of (a) ***o*CN1**, (b) ***o*CN2** and (c) ***p*CN** and at higher water content of (d) ***o*CN1** (95% water) (e) ***o*CN2** (95% water) and (f) ***p*CN** (95% water).

Relative PLQY calculation for aggregates:

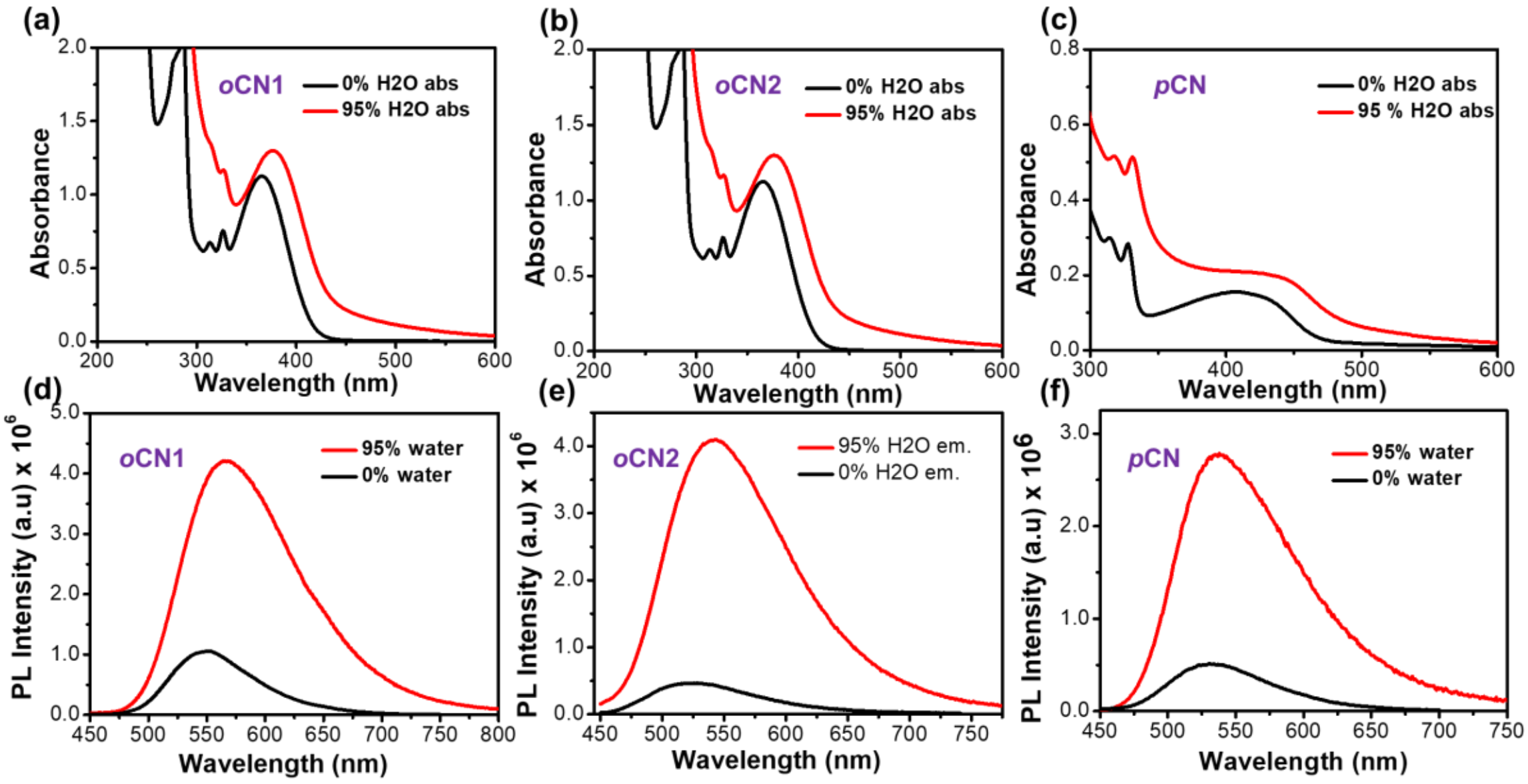

Fig. S11 Absorption and emission spectra of monomers and aggregates in THF-H2O binary mixture for (a), (d) ***o*CN1**, (b), (e) ***o*CN2**, (c), (f) ***p*CN** (30µM concentration). For 95% aggregates PLQY was calculated by using relative methods followed by:

PLQY of 95% aggregates = PLQY of 0% monomers × (area under the emission for 95% / area under the emission of 0%) × (absorbance of 0% / absorbance of 95%, at the absorption maxima of 0%).

PLQY of 0% monomers was measured by absolute PLQY method using integrating sphere.

Delayed lifetimes for aggregates in log-log scale:

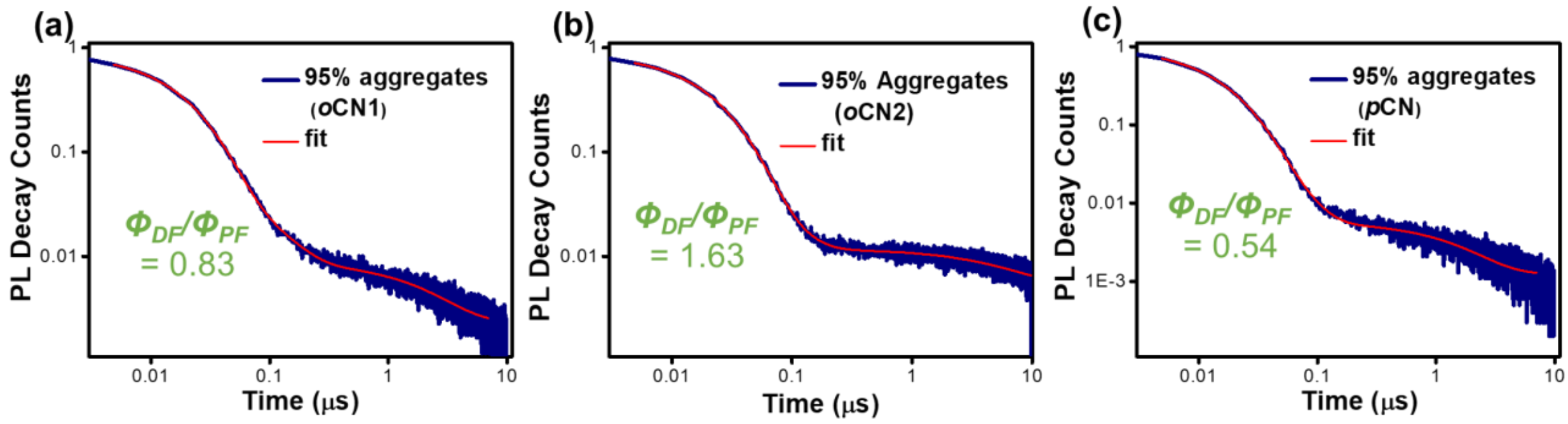

Fig. S12 TRPL data fitting with time-delay to quantify the delayed fluorescence (DF) and prompt fluorescence (PF) contribution ratio of 95% aggregates for all the emitters. For 95% aggregates, the pre-exponential factors (A_{PF} and A_{DF}) of the decay transients have been used for Φ_{DF}: Φ_{PF} ratio calculation using the standard formula Φ_{DF}: Φ_{PF}= ($A_{DF}\times \tau_d$)/ ($A_{PF}\times \tau_p$).

Delay-dependent Gated spectra at RT in neat film:

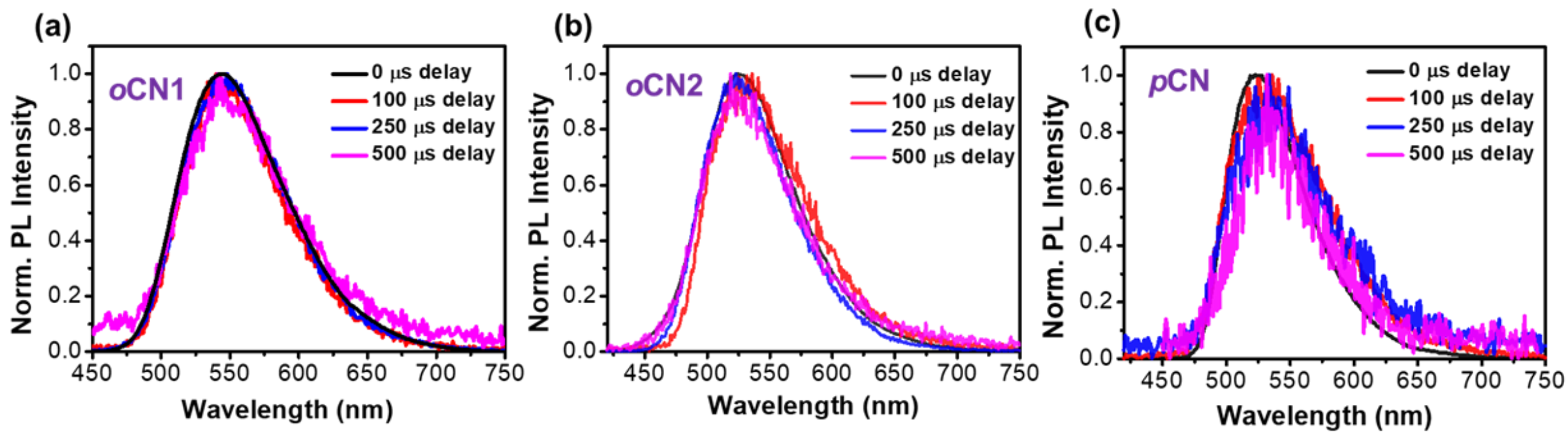

Fig. S13 Time-gated emission spectra with different time-delays for (a) ***o*CN1**, (b) ***o*CN2** and (c) ***p*CN** in neat films at RT.

Prompt Lifetimes and temperature-dependent delayed lifetimes in neat film:

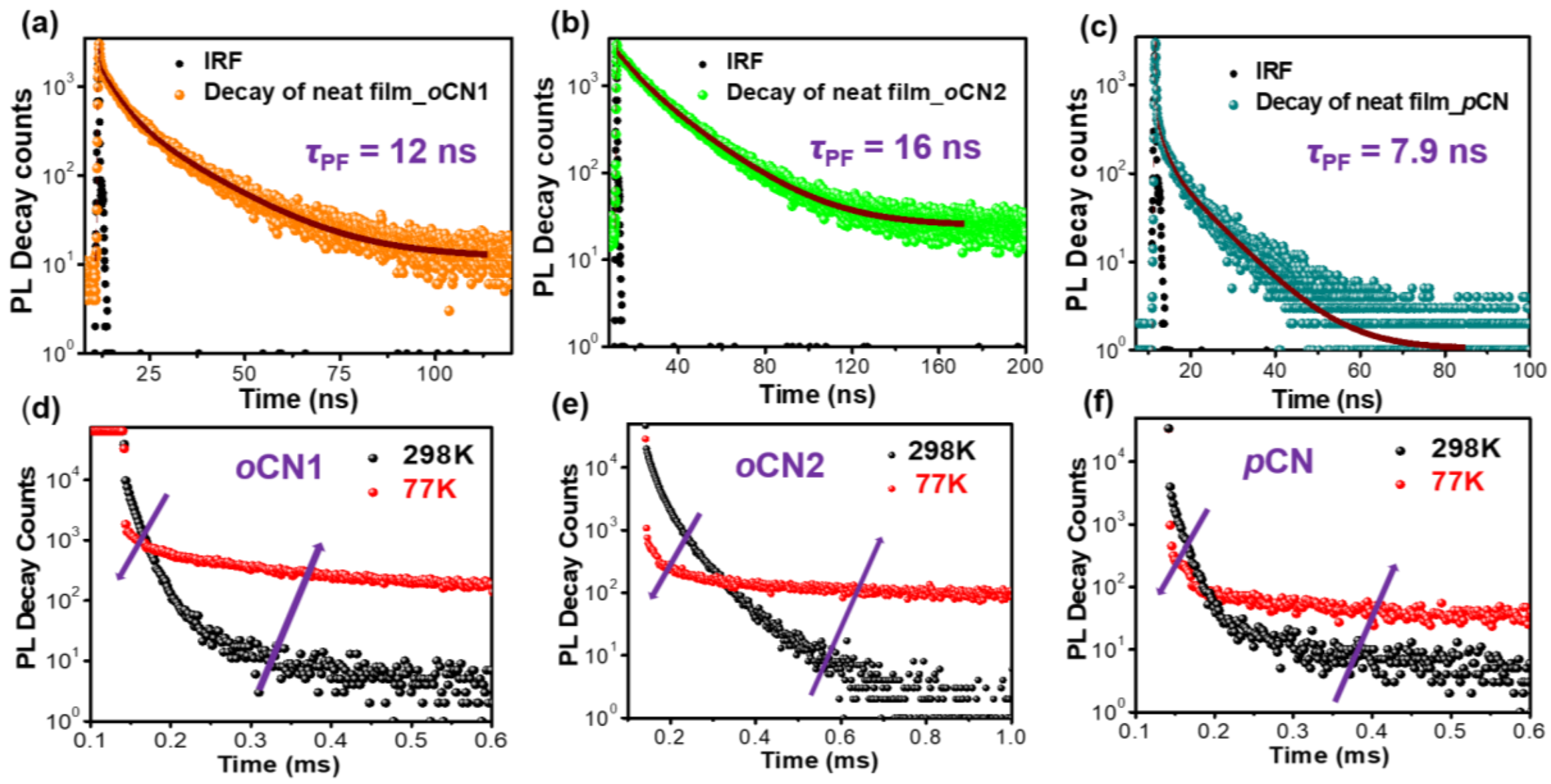

Fig. S14 Prompt fluorescence (PF) lifetime of all three luminogens in neat films of (a) ***o*CN1**, (b) ***o*CN2** and (c) ***p*CN** (excitation wavelength = 402 nm). Time-resolved emission decay profiles (λ_{exc} = 366nm) for the neat film of (d) ***o*CN1**, (e) ***o*CN2** and (f) ***p*CN** at RT (298 K) and 77 K.

Long Lifetimes at 77K in neat film:

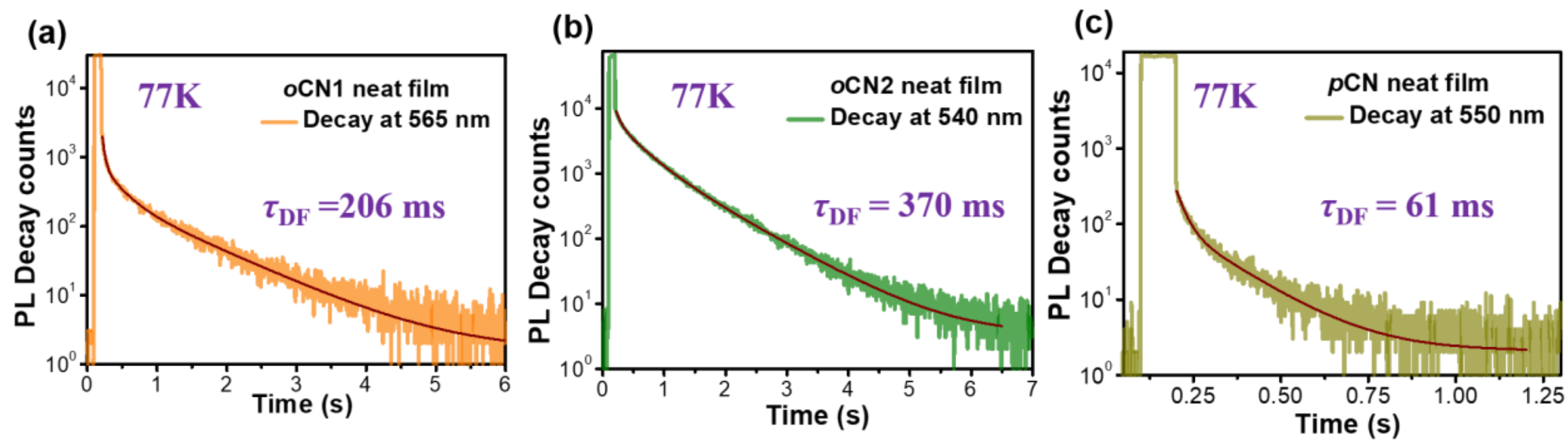

Fig. S15 Phosphorescence lifetime of (a) ***o*CN1**, (b) ***o*CN2** and (c) ***p*CN** in neat films at 77K.

NTOs calculation for aggregated state:

***o*CN1**

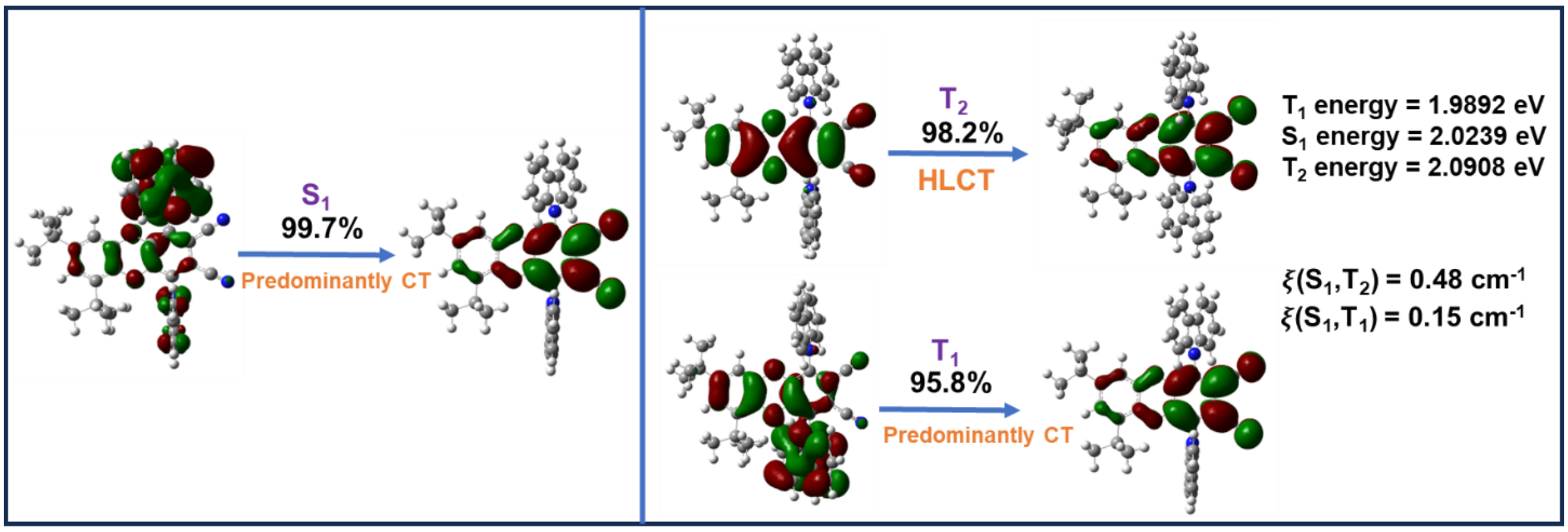

***o*CN2**

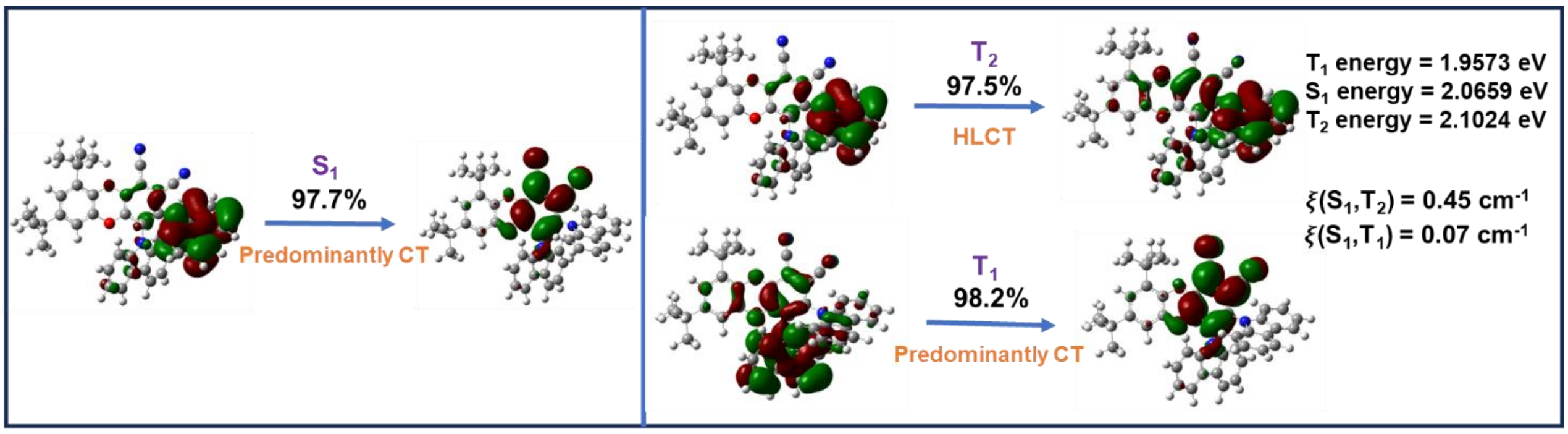

***p*CN**

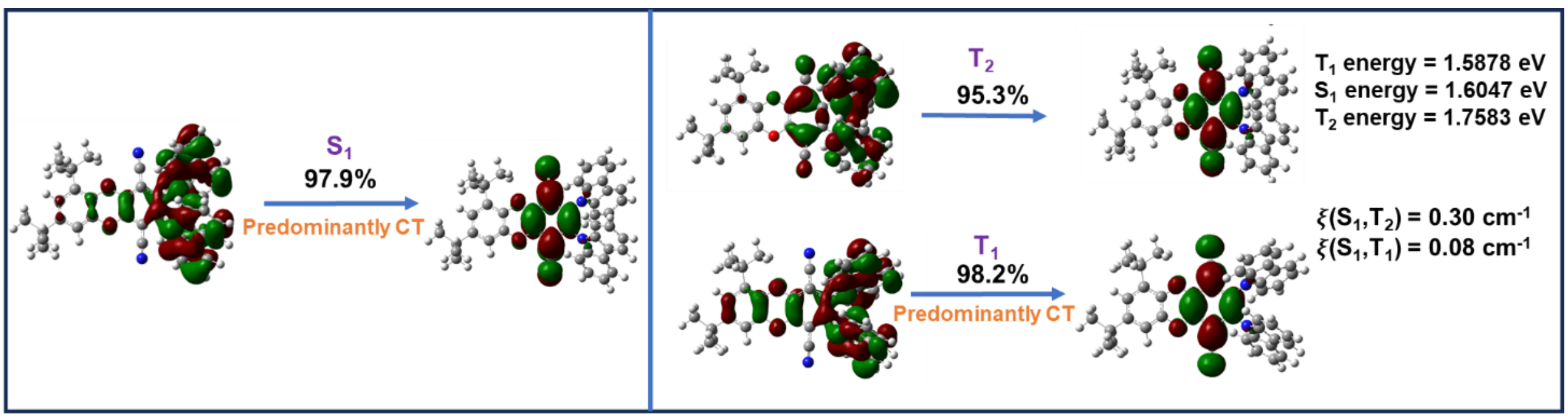

Fig. S16 Natural transition orbital (NTO) analysis for all the three emitters in aggregated state (obtained from QM/MM calculation). For the QM/MM calculation, the QM layer has been treated with PBE0, 6-31G (d, p) level of theory, while the MM layer has been treated with a classical UFF model of theory.

Table S2 Percentage of CT-LE characters for the excited states of aggregates (obtained from QM/MM calculation) using interfragmentary charge transfer method in Multiwfn software.[10]

Emitters	S_1		T_1		T_2	
	CT (%)	LE (%)	CT (%)	LE (%)	CT (%)	LE (%)
***o*CN1**	91.8	8.2	89.8	10.2	56.9	43.1
***o*CN2**	86.3	13.7	77.1	22.9	75.4	24.6
***p*CN**	92.8	7.1	92.4	7.6	89.7	10.3

Photoluminescence Emission and Lifetime for 10 wt% PMMA film:

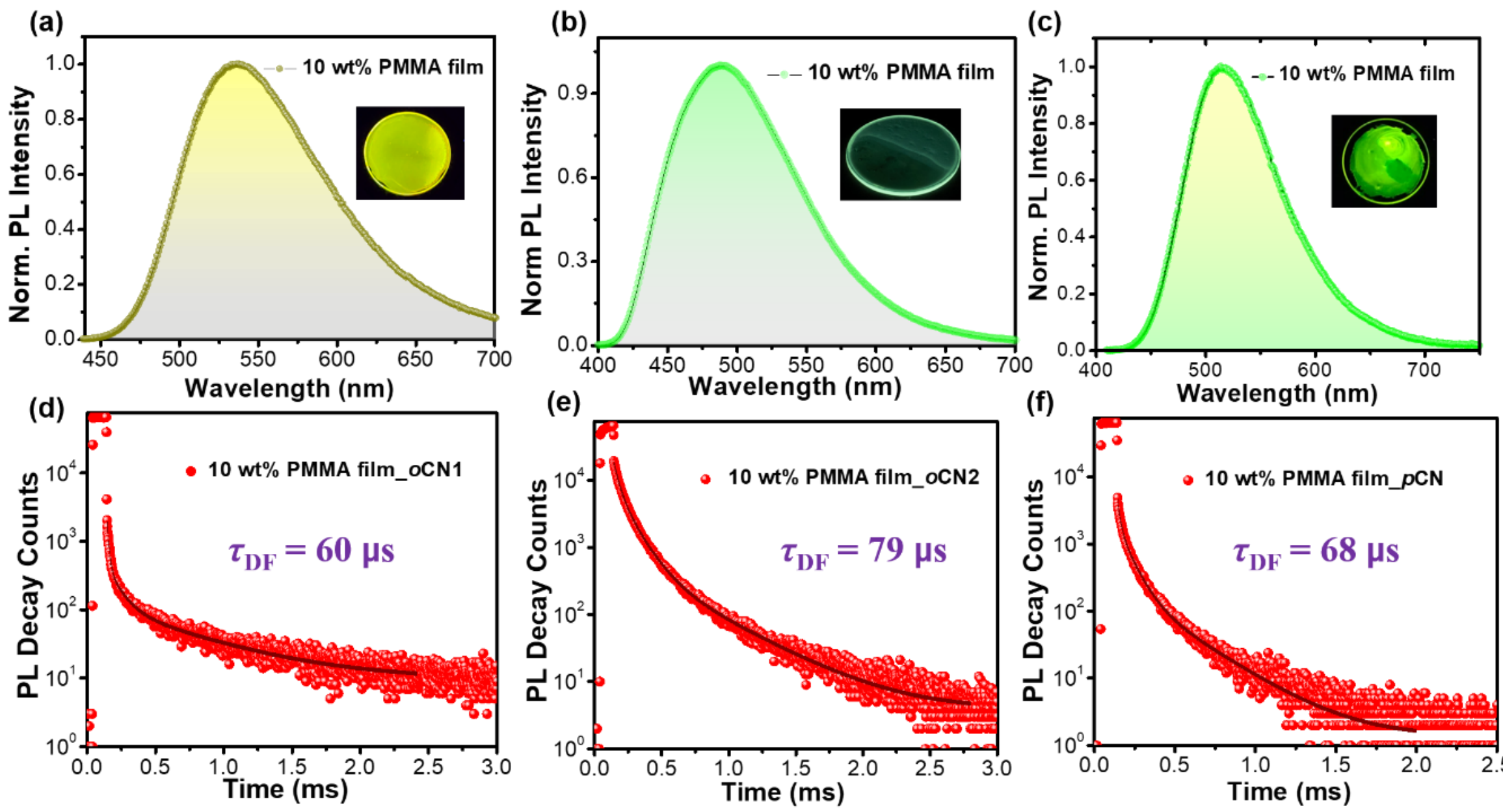

Fig. S17 Steady state and time-resolved photoluminescence spectra of (a), (d) ***o*CN1**, (b), (e) ***o*CN2** and (c), (f) ***p*CN** in 10 wt% PMMA films at RT.

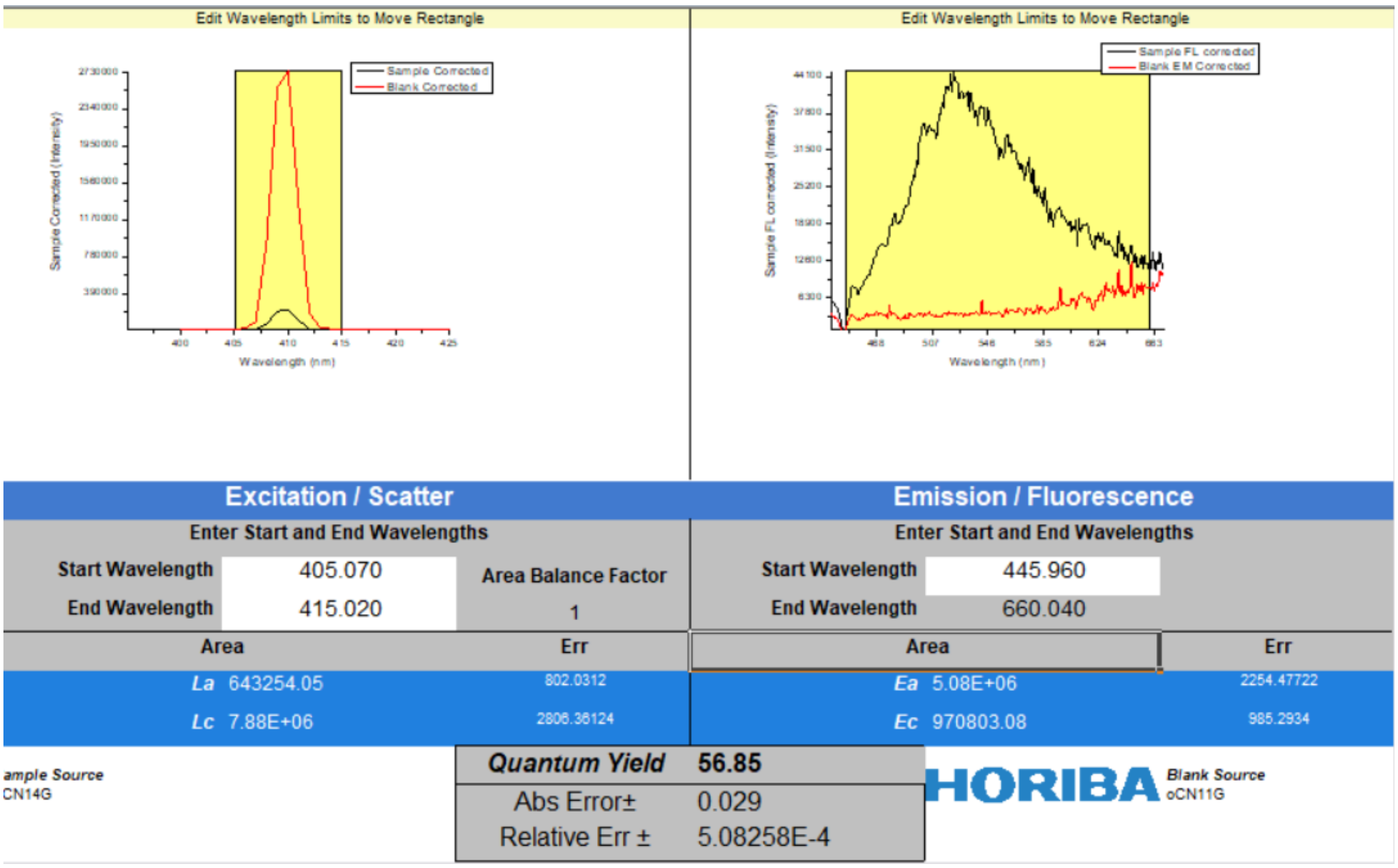

PLQY of *o*CN1 in toluene solution.

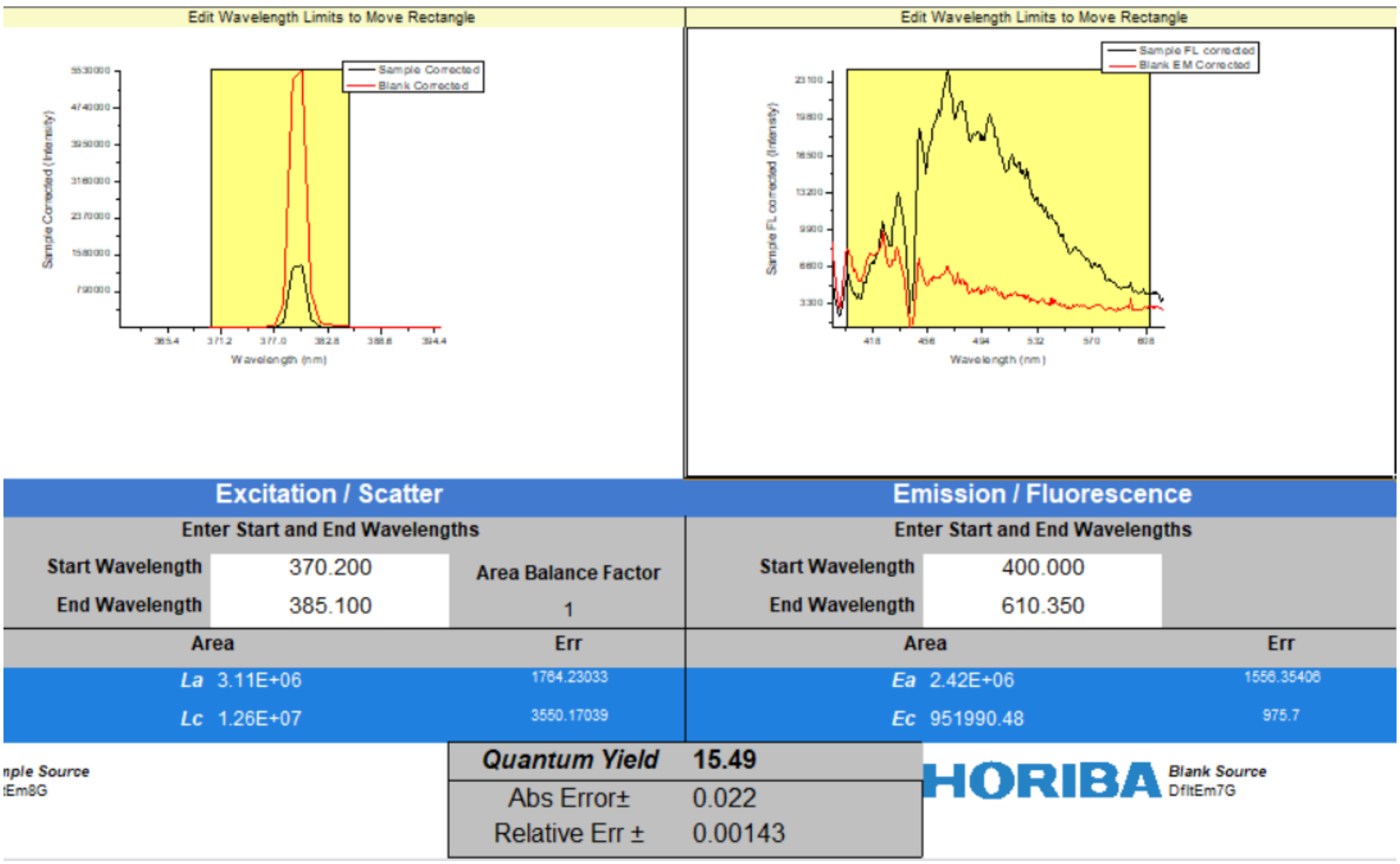

PLQY of *o*CN2 in toluene solution.

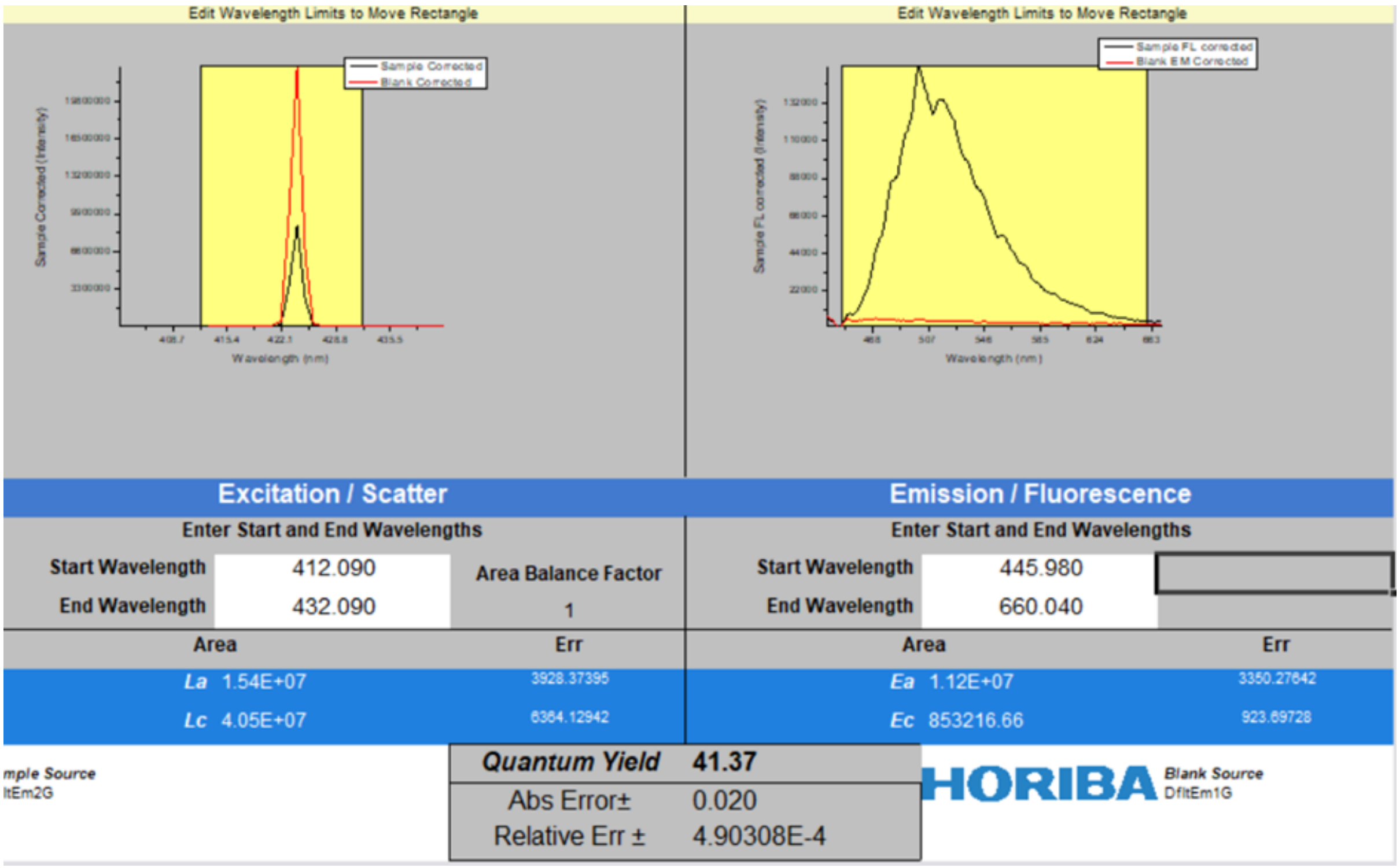

PLQY of *p*CN in toluene solution.

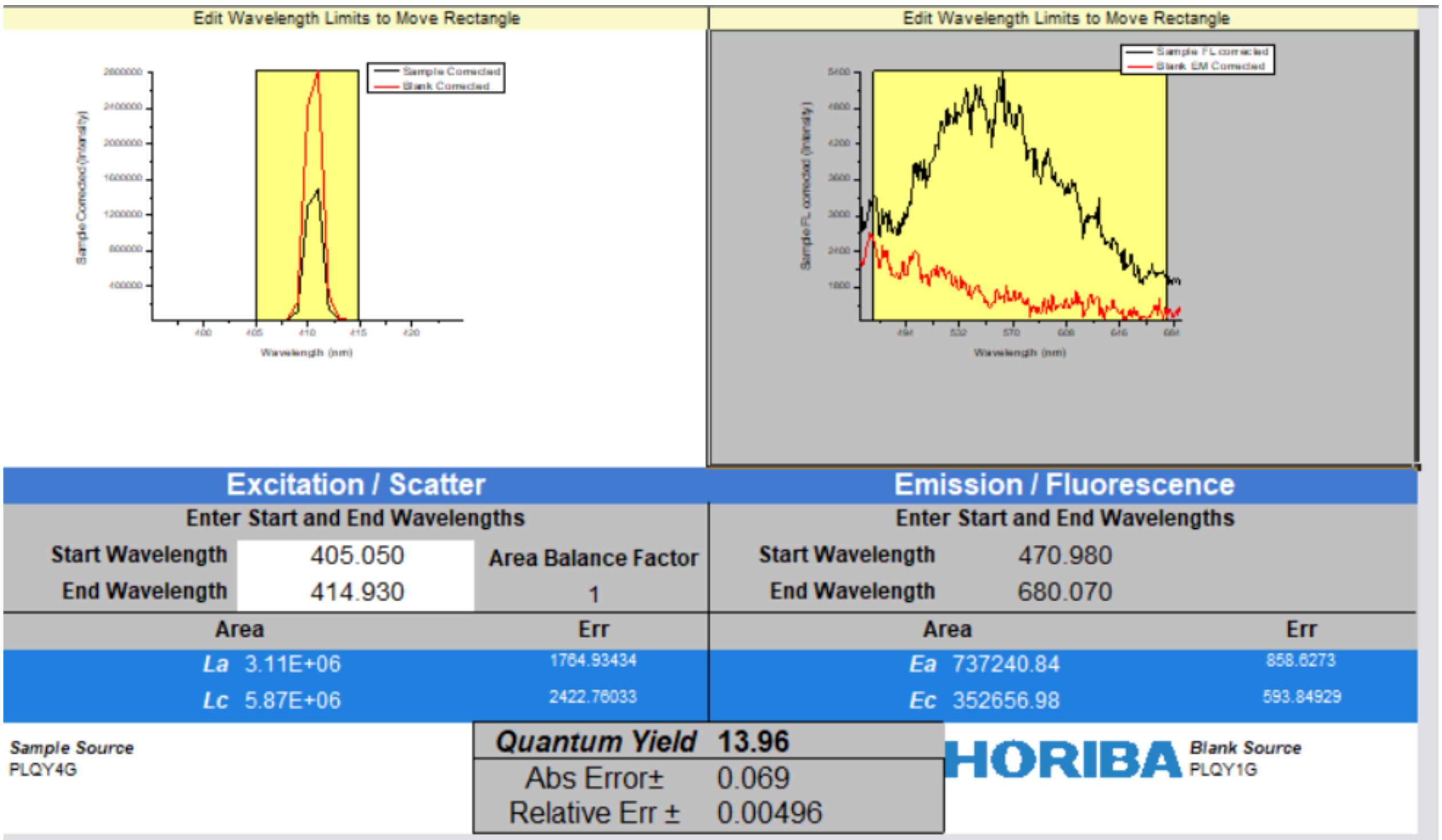

PLQY of *o*CN1 in 0% water content of THF solution.

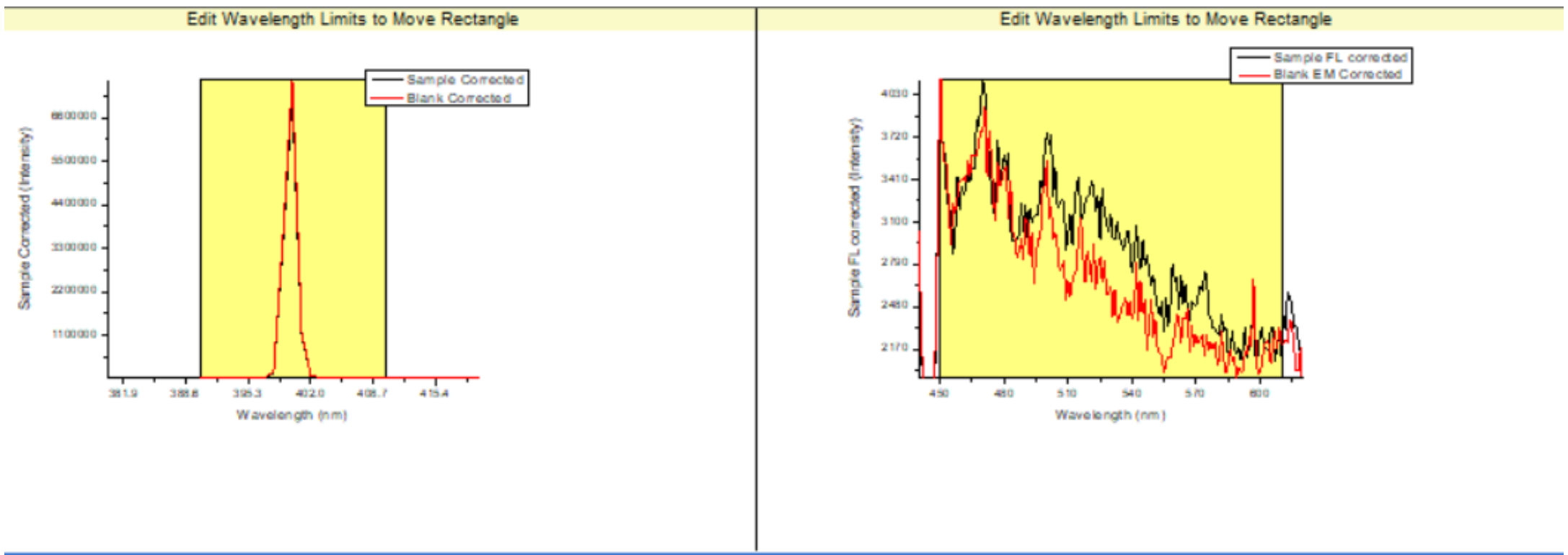

Excitation / Scatter			Emission / Fluorescence		
Enter Start and End Wavelengths			Enter Start and End Wavelengths		
Start Wavelength	390.160	Area Balance Factor	Start Wavelength	449.930	
End Wavelength	410.080	1	End Wavelength	610.230	
Area		Err	Area		Err
La	1.22E+07	3488.06327	*Ea*	466935.10	683.3265
Lc	1.27E+07	3566.37337	*Ec*	431015.00	656.51733

e Source 2G	**Quantum Yield**	**6.50**
	Abs Error±	0.645
	Relative Err ±	0.09922

HORIBA *Blank Source* DfltEm1G

PLQY of *o*CN2 in 0% water content of THF solution.

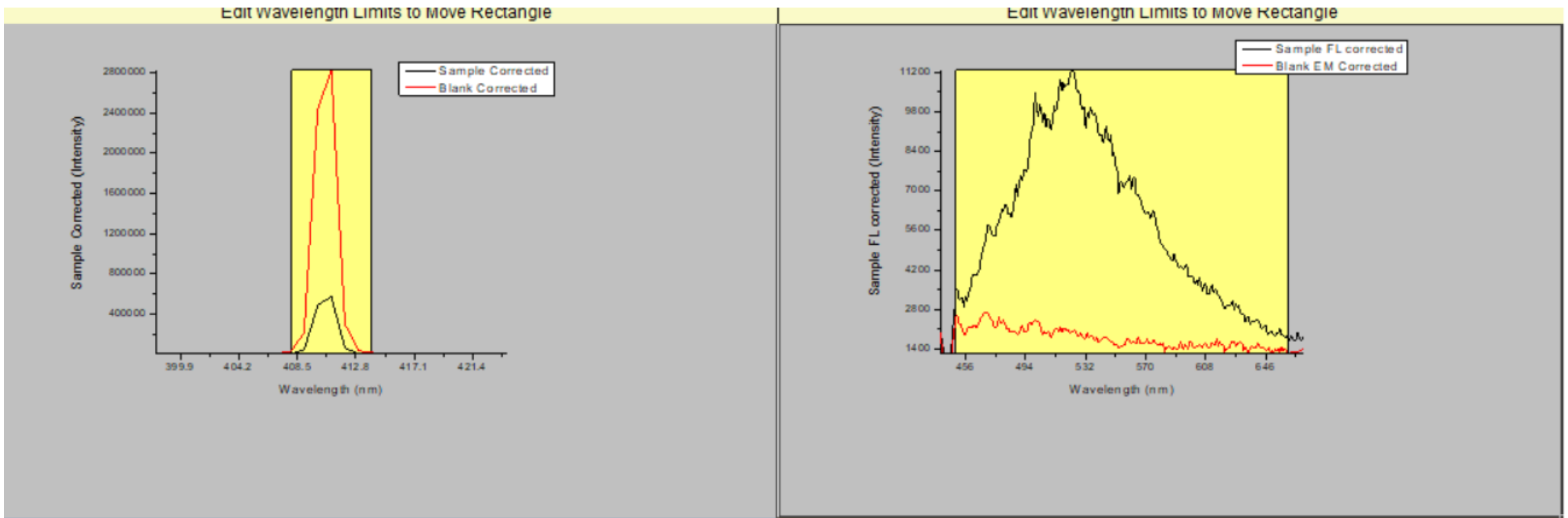

Excitation / Scatter			Emission / Fluorescence		
Enter Start and End Wavelengths			Enter Start and End Wavelengths		
Start Wavelength	408.070	Area Balance Factor	Start Wavelength	449.980	
End Wavelength	413.950	1	End Wavelength	660.070	
Area		Err	Area		Err
La	1.16E+06	1074.87466	*Ea*	1.23E+06	1109.83645
Lc	5.69E+06	2385.00647	*Ec*	374002.65	611.55756

Sample Source PLQY3G	**Quantum Yield**	**18.92**
	Abs Error±	0.029
	Relative Err ±	0.00155

HORIBA *Blank Source* PLQY1G

PLQY of *p*CN in 0% water content of THF solution

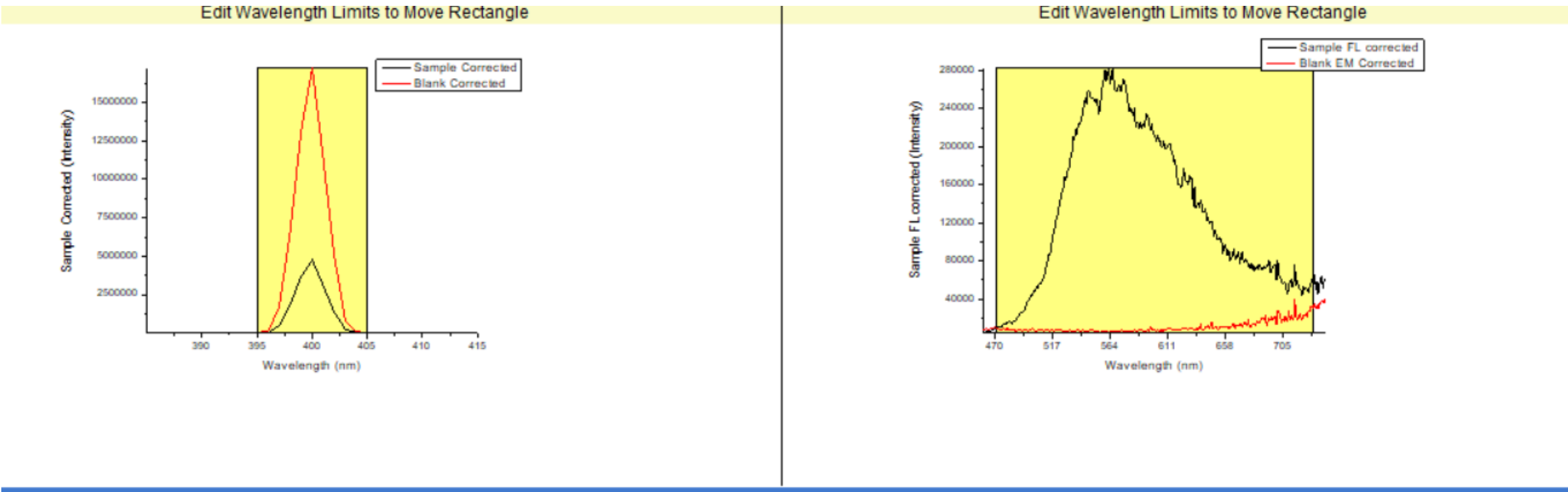

Excitation / Scatter			Emission / Fluorescence		
Enter Start and End Wavelengths			Enter Start and End Wavelengths		
Start Wavelength	395.020	Area Balance Factor	Start Wavelength	470.930	
End Wavelength	405.030	1	End Wavelength	730.020	
Area		Err	Area		Err
La	1.55E+07	3942.61685	*Ea*	3.45E+07	5873.63158
Lc	5.61E+07	7491.70033	*Ec*	2.73E+06	1651.70824

Quantum Yield	78.29
Abs Error±	0.019
Relative Err ±	2.48236E-4

le Source sa7G

HORIBA *Blank Source* oCN1sa4G

PLQY of *o*CN1 in neat film.

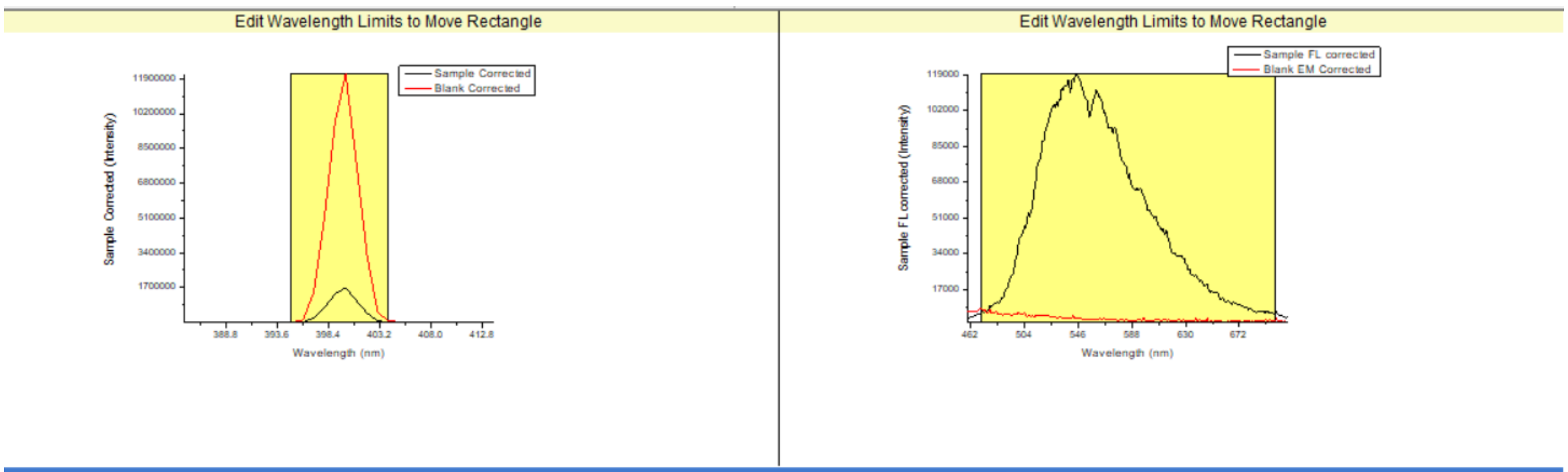

Excitation / Scatter			Emission / Fluorescence		
Enter Start and End Wavelengths			Enter Start and End Wavelengths		
Start Wavelength	394.930	Area Balance Factor	Start Wavelength	469.940	
End Wavelength	403.910	1	End Wavelength	700.020	
Area		Err	Area		Err
La	5.60E+06	2365.76722	*Ea*	1.15E+07	3394.04253
Lc	3.98E+07	6310.13299	*Ec*	757933.07	870.59351

Quantum Yield	31.45
Abs Error±	0.010
Relative Err ±	3.06026E-4

Sample Source oCN112G

HORIBA *Blank Source* blank3G

PLQY of *o*CN2 in neat film.

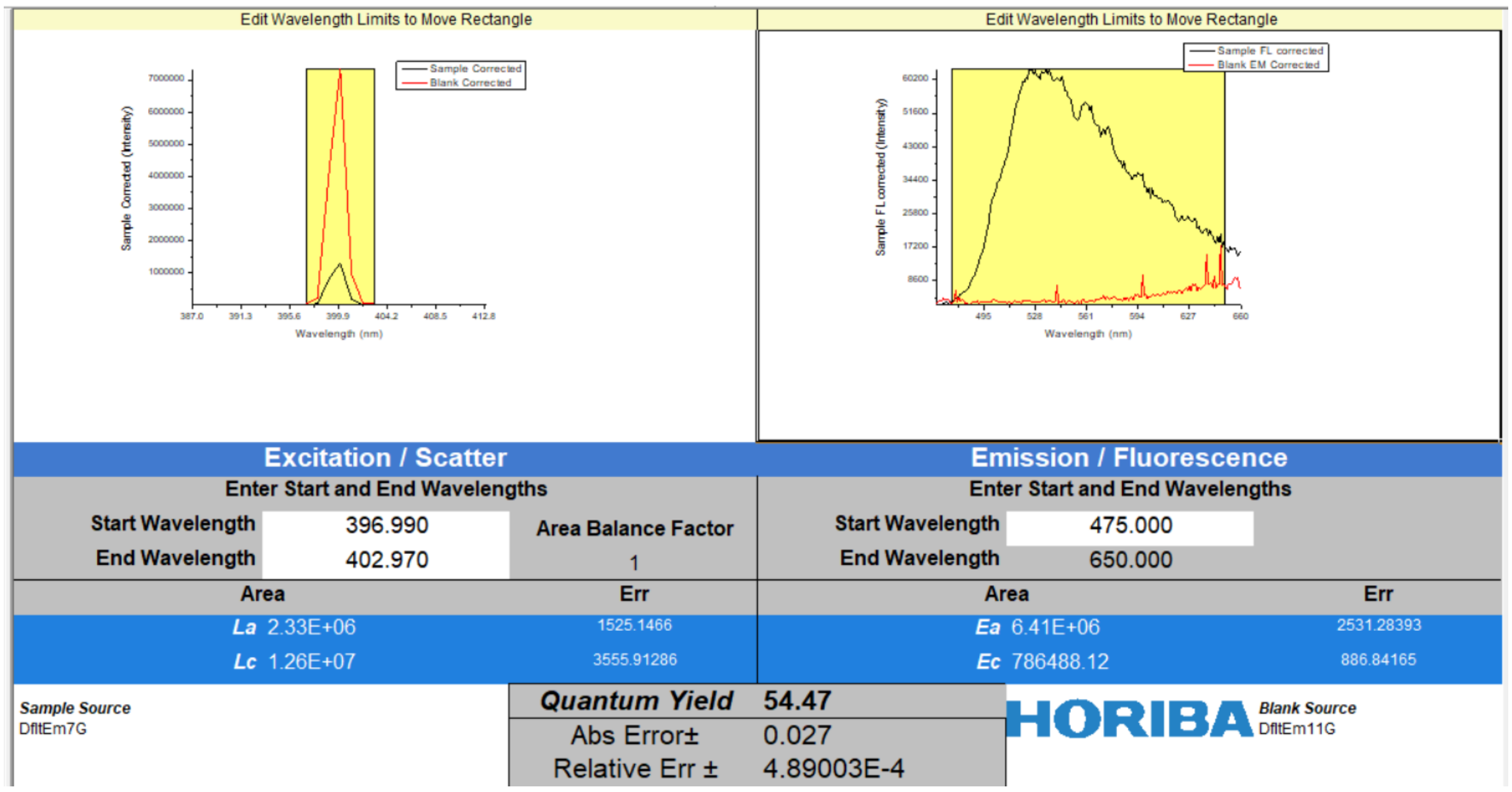

PLQY of *p*CN in neat film.

Section S7: Crystallographic Data

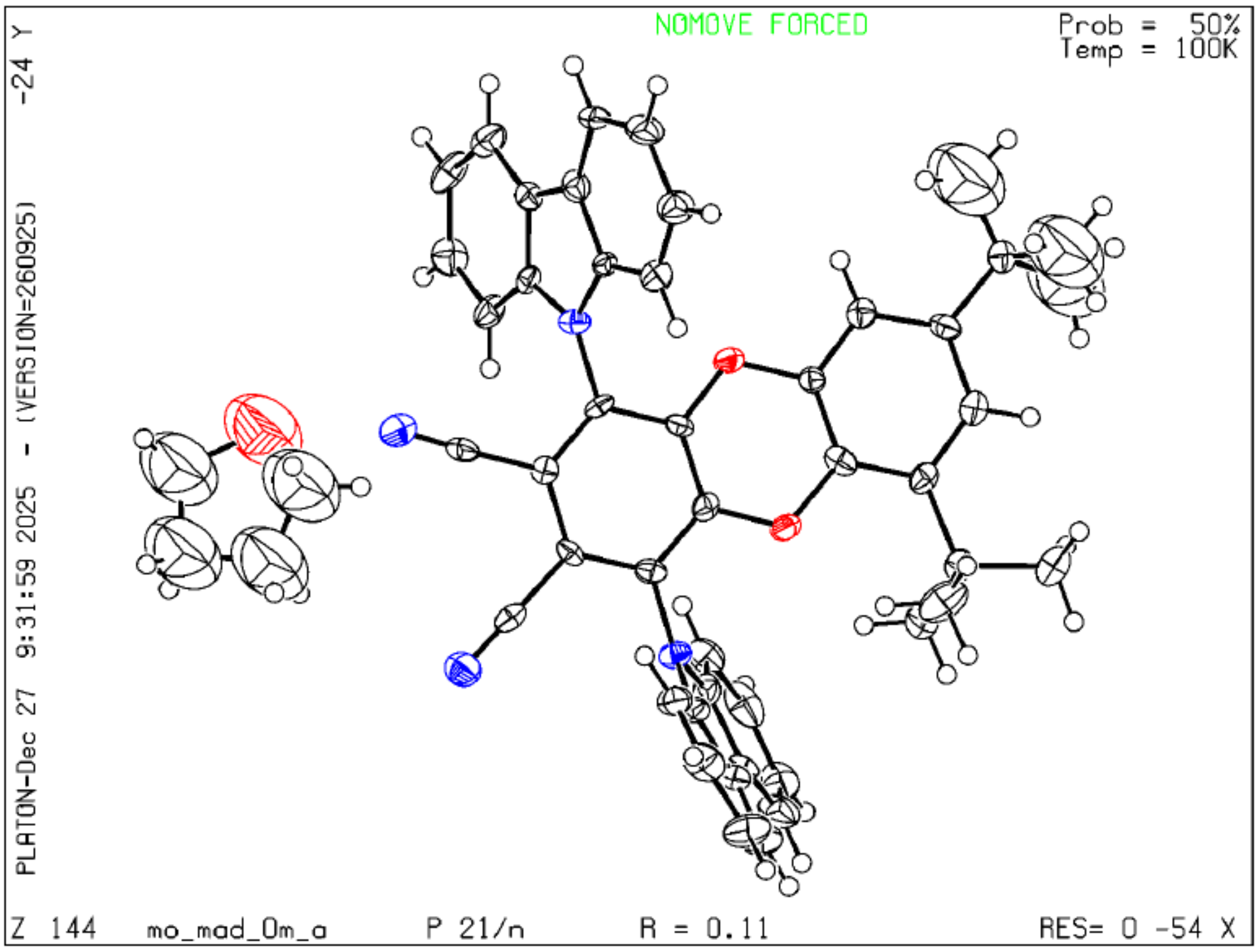

Table S3 Crystal data and structure refinement for ***o*CN1**.

**Identification code**	*o*CN1
**CCDC number**	2551023
**Empirical formula**	C50 H44 N4 O3
**Formula weight**	748.89
**Temperature/K**	100(2)
**Crystal system**	monoclinic
**Space group**	$P2_1/n$
**a/Å**	20.821(14)
**b/Å**	9.344(6)
**c/Å**	21.315(14)
**α/°**	90
**β/°**	104.810(15)

**γ/°**	90
**Volume/Å3**	4009(4)
**Z**	4
**ρ_{calc}(g/cm^3)**	1.241
**μ/mm^{-1}**	0.078
**F(000)**	236.0
**Crystal size/mm^3**	0.18 × 0.01 × 0.008
**Radiation**	Mo Kα (λ = 0.71073)
**2Θ range for data collection/°**	2.44 to 44.998
**Index ranges**	-22 ≤ h ≤ 22, -10 ≤ k ≤ 10, -22 ≤ l ≤ 22
**Reflections collected**	24537
**Independent reflections**	5211 [Rint = 0.1584, Rsigma = 0.1388]
**Data/restraints/parameters**	5211/70/472
**Goodness-of-fit on F2**	1.501
**Final R indexes [I>=2σ (I)]**	R1 = 0.1115, wR2 = 0.2695
**Final R indexes [all data]**	R1 = 0.2021, wR2 = 0.3190
**Largest diff. peak/hole / e Å^{-3}**	1.59/-0.67

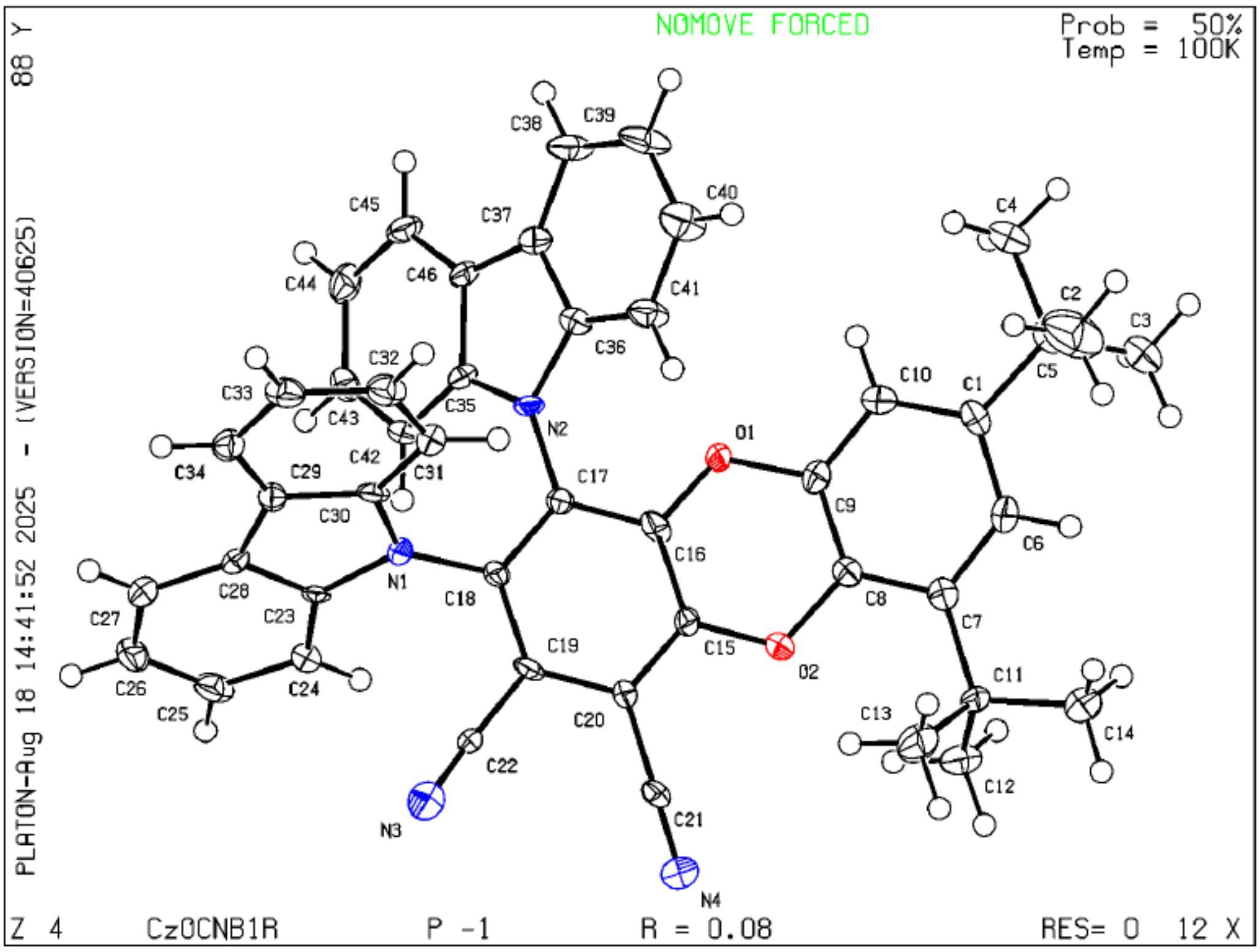

Table S4 Crystal data and structure refinement for ***o*****CN2**.

**Identification code**	*o*CN2
**CCDC number**	2551024
**Empirical formula**	C46 H36 N4 O2
**Formula weight**	676.79
**Temperature/K**	100(2)
**Crystal system**	triclinic
**Space group**	P-1
**a/Å**	10.287(4)
**b/Å**	11.229(4)
**c/Å**	16.180(6)
**α/°**	80.731(11)
**β/°**	85.950(10)

**γ/°**	75.067(11)
**Volume/Å^3**	1781.3(11)
**Z**	2
**ρ_{calc}(g/cm^3)**	1.262
**μ/mm-1**	0.078
**F(000)**	712.0
**Crystal size/mm^3**	0.15 × 0.04 × 0.03
**Radiation**	MoKα (λ = 0.71073)
**2Θ range for data collection/°**	3.796 to 42.996
**Index ranges**	-10 ≤ h ≤ 10, -11 ≤ k ≤ 11, -16 ≤ l ≤ 16
**Reflections collected**	45179
**Independent reflections**	4103 [Rint = 0.1239, Rsigma = 0.0630]
**Data/restraints/parameters**	4103/0/475
**Goodness-of-fit on F2**	1.451
**Final R indexes [I>=2σ (I)]**	R1 = 0.0839, wR2 = 0.2077
**Final R indexes [all data]**	R1 = 0.1251, wR2 = 0.2281
**Largest diff. peak/hole / e Å^{-3}**	0.99/-0.30

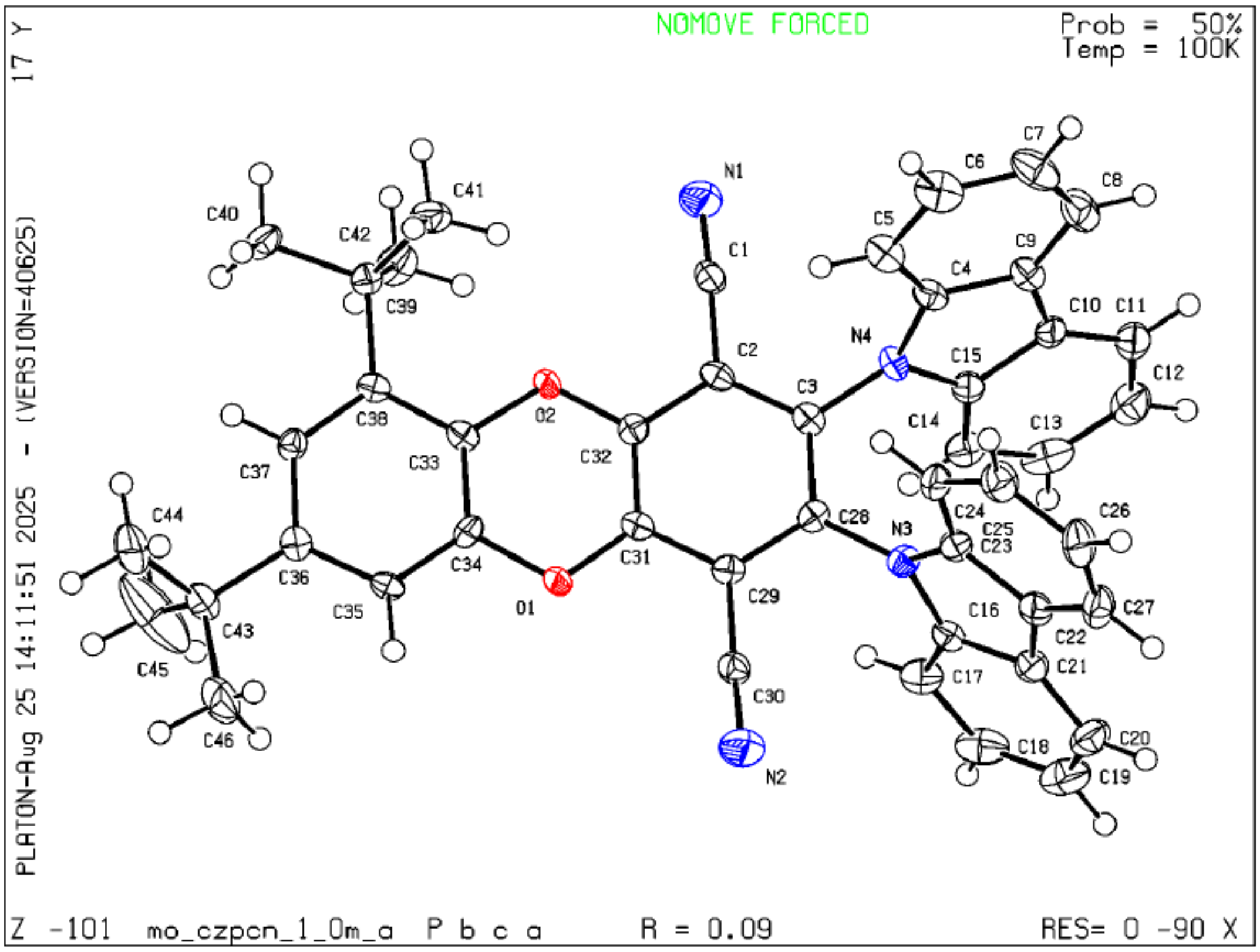

Table S5 Crystal data and structure refinement for ***p*CN**.

**Identification code**	*p*CN
**CCDC number**	2551025
**Empirical formula**	C46 H36 N4 O2,
**Formula weight**	676.79
**Temperature/K**	100(2)
**Crystal system**	orthorhombic
**Space group**	Pbca
**a/Å**	15.493(2)
**b/Å**	11.373(2)
**c/Å**	41.318(8)
**α/°**	90
**β/°**	90

**γ/°**	90
**Volume/Å^3**	7280(2)
**Z**	8
**ρ_{calc}(g/cm^3)**	1.235
**μ/mm^{-1}**	0.076
**F(000)**	2848.0
**Crystal size/mm^3**	0.2 × 0.06 × 0.03
**Radiation**	MoKα (λ = 0.71073)
**2Θ range for data collection/°**	4.55 to 48.998
**Index ranges**	-18 ≤ h ≤ 16, -13 ≤ k ≤ 13, -48 ≤ l ≤ 48
**Reflections collected**	170751
**Independent reflections**	6057 [Rint = 0.2483, Rsigma = 0.0723]
**Data/restraints/parameters**	6057/7/475
**Goodness-of-fit on F2**	0.887
**Final R indexes [I>=2σ (I)]**	R1 = 0.0899, wR2 = 0.2098
**Final R indexes [all data]**	R1 = 0.1457, wR2 = 0.2546
**Largest diff. peak/hole / e Å^{-3}**	0.56/-0.43

Unit cell packing along a,b and c-axes:

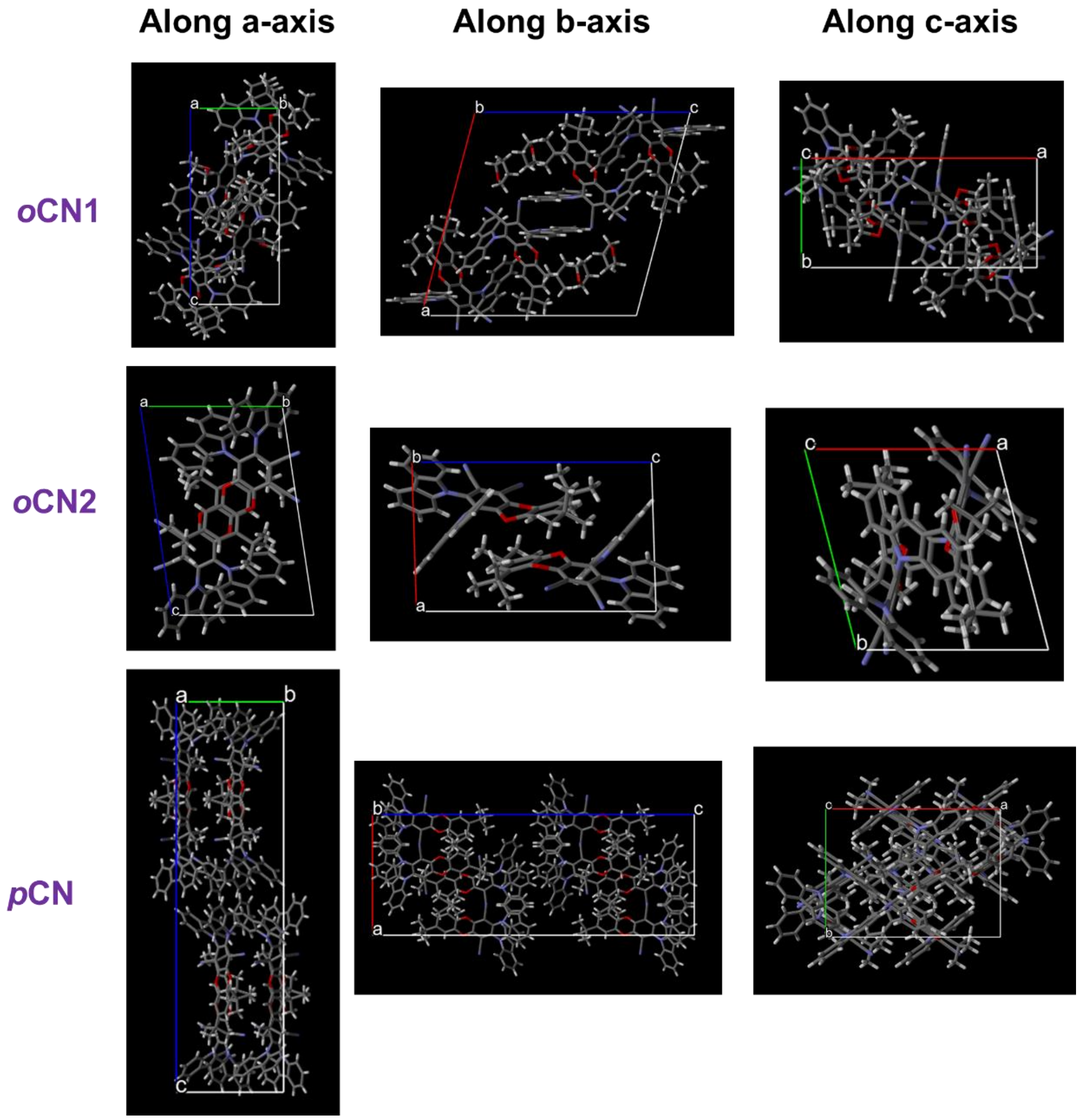

Fig. S18 Molecular packing along a, b and c crystallographic axis.

Angle between planes of peripheral benzene rings in acceptor core:

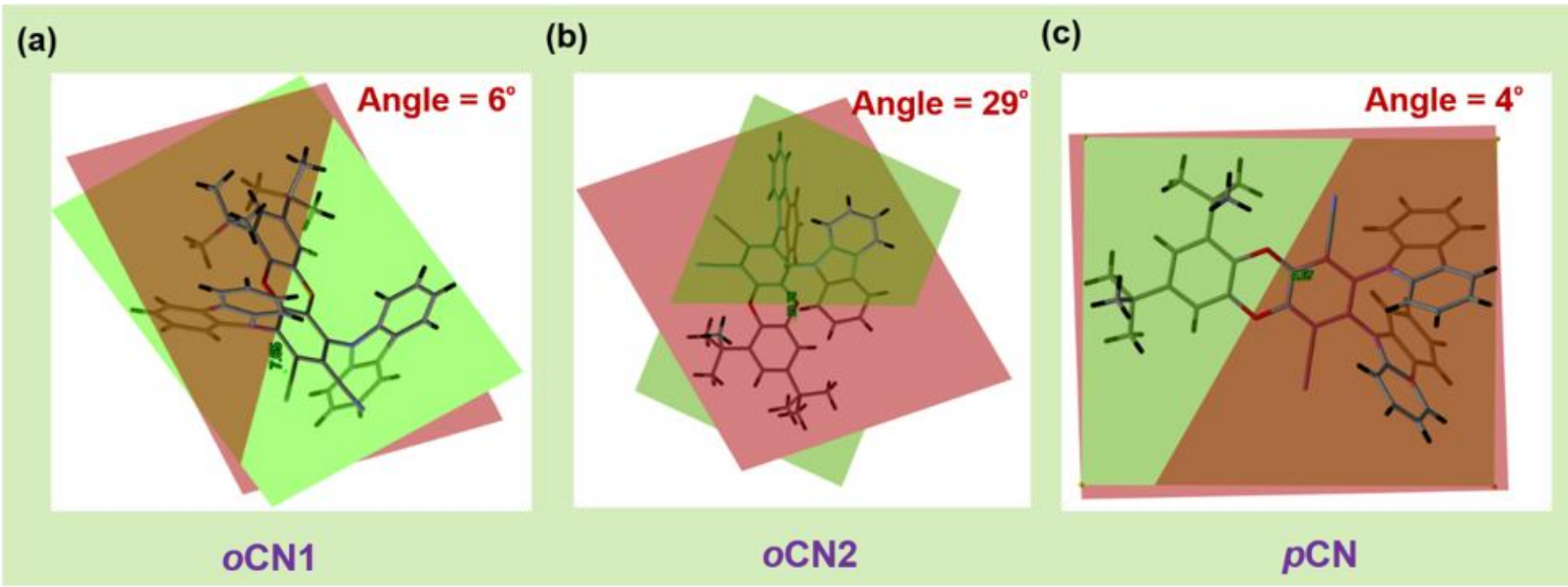

Fig. S19 Angles between two peripheral benzene rings in dioxin acceptor core for (a) ***o*CN1**, (b) ***o*CN2** and (c) ***p*CN**.

Intermolecular distances between two molecules:

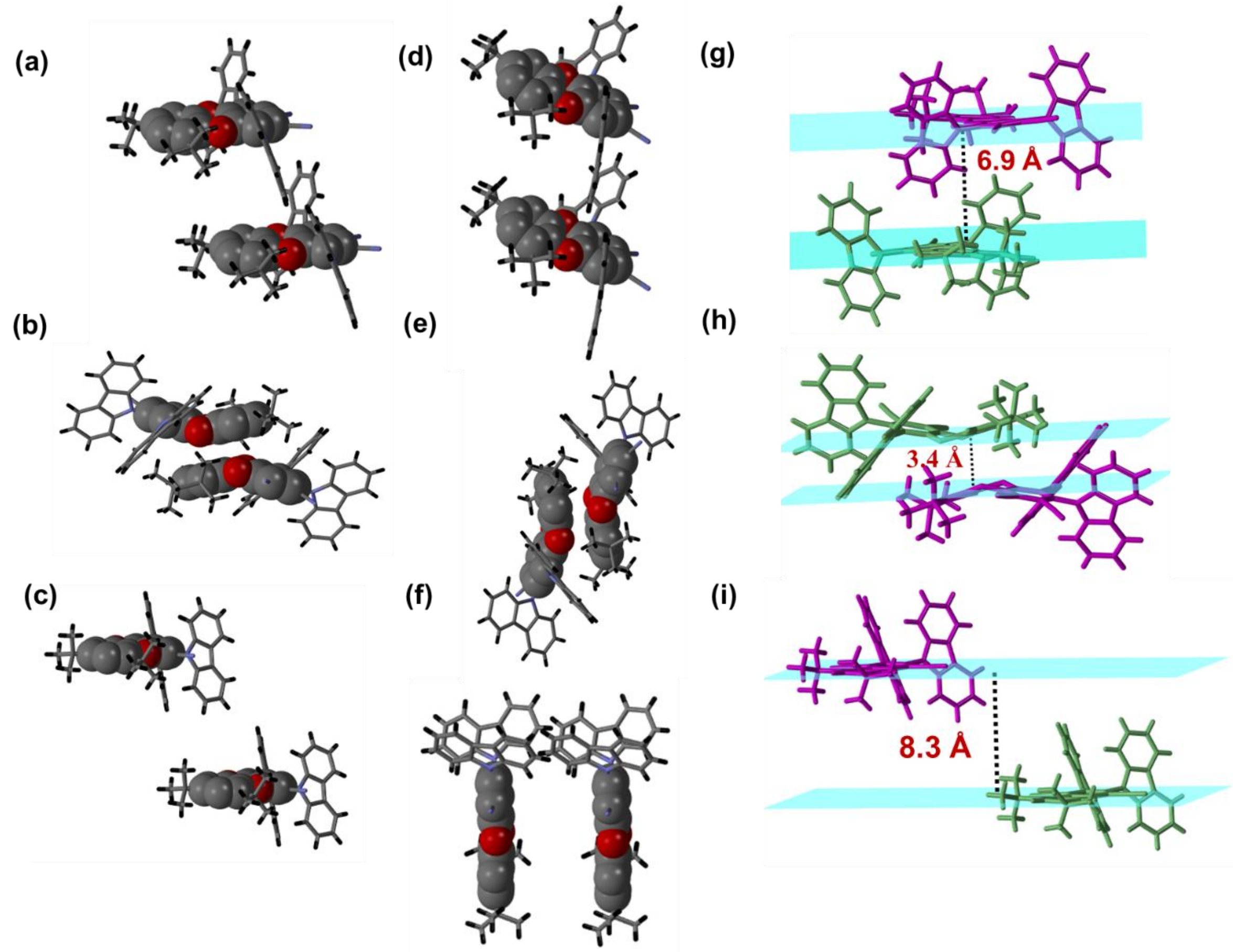

Fig. S20 Space-filling model of (a) ***o*CN1**, (b) ***o*CN2** and (c) ***p*CN** from side-view. Space-filling model of (d) ***o*CN1**, (e) ***o*CN2** and (f) ***p*CN** from top-view. π···π stacking interactions distance of two neighboring molecules of (g) ***o*CN1**, (h) ***o*CN2** and (i) ***p*CN**.

Intra and Intermolecular centroid-centroid distance between carbazole rings:

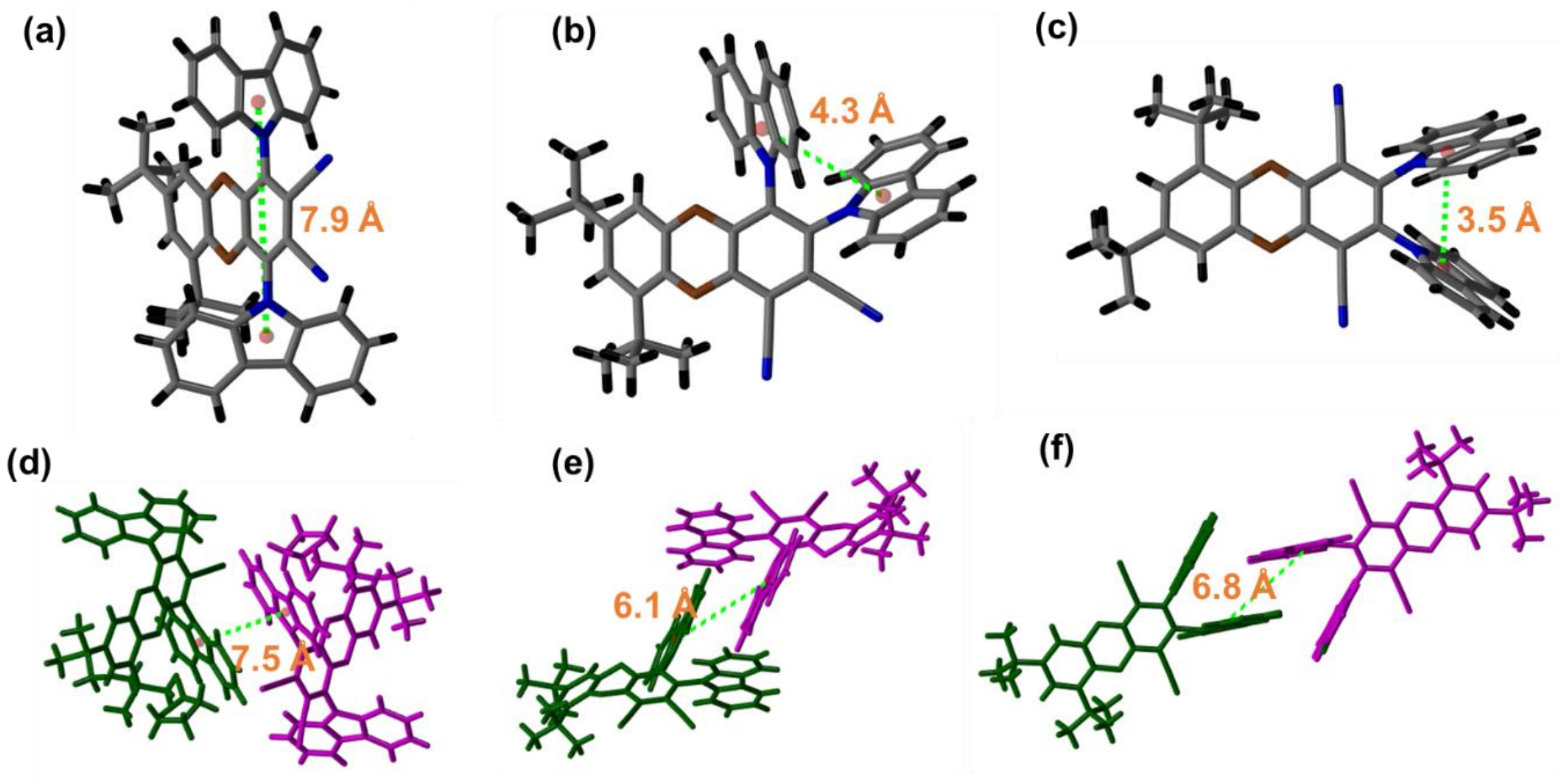

Fig. S21 Intramolecular and Intermolecular π···π stacking interactions distances between carbazole rings in (a), (d) for ***o*CN1**, (b), (e) for ***o*CN2**, and (c), (f) for ***p*CN**. Different color codes represent two different molecules.

Crystals Emissions and Lifetimes:

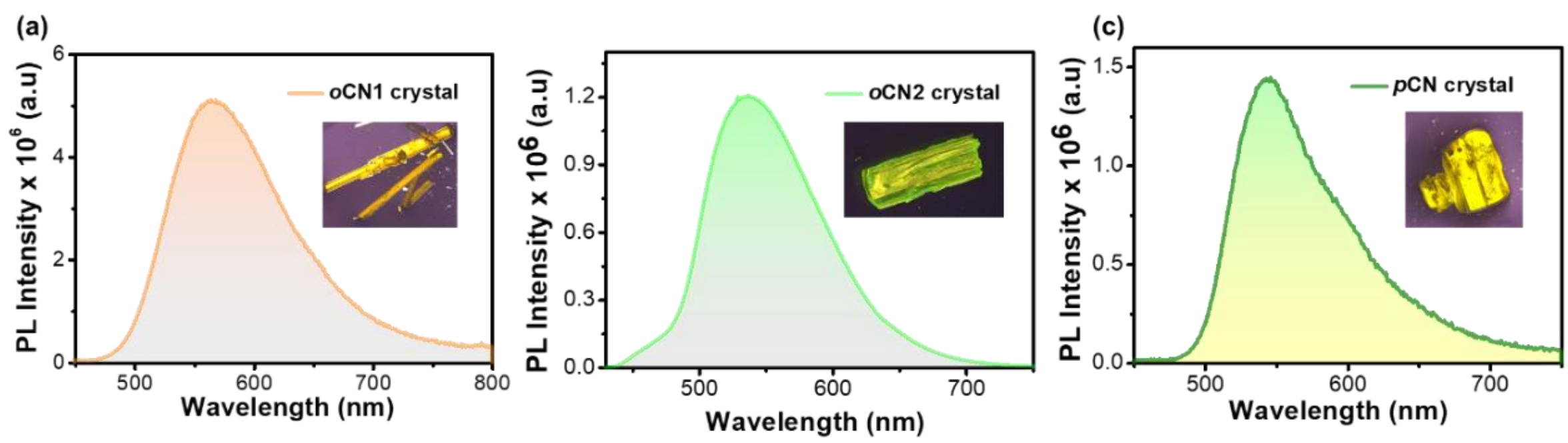

Fig. S22 Steady-state emission spectra of the crystals of (a) ***o*CN1**, (b) ***o*CN2**, and (c) ***p*CN** along with the UV (365 nm) excited crystal emission shown in the insets.

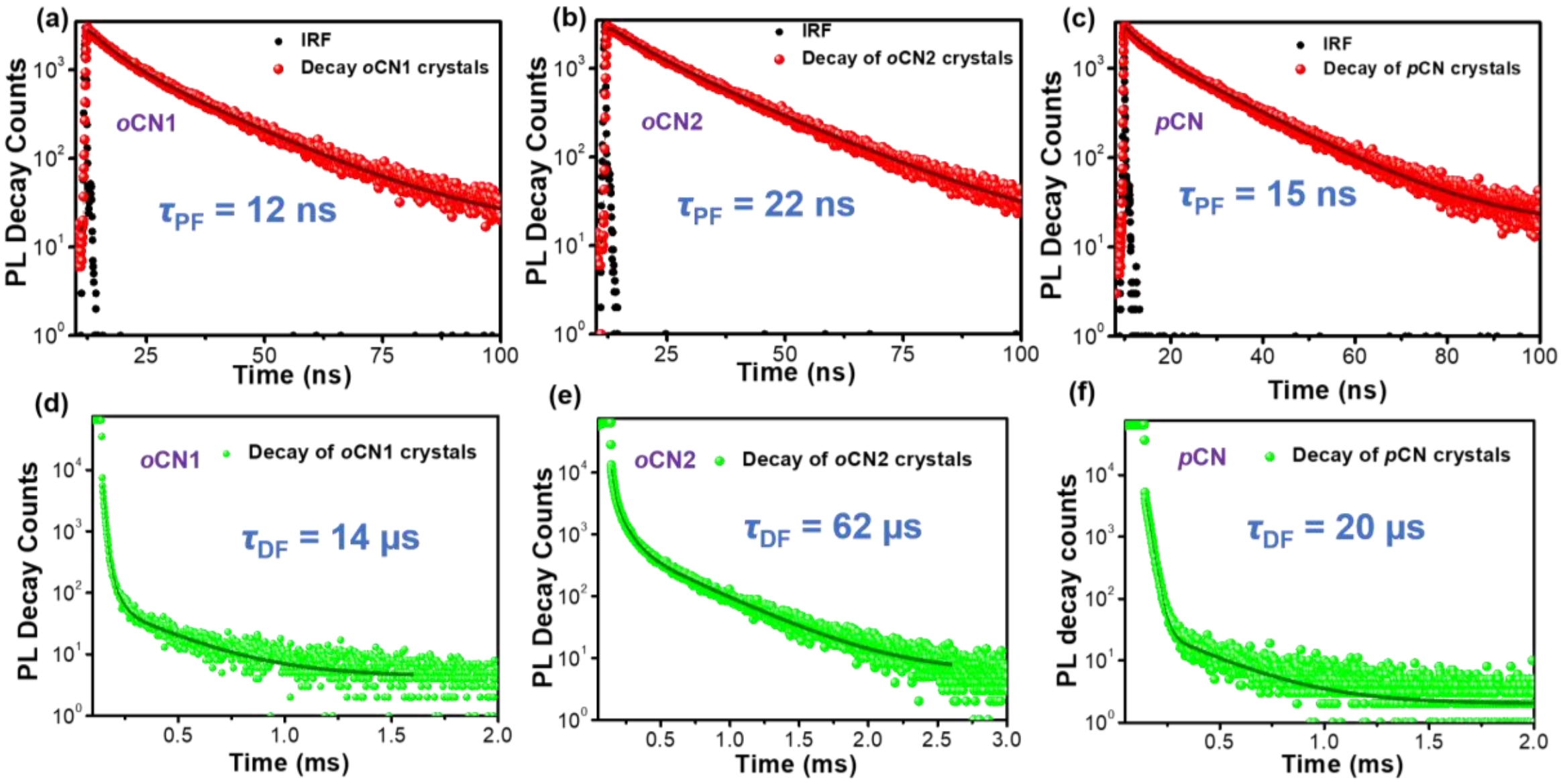

Fig. S23 Time-resolved emission decay profiles for the crystals of (a) ***o*CN1**, (b) ***o*CN2** and (c) ***p*CN** in the nanosecond time scale (λex = 402 nm) and (d) ***o*CN1**, (e) ***o*CN2** and (f) ***p*CN** in the sub-millisecond time scale (λex = 366 nm).

Crystals Packing arrangements (Space-filling model):

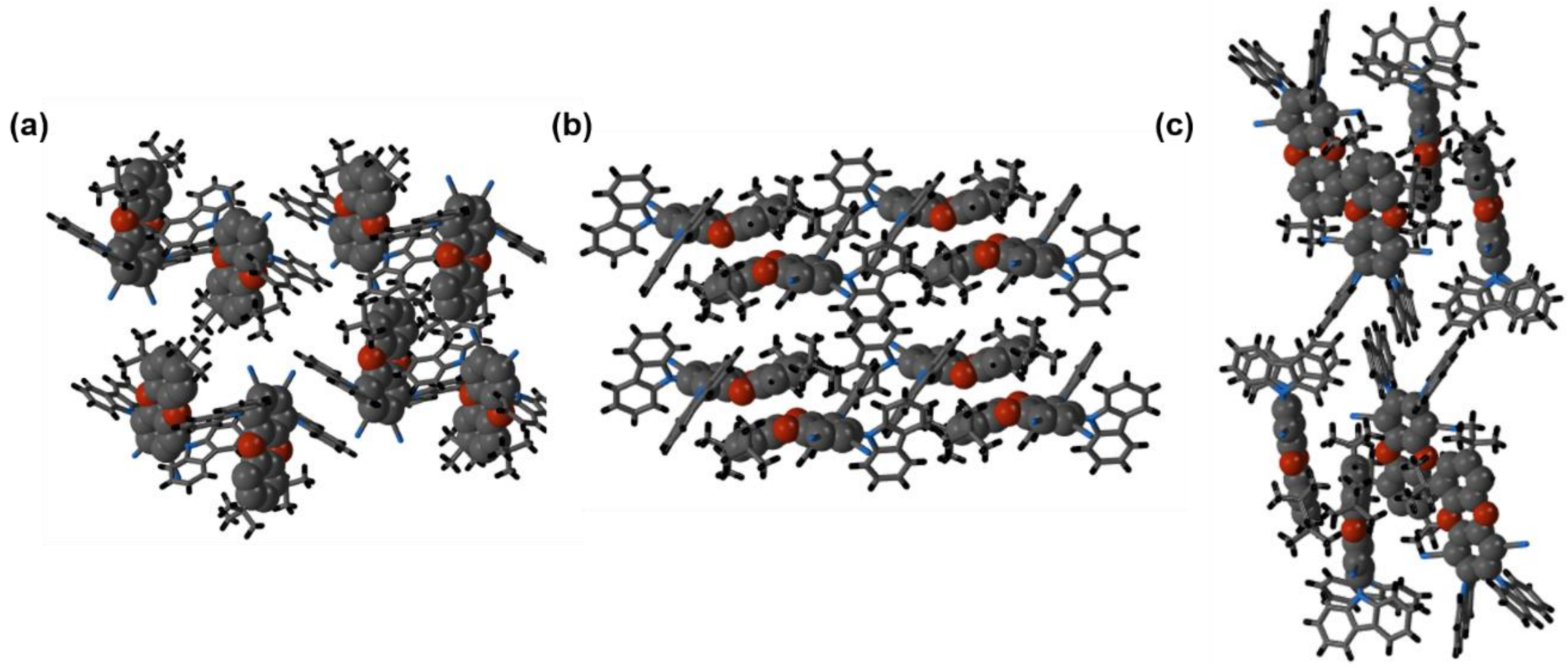

Fig. S24 Space filling model of variant types of molecular packing for (a) *o*CN1, (b) *o*CN2 and (c) *p*CN crystals.

Hirshfield Surface Analysis:

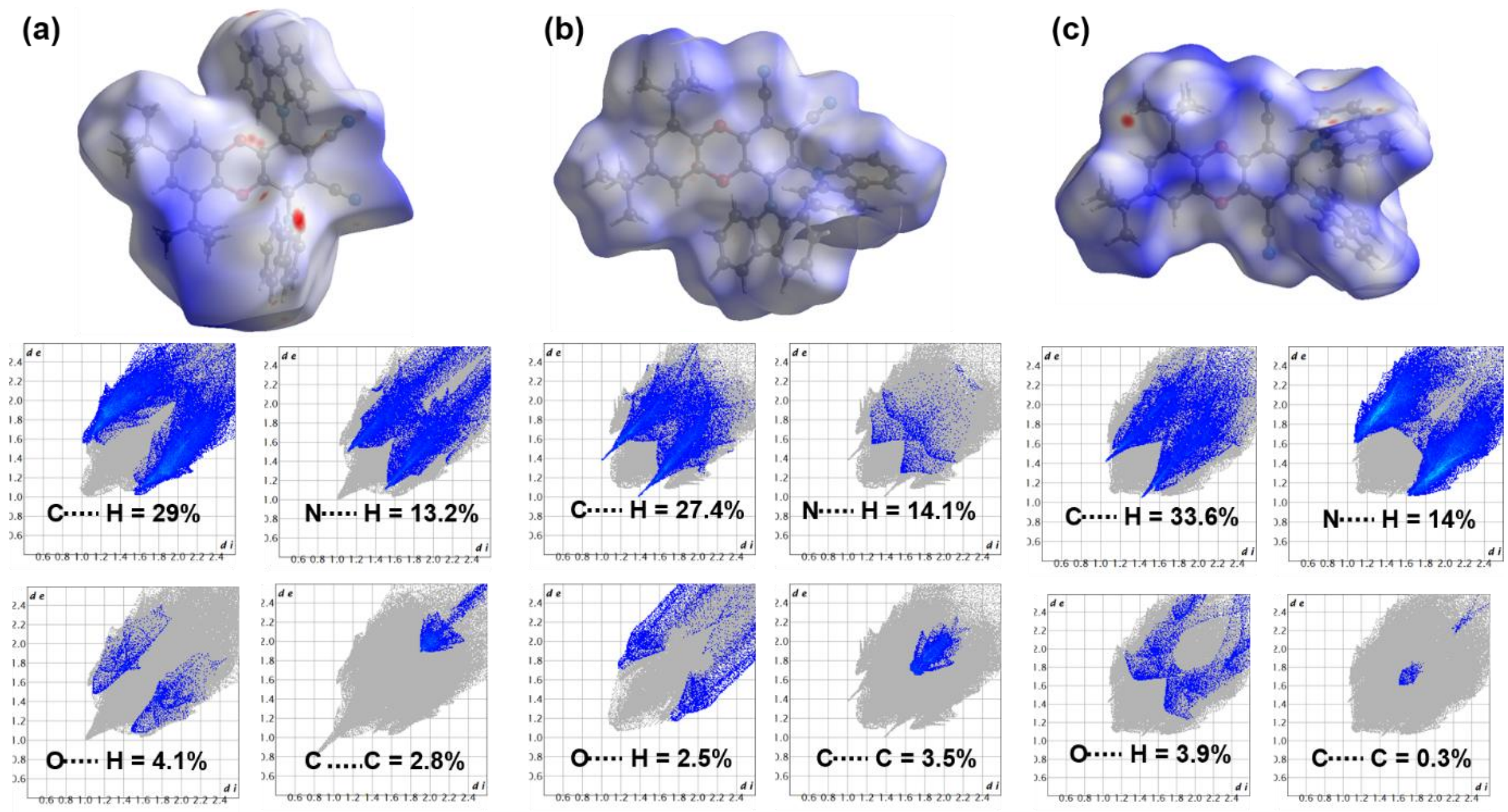

Fig. S25 Normalized distance (d_{norm}) mapped over Hirshfeld surface of each luminogen ((a) ***o*CN1**, (b) ***o*CN2** and (c) ***p*CN**) along with generated finger print plots obtained from d_{norm} to decipher percentage of each type of non-covalent interaction. The grey part in the finger print plot indicates total interactions.

The Hirshfeld surface is a method for partitioning crystal electron density into molecular fragments, defining smooth boundaries around molecules and enabling visualization of their intermolecular interactions.[11-12] d_e and d_i are the two interactions to be mapped on the Hirshfeld surface; where d_e is the distance of an atom external to the generated Hirshfeld surface and d_i is the distance of an atom internal to the Hirshfeld surface. These two values together generate a 2-D fingerprint plot where different colors indicate the frequency of occurrence of interaction. In this regard, the normalized contact distance based on de, di, and the van der Waals radii of the atom enables the identification of the region of importance to intermolecular interactions. The d_{norm} values are mapped onto the Hirshfeld surface by a red-white-blue color scheme, where the red color indicates closer contact and negative d_{norm} value, the blue region suggests longer contact and positive d_{norm} value, and the white color indicates the distance of contact exactly equal to the van der Waals separation and a d_{norm} value equals to zero.[11-13] Here in this work, the Hirshfeld surface of all the designed luminogens is generated using Crystal Explorer 3.1 software with an iso-value of 0.5.[14]

Applications:

Section S8: Fabrication of converted LEDs:

Commercially available InGaN chips (power = 3W, λ_{EL}= 390-395 nm, VF = 3.1 V, IF = 700 mA) were purchased from ASIAN ELECTRONICS, India (**Fig. S25†**). The optimal sample, at 10 wt% doped with poly (methyl methacrylate) or PMMA was mixed in chloroform and heated at 50°C with stirring for 10 minutes. After that, the mixture was coated on top of the UV-chip, dried under an IR lamp source, and was used further for emission measurements. Then the emission spectra corresponds to three c-LEDs (*o*CN1, *o*CN2 and *p*CN) had been collected at Fluoromax-4C instrument with minimum excitation monochromator slit width.

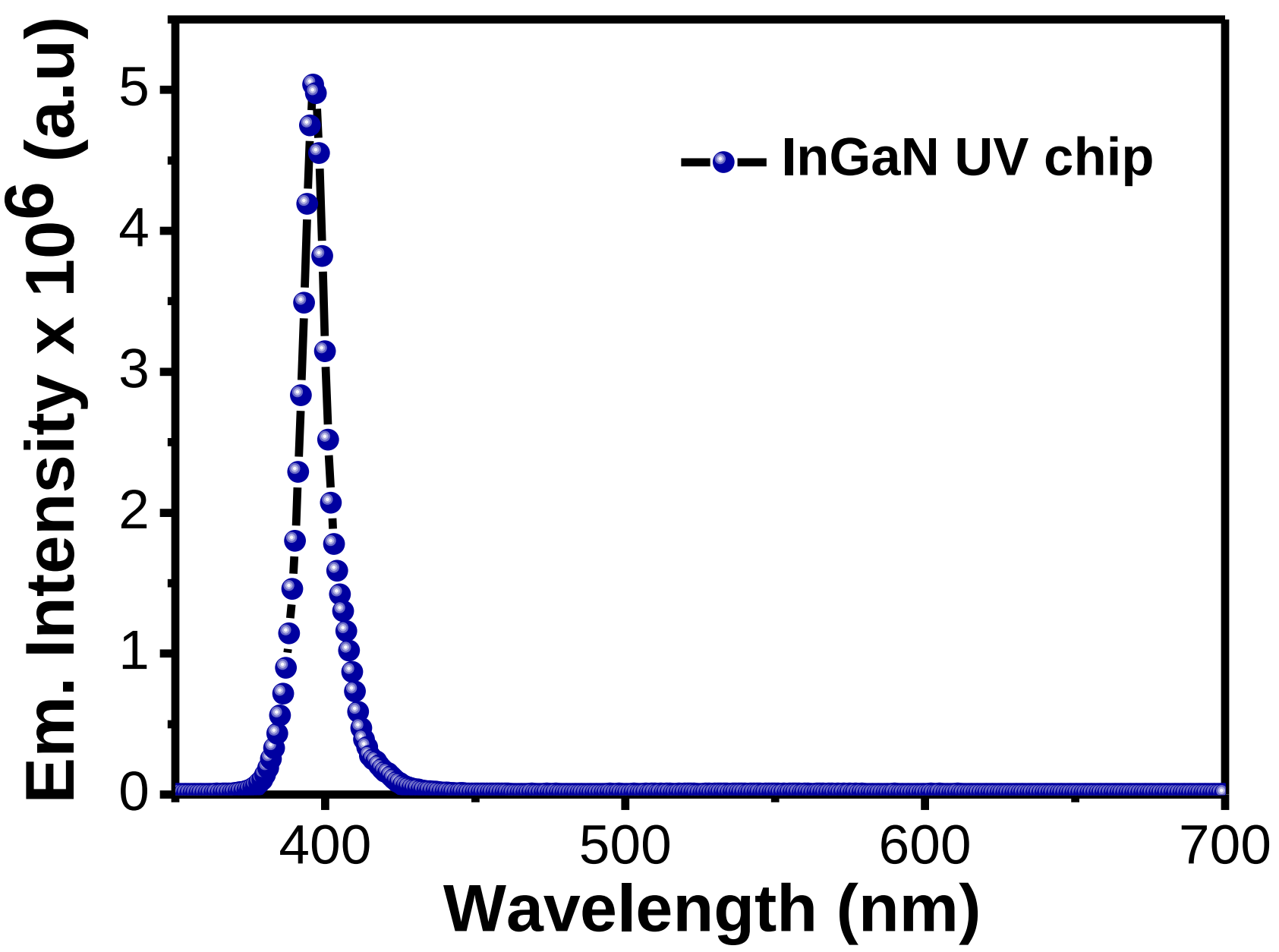

Fig. S26 Electroluminescence spectra of the InGaN chip centered at 390-395 nm region.

Section S9: Two -photon absorption properties

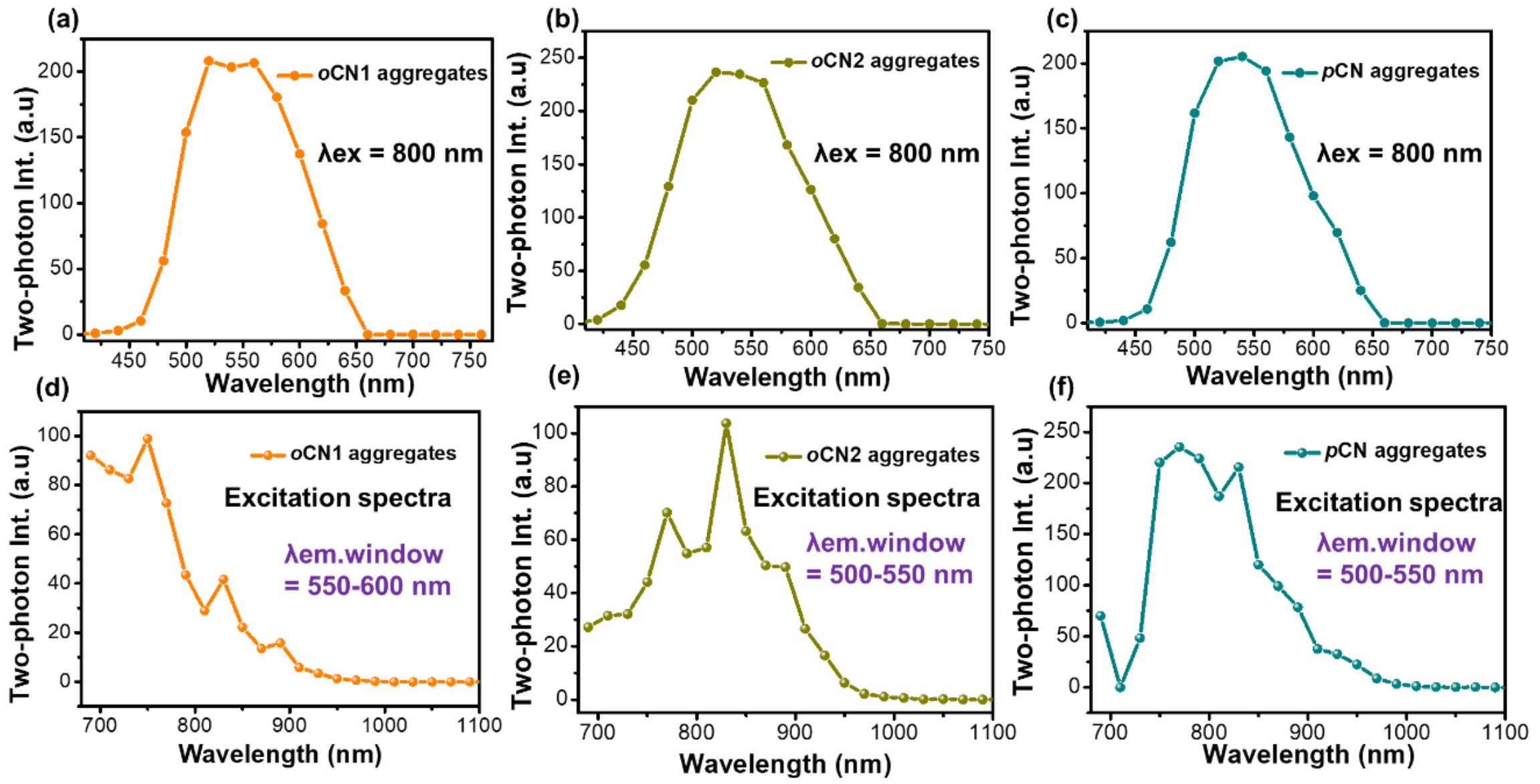

Fig. S27 Two-photon excited emission spectra (excitation wavelength = 800 nm and power = 116 mW) of (a) ***o*CN1**, b*)* ***o*CN2** and (c) ***p*CN** aggregates (formed at 95% water- 5% THF binary mixtures). Two-photon excitation spectra for (d) ***o*CN1** (emission range= 550-600 nm), (e) ***o*CN2** (emission range 500-550 nm) and (f) ***p*CN** (emission range= 500-550 nm).

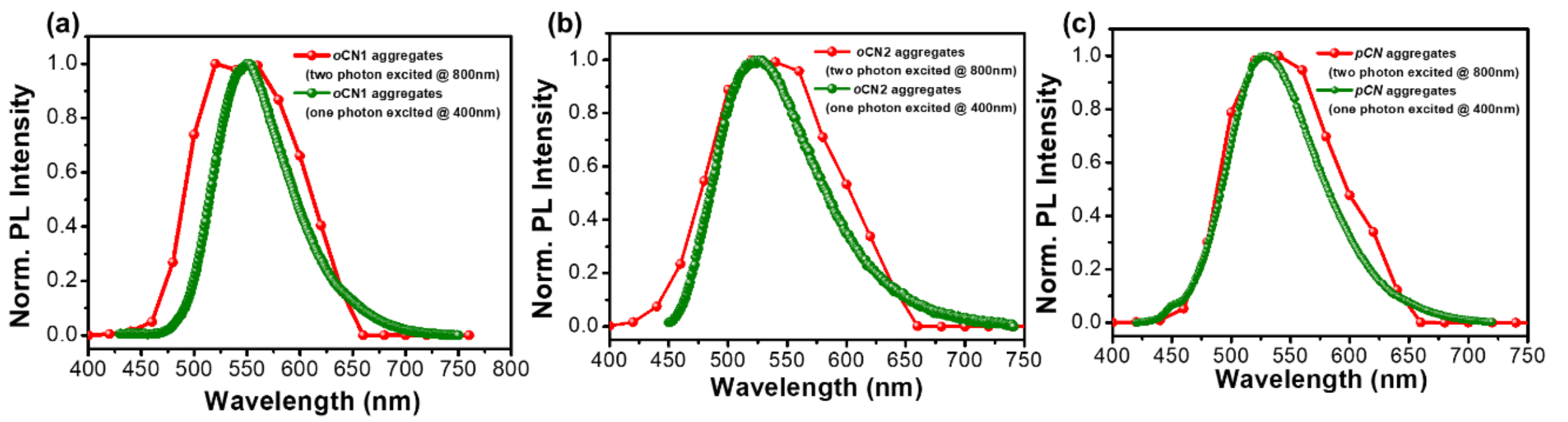

Fig. S28 One-photon and two-photon emission profile of (a) ***o*CN1**, (b) ***o*CN2** and (c) ***p*CN**.

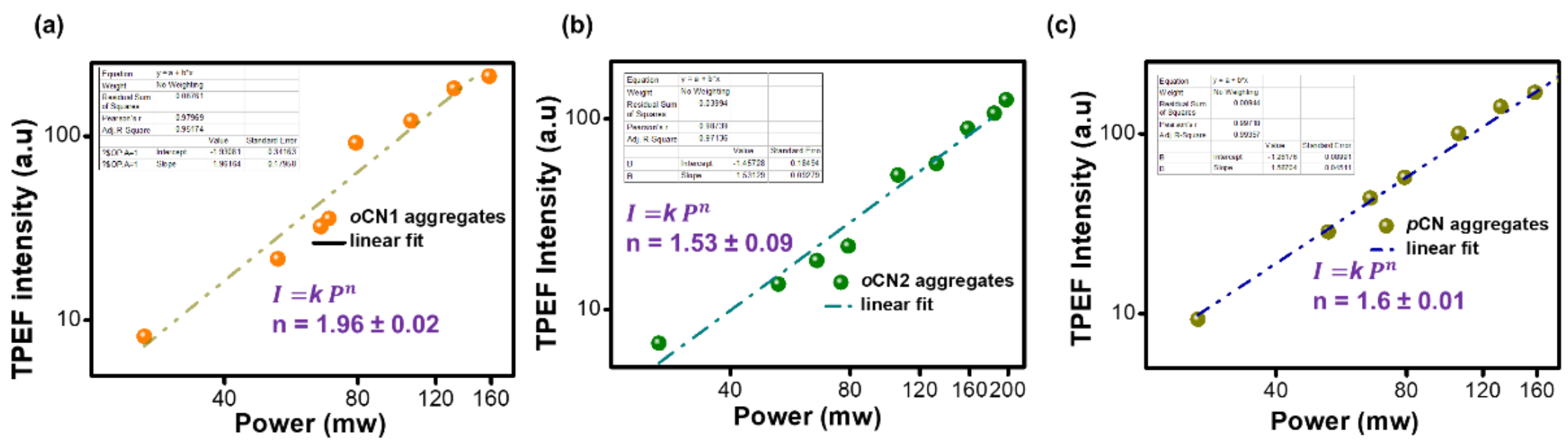

Fig. S29 Power dependence study (excitation = 800 nm) of (a) ***o*CN1**, (b) ***o*CN2** and (c) ***p*CN**.

Section S10: Two photon imaging studies

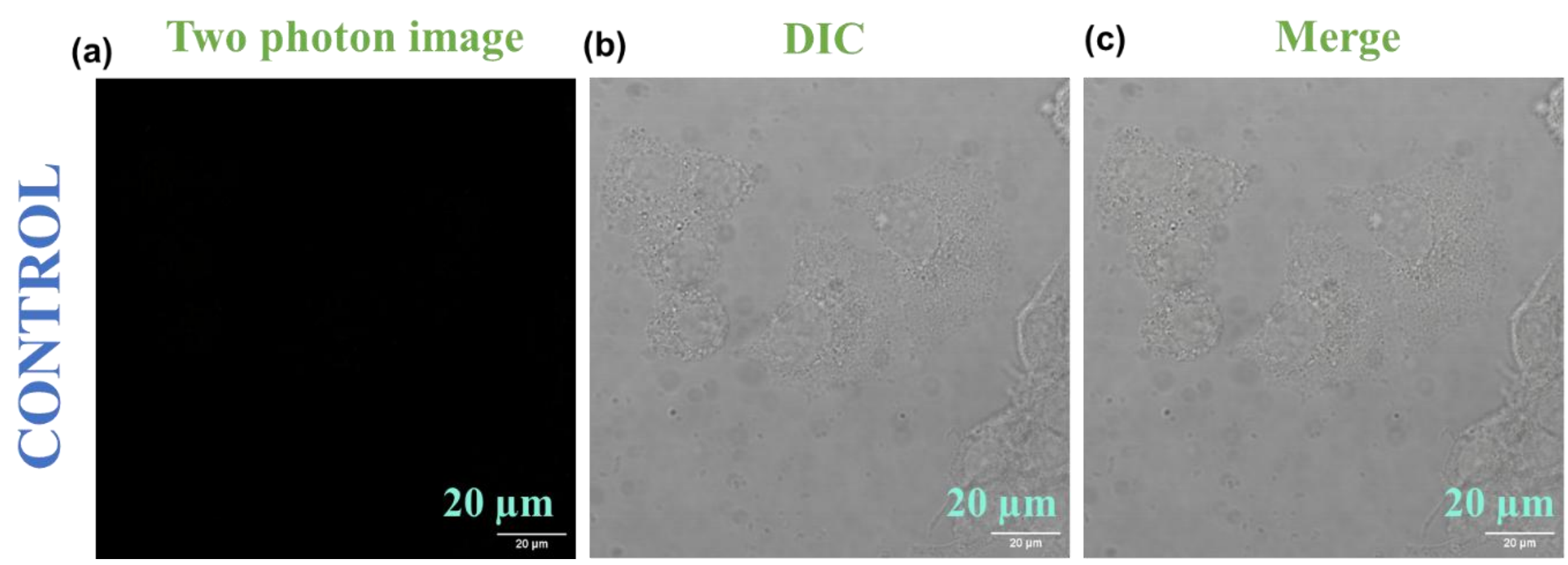

Fig. S30 Control experiment for two-photon microscopy imaging of MCF7 cells. (a) cells treated with DMSO without dye, (b) Differential interference contrast (DIC) image, (c) Merged image of DIC image and cells treated with DMSO without dye.

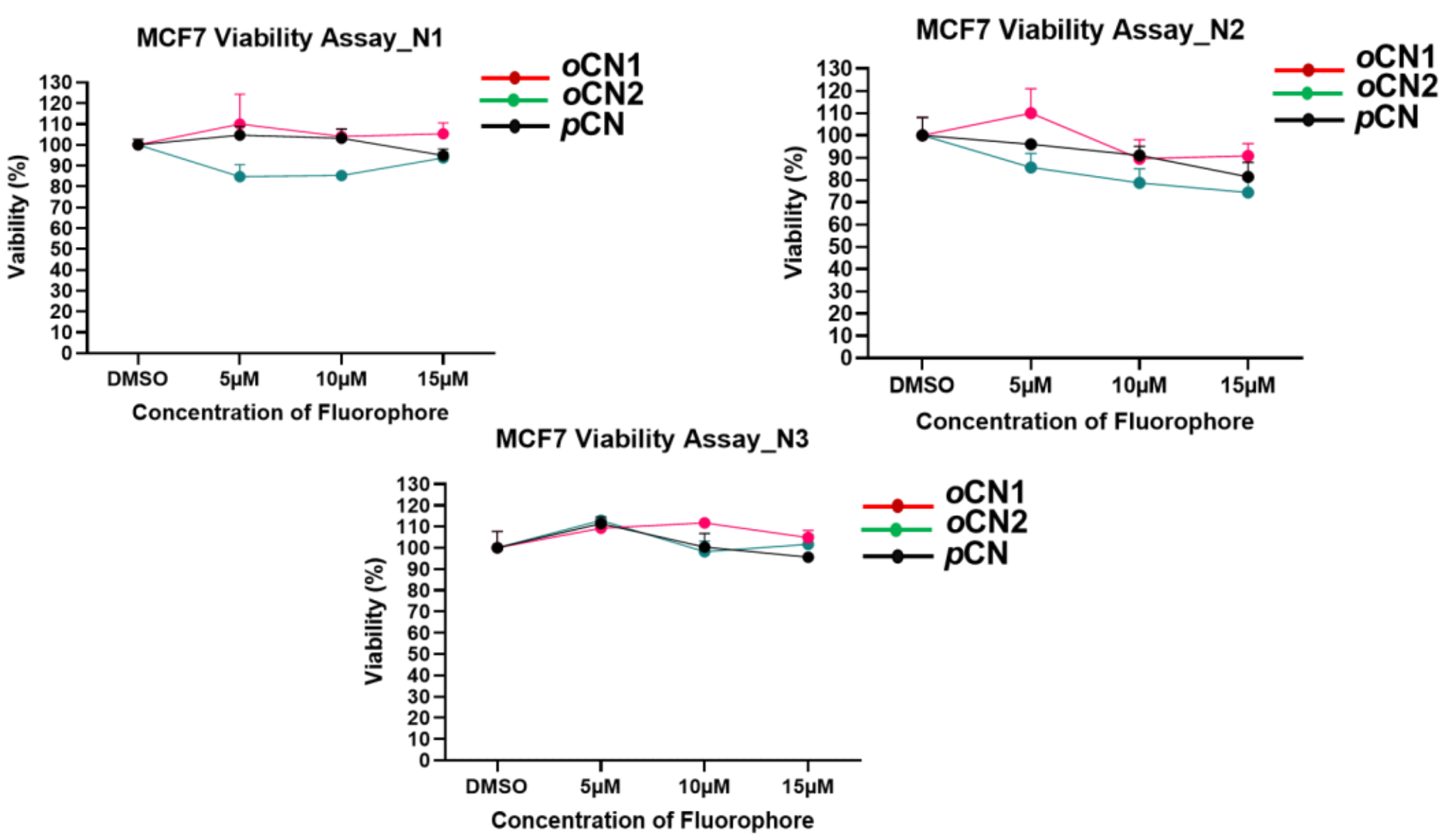

Fig. S31 MTT-cell viability assay of MCF-7 cells in the presence of different concentrations (5 µm, 10 µm and 15 µm) of dye (***o*CN1, *o*CN2 and *p*CN**) across three biological replicates**.**

Table S6 PLQYs in neat films and 95% aggregates of reported literature:

Structures	Name	PLQY in neat film (%)	PLQY in 95% aggregates (%)	References
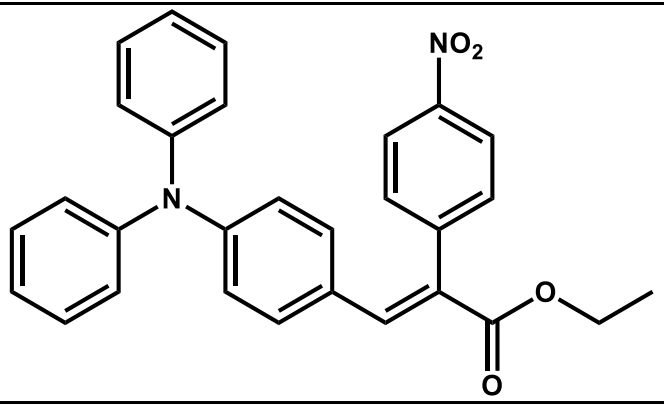	TN		13	15
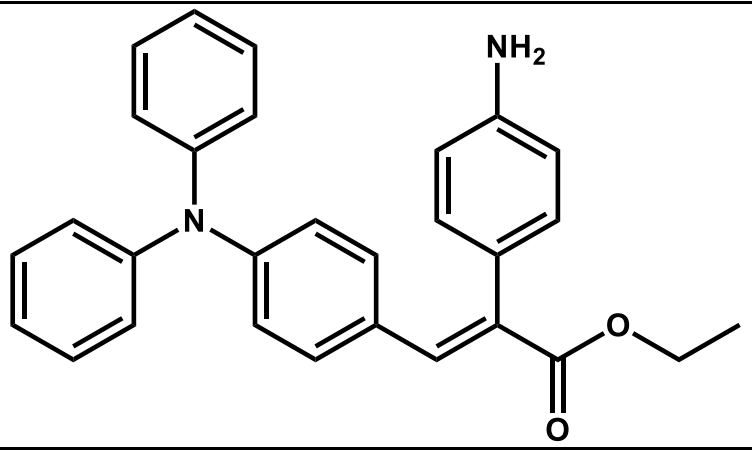	TA		7	15
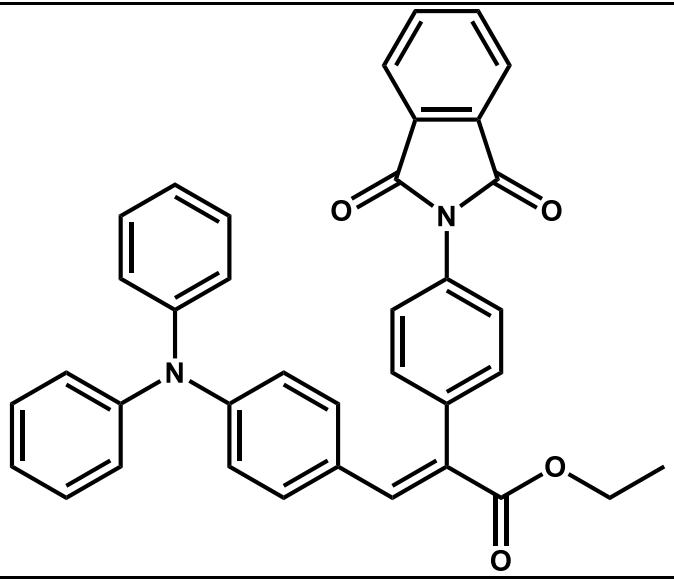	TP		45	15
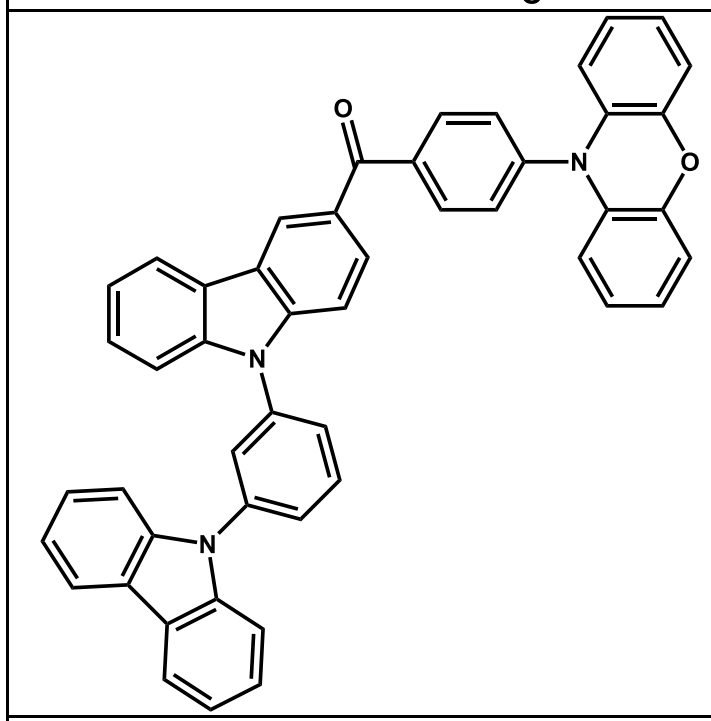	mCP-BP-PXZ		62	16
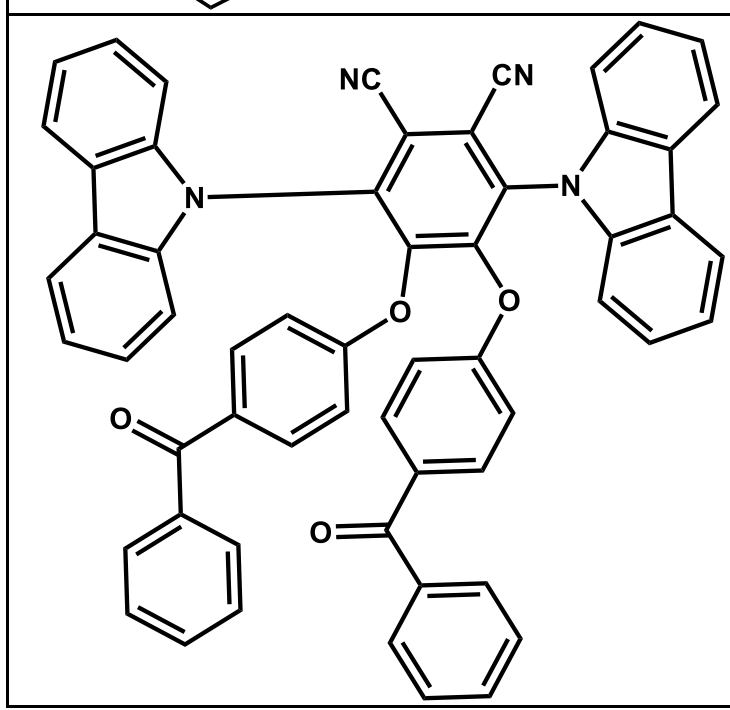	CBPN	33		17

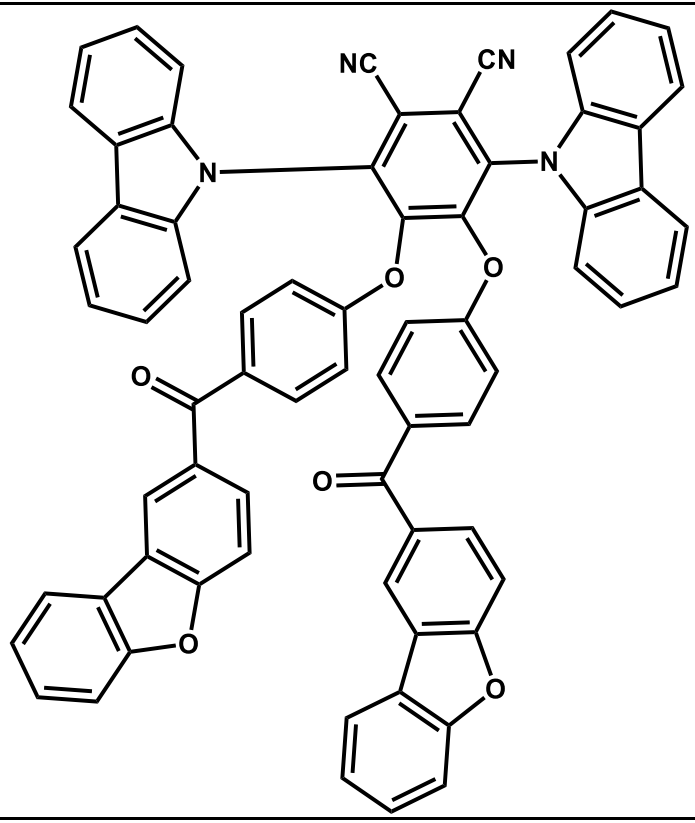	CDBFPN	67	48	17
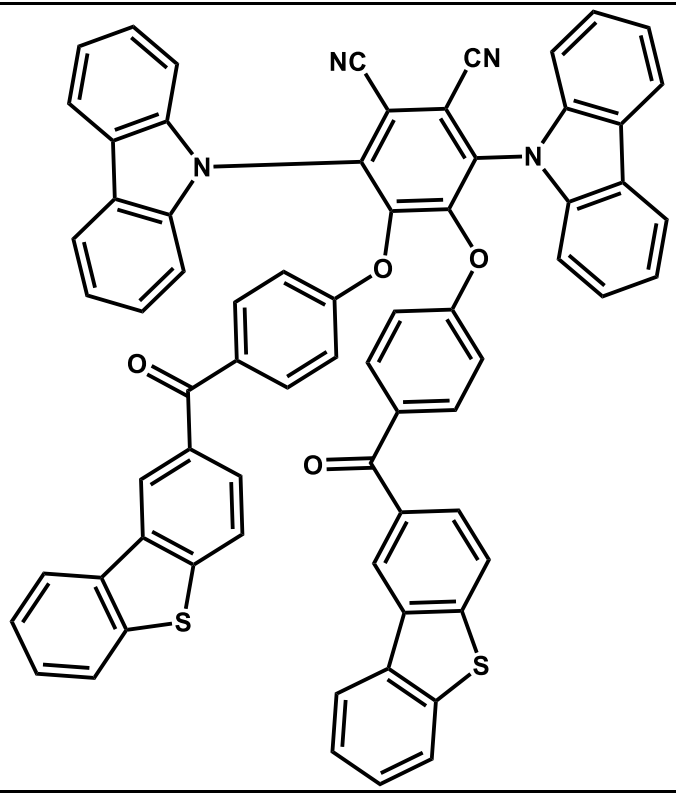	CDBTPN	60	54	17
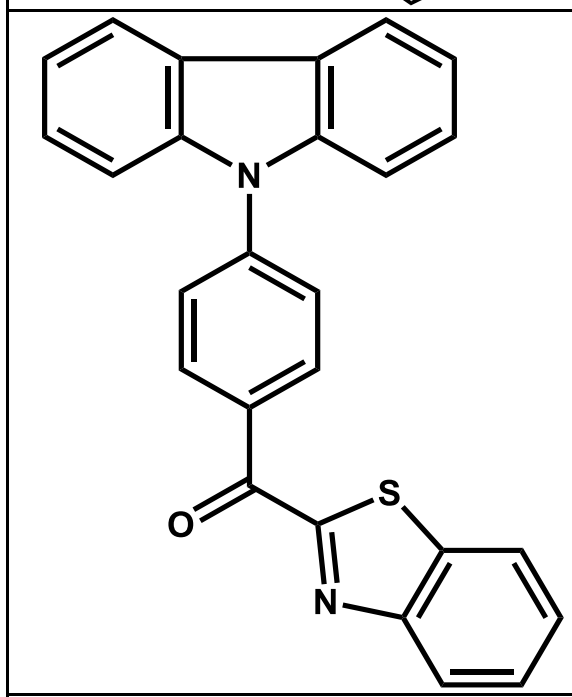	BTM-Cz	62	59	18
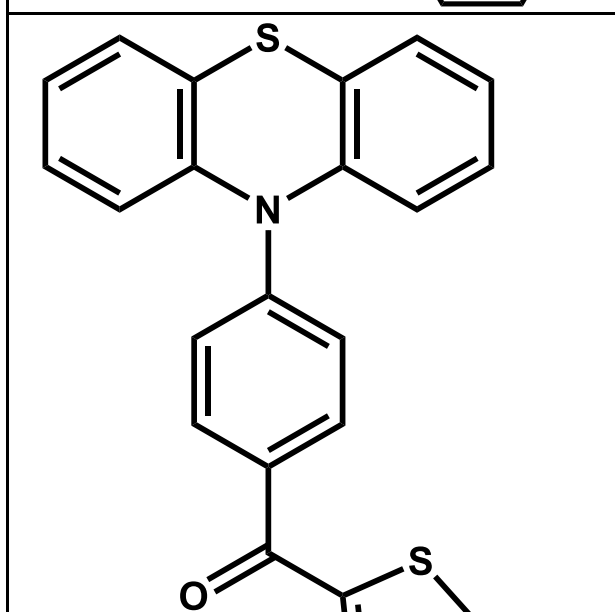	BTM-PTZ	8	6	18

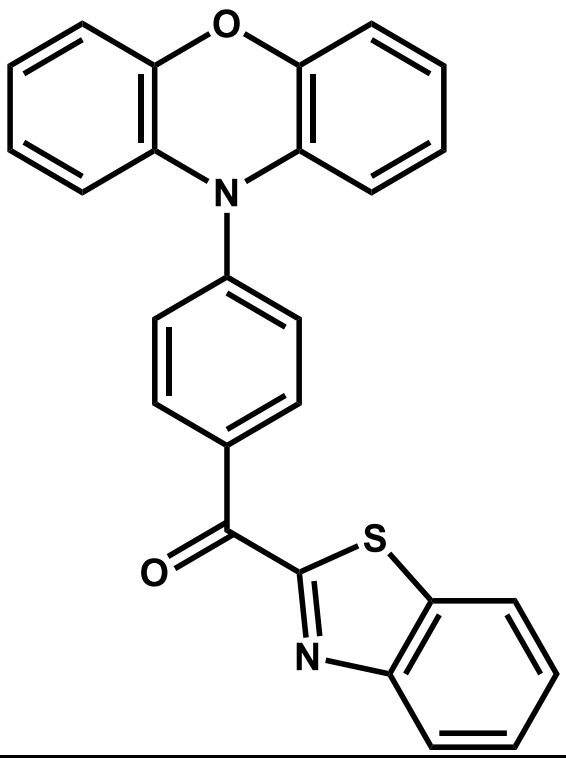	BTM-PXZ	5	4	18
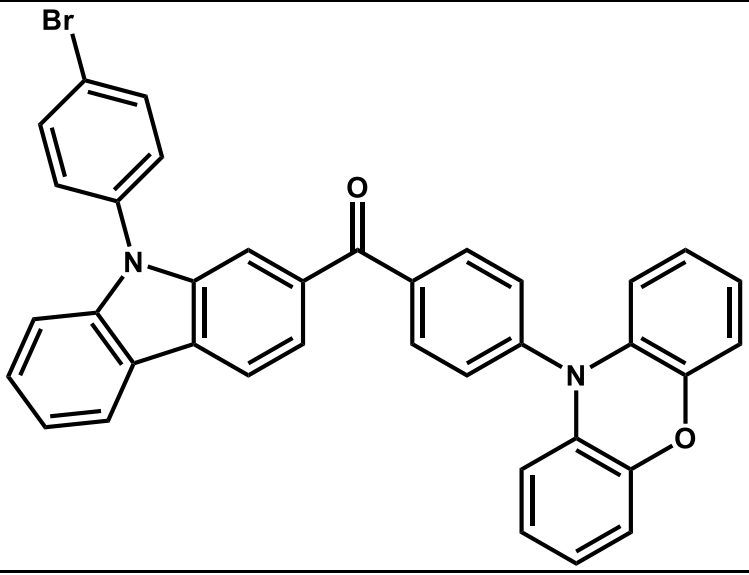	9-BCP-BP-PXZ	53		19
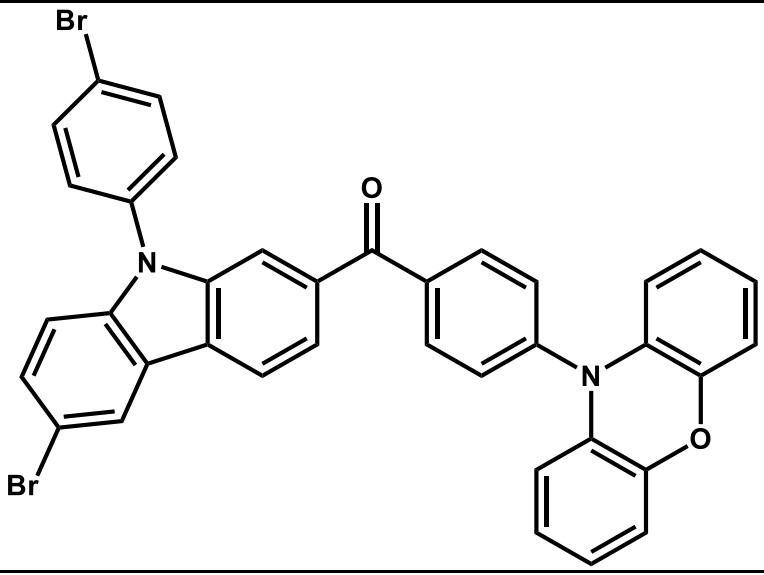	3,9-BCP-BP-PXZ	50		19
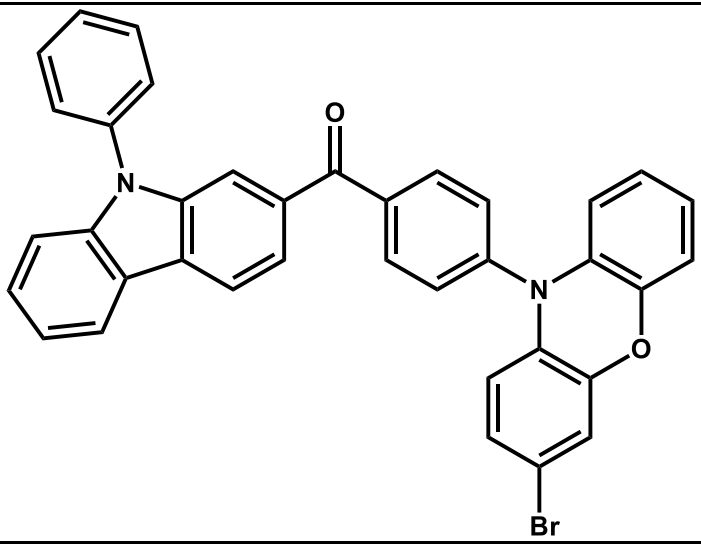	CP-BP-BPXZ	16		19
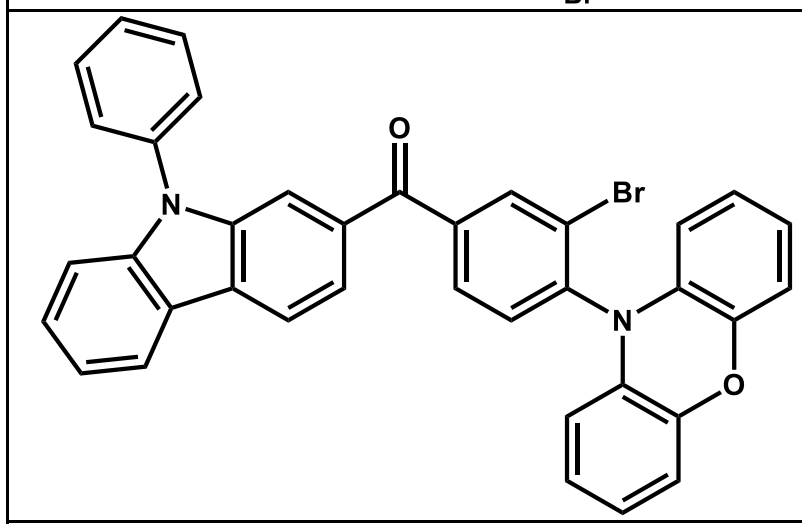	CP-3-BBP-PXZ	23		19
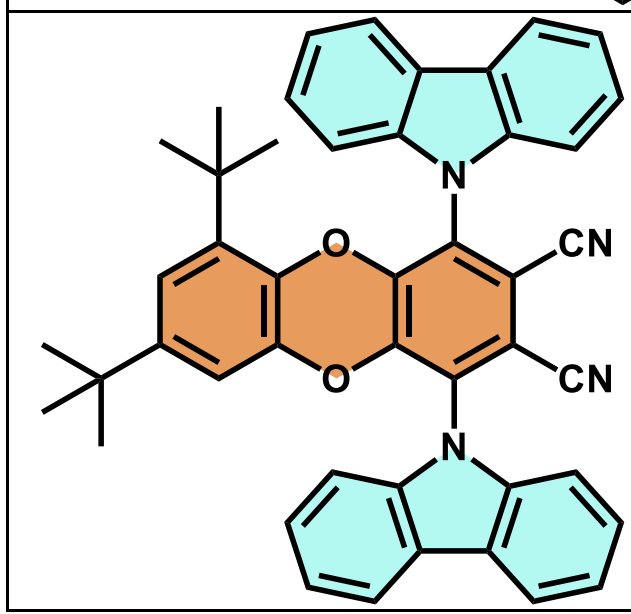	**_o_CN1**	**78**	**63**	This work

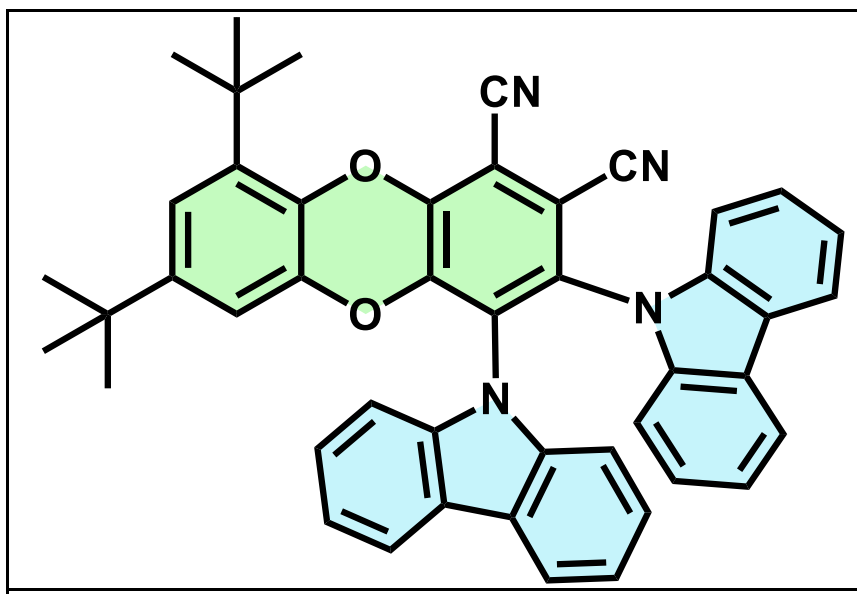	***o*CN2**	**31**	**40**	This work
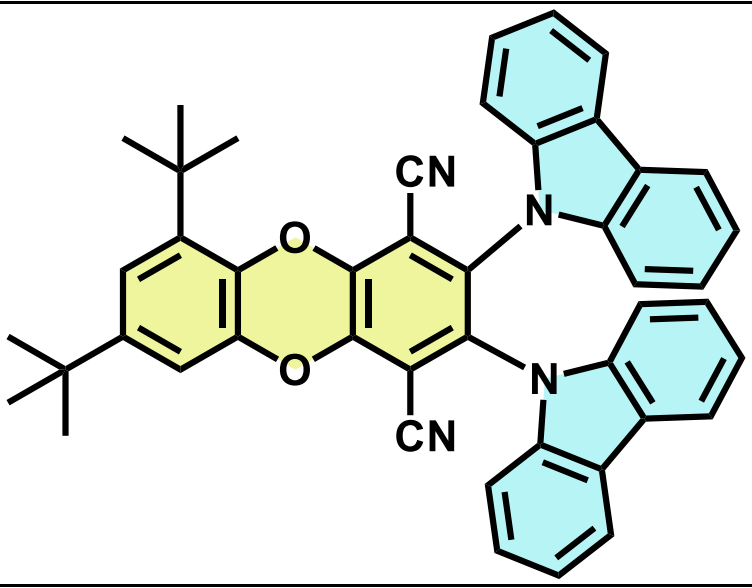	***p*CN**	**54**	**52**	This work

References:

1 G. M. Sheldrick, *Act. Cryst. A.*, 2008, **64**, 112.

2 G. M. Sheldrick, *Act. Cryst. Sec. C.*, 2015, **71**, 3.

3 M. J. Frisch, G. W. Trucks, H. B. Schlegel, G. E. Scuseria, M. A. Robb, J. R. Cheeseman, G. Scalmani, V. Barone, G. A. Petersson, H. Nakatsuji, X. Li, M. Caricato, A. V. Marenich, J. Bloino, B. G. Janesko, R. Gomperts, B. Mennucci, H. P. Hratchian, J. V. Ortiz, A. F. Izmaylov, J. L. Sonnenberg, D. Williams-Young, F. Ding, F. Lipparini, F. Egidi, J. Goings, B. Peng, A. Petrone, T. Henderson, D. Ranasinghe, V. G. Zakrzewski, J. Gao, N. Rega, G. Zheng, W. Liang, M. Hada, M. Ehara, K. Toyota, R. Fukuda, J. Hasegawa, M. Ishida, T. Nakajima, Y. Honda, O. Kitao, H. Nakai, T. Vreven, K. Throssell, J. A. Montgomery, Jr., J. E. Peralta, F. Ogliaro, M. J. Bearpark, J. J. Heyd, E. N. Brothers, K. N. Kudin, V. N. Staroverov, T. A. Keith, R. Kobayashi, J. Normand, K. Raghavachari, A. P. Rendell, J. C. Burant, S. S. Iyengar, J. Tomasi, M. Cossi, J. M. Millam, M. Klene, C. Adamo, R. Cammi, J. W. Ochterski, R. L. Martin, K. Morokuma, O. Farkas, J. B. Foresman and D. J. Fox, *Gaussian 09, Revision C.01, Wallingford, CT*, 2009.

4 A. D. Becke, *Phys. Rev. A.*, 1988, **38**, 3098.

5 C. Lee, W. Yang and R. G. Parr, *Phys. Rev. B., 1988*, **37**, 785.

6 T. Vreven, K. Morokuma, O. Farkas, H. B. Schlegel and M. J. Frisch, *J. Comput. Chem.*, 2003, **24**, 760.

7 H. Lin, D. G. Truhlar, *Theor. Chem. Acc*., 2007, **117**, 185.

8 S. V. K. Isukapalli, R. S. Lekshmi, P. K. Samanta and S. R. Vennapusa, *J. Chem. Phys.*, 2020, **153**, 124301.

9 M. Homocianu, A. Airinei, A. M. Ipate and C. Hamciuc, *Chemosensors*, 2022, **10**, 183.

10 T. Lu and F. Chen and *J. Comput. Chem*., 2012, **33**, 580.

11 A. D. Martin, J. Britton, T. L. Easun, A. J. Blake, W. Lewis and M. Schröder, *Cryst. Growth Des*., 2015, **15**, 1697.

12 A. D. Martin, K. J. Hartlieb, A. N. Sobolev and C. L. Raston, *Cryst. Growth Des.*, 2010, **10**, 5302.

13 J. J. McKinnon, D. Jayatilaka and M. A. Spackman, *Chem. Commun*., 2007, **37**, 3814.

14 P. R. Spackman, M. J. Turner, J. J. McKinnon, S. K. Wolff, D. J. Grimwood, D. Jayatilaka and M. A. Spackman, *J. Appl. Crystallogr*., 2021, **54**, 1006.

15 A. Chatterjee, S. Narayanan, S. Thorat, A. J. Malik, M. D. Ambhore, A. Narayanan, A. K. Sihag, S. S. Babu, M. Lahiri and P. Hazra, *Chem. Commun***.**, 2025, **61**, 1447.

16 B. Zhang, Y. Kong, H. Liu, B. Chen, B. Zhao, Y. Luo, L. Chen, Y. Zhang, D. Han, B. Z. Tang and L. Niu, *Chem. Sci.*, 2021, **12**, 13283.

17 M. Upadhyay and D. Ray, *Chem. Commun***.**, 2025, **61**, 5015.

18 D. Barman, P. Rajamalli, A. P. Bidkar, T. Sarmah, S. S. Ghosh, E. Zysman-Colman and P. K. Iyer, *Small*, 2025, **21**, 2409533.

19 J. Xu, X. Wu, J. Li, Z. Zhao and B. Z.Tang, *Adv. Opt. Mater*., 2022, **10**, 2102568.
